# Disrupting Notch signalling by a small molecule inhibiting dihydroorotate dehydrogenase activity

**DOI:** 10.1038/s41598-026-55679-3

**Published:** 2026-06-06

**Authors:** Eike-Benjamin Braune, Dirk Wienke, Anita Seshire, Timo Heinrich, Martin Haraldsson, Sonia Lain, Urban Lendahl

**Affiliations:** 1 Department of Oncology-Pathology, Karolinska Institutet, Stockholm, Sweden; 2 Merck Healthcare KGaA, Darmstadt, Germany; 3 Department of Medical Biochemistry and Biophysics, Karolinska Institutet, Stockholm, Sweden; 4 Department of Microbiology, Tumor and Cell Biology, Karolinska Institutet, Stockholm, Sweden; 5 Department of Cell and Molecular Biology, Karolinska Institutet, Stockholm, Sweden

**Keywords:** Biochemistry, Cancer, Cell biology, Computational biology and bioinformatics, Drug discovery

## Abstract

The Notch signalling pathway is highly evolutionarily conserved and regulates differentiation and homeostasis in most organs. Given the critical role of Notch signalling for normal development, dysregulated Notch signalling is frequently linked to pathogenesis of disease and cancer. Hence, developing Notch-targeting therapeutics is warranted but has been challenging and Notch inhibitors have not yet reached broad clinical use. In this report, we identify potential Notch inhibitors, using a novel cell-based Notch reporter system for unbiased screening of compounds reducing Notch signalling. A library of 37,966 small organic compounds was screened for inhibitor candidates, followed by a counter screen to eliminate γ-secretase inhibitor-like compounds and an orthogonal screen based on the role of Notch signalling in myogenic differentiation. This triage led to the identification of five Notch inhibitor candidate hits with different chemical backbones and unrelated to previous Notch antagonists. One candidate hit proved to be a DHODH inhibitor, and we also provide evidence that a well-established DHODH inhibitor is a highly potent Notch inhibitor. In conclusion, our data support the notion that DHODH inhibition may be an interesting avenue to explore for the development of novel Notch inhibitors.

**Supplementary Information:**

The online version contains supplementary material available at 10.1038/s41598-026-55679-3.

## Introduction

The Notch signalling pathway is a cell–cell communication system required for differentiation and homeostasis of most tissues and organs. Notch receptors and ligands are highly evolutionarily conserved, and Notch signalling operates in most, if not all, multicellular organisms^1,2^. In most tissues, Notch signalling promotes an undifferentiated cell state and blocks differentiation, while in some organs, such as the skin, Notch signalling drives differentiation.

**Fig. 1:**
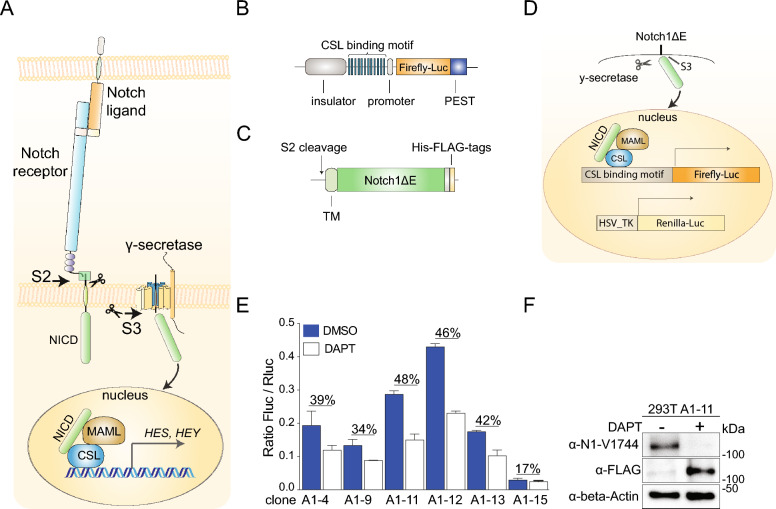
A novel Notch reporter system. (**A**) Schematic overview of the Notch signalling pathway. Arrows mark the S2 and S3 cleavage sites, and the ternary Notch ICD/MAML/CSL transcriptional complex in the nucleus is depicted. (**B**) Schematic depiction of the novel Notch reporter construct. (**C**) The Notch1ΔE construct used to generate enhanced ligand-independent Notch signalling in the reporter cell line. The S2 cleavage site, transmembrane domain (TM) and C-terminal protein tags are indicated. (**D**) Schematic depiction of the reporter cell line, containing the Notch1ΔE construct, the CSL-firefly luciferase reporter to read out Notch signalling, and the constitutively activated HSV-TK renilla-luciferase as an internal control for luciferase activity. (**E**) Analysis of the levels of firefly/renilla luciferase activity in different clones of the HEK293T reporter cell line. (**F**) Western blot analysis of Notch1ΔE in clone #A1-11 in the presence or absence of DAPT, visualized by an anti-FLAG (a-FLAG) antibody or an antibody recognizing the S3-cleaved form of NOTCH1 (a-N1-V1744), as indicated. Beta-Actin was used as loading control. Samples were run on the same gel and the anti-N1-V1744 membrane was stripped and reprobed with anti-FLAG antibody.

Mechanistically, Notch signalling is initiated when a transmembrane Notch receptor interacts with a transmembrane Notch ligand of the Jagged or Delta-like type presented on a juxtaposed cell (Fig. 1A). Ligand-receptor interaction leads to proteolytic processing of the Notch receptor by ADAM10 at the extracellular side close to the plasma membrane (referred to as S2-cleavage). S2-cleavage is rapidly followed by S3-cleavage, which is executed by the γ-secretase complex at the intracellular surface of the plasma membrane or in endosomes (Fig. 1A). S3-cleavage liberates the C-terminal portion of the Notch receptor, i.e., the Notch intracellular domain (Notch ICD), which translocates to the cell nucleus. In the cell nucleus, Notch ICD interacts with the DNA-binding protein CSL and an adaptor protein MAML. The ternary Notch ICD/MAML/CSL complex regulates transcription of downstream genes, including *Nrarp*, *Hes* and *Hey* (Fig. 1A).

In accordance with its importance for normal cellular differentiation, aberrant Notch signalling is observed in disease and cancer. Several monogenic diseases are caused by mutations in genes in the Notch signalling pathway, including a number of heart diseases, the multiorgan disease Alagille syndrome, the stroke and dementia syndrome CADASIL and the connective tissue disorder Hajdu-Cheney disease^2^ A considerable number of cancers, including acute lymphoblastic T-cell leukaemia (T-ALL), adenoid cystic carcinoma (ACC), triple negative breast cancer (TNBC) and non-small cell lung cancer (NSCLC) are linked to hyperactive Notch signalling. There are however also tumour forms in which Notch serves as a tumour suppressor, including small cell lung cancer (SCLC), bladder cancer and squamous cell carcinoma (SCC)^3–5^.

The observation that elevated Notch signalling, caused by Notch receptor gain-of-function mutations or hyperactivation of Notch signalling as an indirect consequence of other mutations, is found in several cancers and diseases has motivated efforts to develop Notch-targeting therapies. There have been several attempts to generate Notch inhibitors, and there are > 400 clinical trials with preclinical Notch inhibitors reported at clincialtrial.com. γ-secretase inhibitors (GSIs), which block the activity of the γ-secretase complex and thus S3-cleavage of all Notch receptors, have however caused severe side effects, e.g., goblet cell metaplasia, immune suppression and skin cancer, and it is therefore of interest to explore alternative, non-GSI-based inhibitors for future clinical use. Other types of small molecule inhibitors have been developed, including RIN1^6^, CB-103^7^, NADI-351^8^ and Z271-0326^9^, which disrupt the Notch transcriptional complex, and CB-103 is currently investigated in phase 2 clinical trials. Compounds interfering with the Golgi apparatus or Notch posttranslational modification are also being explored^10,11^. Receptor-specific antibodies that block Notch receptor-ligand interaction or receptor processing represent an alternative approach which has shown promise, and antibodies that specifically block each of the four NOTCH receptors or Notch ligands are currently available^12–16^. These reagents have the advantage that they specifically target the activity of an individual receptor, but long-term use in preclinical animal models is associated with side effects^17^. There are to date no Notch inhibitors in routine clinical use^18,19^, except for the γ-secretase inhibitor (GSI) nirogacestat, which recently received FDA priority review for soft-tissue desmoid tumours with no FDA-approved treatments^20^.

In the light of the outlined limitations of current Notch-targeted approaches, new approaches are warranted to identify alternative Notch-directed therapies. In this report, we have taken an unbiased approach to identify novel Notch inhibitors based on a novel reporter assay recording signalling immediately downstream of the Notch receptor. We report the identification of five Notch inhibitor candidates with distinct chemical backbones, one of which constitutes a dihydroorotate dehydrogenase (DHODH) inhibitor. This identifies a role for DHODH in regulation of Notch signalling and our data suggest that DHODH inhibition may be an interesting avenue in the search for new ways to inhibit Notch signalling.

## Results

### A novel Notch reporter assay recording immediate downstream Notch signalling

To identify novel Notch inhibitors in an unbiased manner, we generated a novel Notch reporter construct, which records the level of Notch signalling immediately downstream of receptor activation rather than further downstream in the signalling cascade, for example at the level of Hes and Hey proteins (Fig. 1B). The reporter construct is based on a set of 12 CSL-binding sites linked to a firefly luciferase gene with a PEST domain attached C-terminally, to decrease reporter gene half-life and increase the temporal dynamics of the assay (Fig. 1B). The reporter construct carries piggy-bac sequences at both ends of the promoter-reporter cassette as well as cSH4 insulator elements to reduce transcriptional noise^21^ and was transfected into HEK293T cells. To achieve a robust level of Notch receptor activity in the HEK293T reporter cell line and to confine the search for inhibitors to compounds affecting the receptor function and eliminating the need for ligand activation, a truncated ligand-independent version of the Notch1 receptor, mimicking an S2-cleaved Notch1 receptor (Notch1ΔE) and spontaneously activated by S3-cleavage^22^ (Fig. 1C) was introduced into the cell line carrying the reporter construct (Fig. 1D). In addition, an HSV-TK-renilla luciferase plasmid was introduced into the cell line as an internal control for normalisation of the reporter expression (Fig. 1D).

A set of clones containing the reporter construct, the truncated Notch1 receptor and the renilla luciferase control were analysed for response to the GSI tert-Butyl (S)-{(2S)-2-[2-(3,5-difluorophenyl)acetamido]propanamido}phenylacetate) (DAPT), and the clone with strongest DAPT-mediated signal reduction was selected (clone A1-11; hereafter referred to as clone A) (Fig. 1E). Western blot analysis revealed that the majority of Notch1ΔE in clone A was spontaneously S3-processed, as expected, while processing was almost completely abolished when DAPT was supplemented to the cells (Fig. 1F). We next established the cell-based reporter system in a 384-well format, and both the firefly and renilla luciferase reporter activities were stable over time (> 1 h), produced Z-scores > 0.5 and the renilla signal was not affected by DAPT, as expected (Supplemental Fig. 1A; for evaluation of DSMO and DAPT effects see Supplemental Fig. 1B,C). Together, these data suggest that the novel reporter assay can dynamically read out Notch signalling and functions in a 384-well format to enable high throughput screening (HTS) of inhibitors.

### Screening of a small compound library for novel Notch inhibitors

To identify novel Notch inhibitors, we opted for screening a focused library consisting of 37,966 small molecule compounds, selected to represent broad structural diversity, while keeping a certain depth to allow crude structure–activity relationship studies^23^. The selection covered a large chemical space and was biased towards lead-like and drug-like profiles regarding molecular weight, hydrogen bond donors/acceptors and lipophilicity of compounds. The library furthermore included a nucleoside set from Berry & Associates as well as 1.200 compounds from the Prestwick collection of small compounds, which contains several EMA- and FDA-approved compounds and has specially been designed to increase potential high-quality hits (https://www.prestwickchemical.com/). As controls, we included a set of Notch inhibitors and GSIs. The small molecule Notch inhibitor FLI-O6^10^ and the GSIs LY-450139 (Semagacestat), LY-411575 and DAPT were tested in three doses (1, 5, 10 µM) resulting in a dose-dependent reduction of reporter signals (Supplemental Fig. 2A). LY-411575 and DAPT reduced the signal most efficiently at 10 µM (46% and 49%) and LY-450139 and FLI-06 reduced the signal to 52% and 62% of DMSO (Supplemental Fig. 2A), confirming DAPT as a reliable control to define the hit window; validation of normalization across plates and signal-to-background ratios, see Supplemental Fig. 1B–F).

**Fig. 2:**
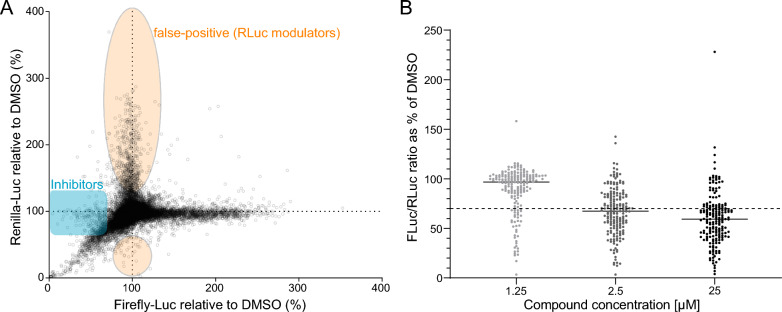
The 37,966 small compound library screen and compound confirmation assay. (**A**) A diagram showing the distribution of potential Notch inhibitors (decreased firefly luciferase activity; N = 189; blue sphere) and false-positive compounds (with an increase also in the renilla luciferase activity; N = 575; peach sphere). Firefly and renilla luciferase signals in % of DMSO are depicted on the x-axis and the y-axis, respectively. Screening was conducted after one day of compound exposure. The threshold for a Notch inhibitor was set to a decrease of firefly luciferase activity by more than 30% and no more than 25% increase in the activity of renilla luciferase. (**B**) Three dosage (1.25 10 and 25 µM) confirmation screen for the 189 inhibitors. The dotted line indicates the 70% reduction compared to DMSO cut off and 103 inhibitors were confirmed at 10 µM.

Screening of the 37,966 compounds identified 189 potential hits that matched the criteria: > 70% reduction compared to DMSO; < 25% difference from the Renilla-luc reporter (Fig. 2A, Supplemental Table 1). The 189 compounds were subsequently tested in a three-dose regimen (1.25, 10 and 25 µM), along with the established Notch inhibitors and three luciferase inhibitors (PTC-124, luciferase inhibitor 1 and Nano-Luc inhibitor) as controls. As expected, the luciferase inhibitors showed the strongest quenching of the signal: the signal was reduced by 83% and 80% by PTC-124 and luciferase inhibitor 1, respectively. No signal reduction was observed for the Nano-luciferase specific inhibitor (Nano-Luc), underscoring the specificity of the inhibitors (Supplemental Fig. 2G; for DMSO/DAPT signals and plate variations, see Supplemental Fig. 2H–L). In the three-dose assay, 103 of the 189 compounds showed inhibition of reporter activity at 10 µM (which was defined as the cut-off), while 37 and 136 compounds showed reduced reporter activity at 1.25 and 25 µM, respectively (Fig. 2B; Supplemental Table 1). In sum, after the initial screening of 37,966 compounds, 103 compounds matching the preset hit criteria remained.

### Removal of potential γ-secretase inhibitors through an APP-based counter screen

**Fig. 3:**
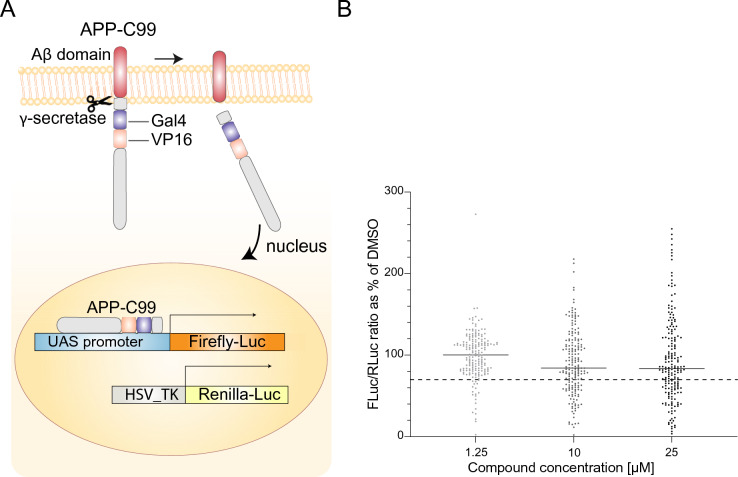
Analysis of Notch inhibitor candidates by the APP-C99 counter screen to eliminate potential GSIs. (**A**) Schematic depiction of the APP-C99 counter assay^24^. The engineered APP protein contains a GAL4/VP16 activation domain, which activates a UAS-firefly luciferase reporter gene construct upon cleavage of APP by the γ-secretase complex. The HSV-TK promoter-renilla luciferase construct was used as a control for luciferase activity. (**B**) The 103 potential inhibitor hits from Fig. 2B were passed through the APP-C99 counter screen at three dosages (1.25, 10 and 25 µM). The dotted line indicates the 70% reduction compared to DMSO cutoff and 53 potential inhibitor hits passed the APP-C99 counter screen at 10 µM.

As the initial set of hits might include potential GSIs, we performed a counter screen to remove such compounds, because GSIs are already well-established as pre-clinical pan-Notch inhibitors and have shown side effects in clinical trials^5,18^. To this end, we used a previously established reporter gene-based GSI assay (APP-C99) (Fig. 3A), which measures γ-secretase activity through cleavage of amyloid precursor protein (APP), another protein processed by the γ-secretase complex^24^ (for analysis of assay stability and variation, effects of established Notch and luciferase inhibitors, see Supplemental Fig. 3A,B). Using 70% signal reduction compared to baseline (DMSO) at 10 µM as threshold for removing a compound, 50 of the 103 compounds were removed as potential GSIs (Fig. 3B, Supplemental Fig. 3C, Supplemental Table 2).

The remaining 53 compounds were then filtered for undesired chemical properties, such as pan-assay interference compounds (PAINs), Rapid Elimination Of Swill (REOS, for review see^25^) and compounds that generated false positive results and may act on luciferase directly were also removed. Together, 11 compounds were eliminated from further analysis, and the 42 remaining compounds were then subjected to an extended 11-point dose–response regimen (39 nM-40 µM) in the APP-C99 counter screen, leading to removal of an additional three compounds. Thus, 39 potential inhibitor hits remained for subsequent analysis (Supplemental Table 3; for inhibitor controls and dose response curves see Supplemental Fig. 3D,E and for plate statistics see Supplemental Fig. 3F,G).

### Identification of five inhibitor hits

To further narrow down the number of compounds, the 39 inhibitor hits passing the APP-C99 counter assay were scrutinized for various properties. Sixteen compounds predicted to have favourable pharmacogenetic, pharmacodynamic and toxicology properties based on their chemical structures were selected for subsequent experiments. These compounds were grouped based on their diverse core chemistries, and analogues from existing libraries were identified, resulting in five lead series. The selected analogues added 47 structurally related compounds to the 16 hits (for full list and chemical structures of the 16 compounds and respective analogues see Supplemental Table 4A). All 63 compounds were analysed in the APP-C99 counter assay in an optimized 14-point dose–response analysis format, which led to removal of 11 compounds (one original compound and 10 analogues) that decreased the signal less than 70% compared to DMSO controls, leaving 52 compounds for subsequent analyses (for assay optimisation see Supplemental Fig. 4A; for plate statistics and inhibitor controls see Supplemental Fig. 4B, dose response curves are shown in Supplemental Fig. 4C and IC50s and Hill-slope values are presented in Supplemental Table 4B).

Next, we subjected the 52 compounds to an optimized 14-point dose–response Notch reporter assay analysis, to establish inhibitory concentration 50 (IC50) values (for optimization of the assay using previously identified GSIs see Supplemental Fig. 4D; for technical details of the analysis see Supplemental Fig. 4E,F; a summary of IC50 and area under curve (AUC) values for known Notch inhibitors in 9-point and 14-point Notch dose optimisation assays is presented in Supplemental Table 4C; and IC50 correlation graphs are provided in Supplemental Fig. 4G). Based on a 11-point pre-screen (data not shown), the 52 compounds were sorted into three baskets, each corresponding to an adjusted dose-range aiming to improve the curve fits and IC50s for the individual compounds in each basket. Of the 52 compounds, 22 showed an apparent signal reduction (14 of the original compounds and eight analogues (see Supplemental Table 4D for IC50 and Hill-slopes values).

**Fig. 4:**
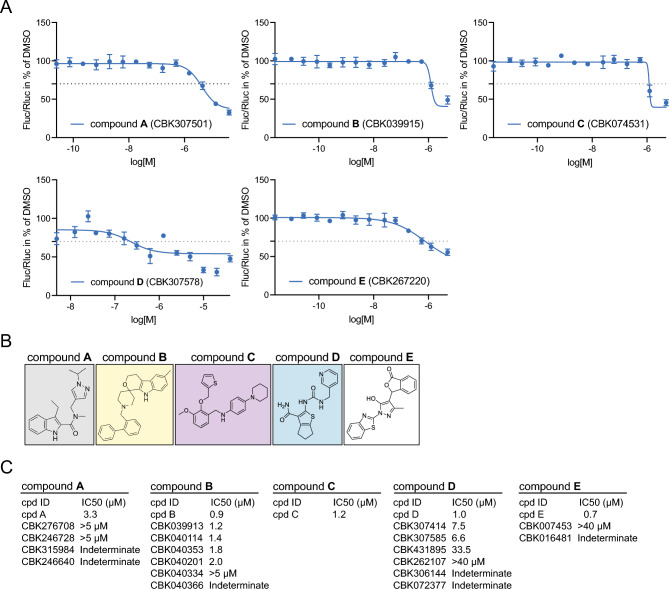
The five inhibitor hits A-E. (**A**) Dose–response curves from the Notch reporter assay using compound A-E at 14 different concentrations. (**B**) The chemical structures of compound A-E. (**C**) The approximated IC50 values for the A-E compounds and names and IC50 values for analogues to the respective compound. Indeterminate indicates that an IC50 could not be established.

Five of the original 16 hit compounds, hereafter referred to as compounds A-E, exhibited approximated IC50 values (as IC50 values could not be calculated precisely) between 0.73 µM (compound E) and 4 mM (compound A) and Hill slopes between -16.2 (compound C) and -1.7 (compound D) (Fig. 4A, Supplemental Fig. 4H). The structures of compounds A-E are shown in Fig. 4B. Three analogues to compound B showed an IC50 < 2 µM while other analogues showed IC50s > 4 µM or did not result in proper curve fits (Fig. 4C, see Supplemental Fig. 4I and Supplemental Table 4E for area under curve analysis and Supplemental Table 4F for a comparison of IC50 values of hits and Notch inhibitors in both assays). Analysis of compounds A-E in the APP assay showed that only compound A showed an inhibitory effect at high concentrations; a direct comparison of the hits dose–response curves is presented in Supplemental Fig. 4J. In keeping with the lack of activity in the APP assay, none of the five compounds blocked cleavage of the Notch intracellular domain (Supplemental Fig. 4K; for quantification of western blot signals see Supplemental Table 4G). In sum, compounds A-E were selected for further analysis based on their structural properties and IC50 values.

### Assessing Notch inhibitor hits A-E in an orthogonal assay based on myogenic differentiation

**Fig. 5:**
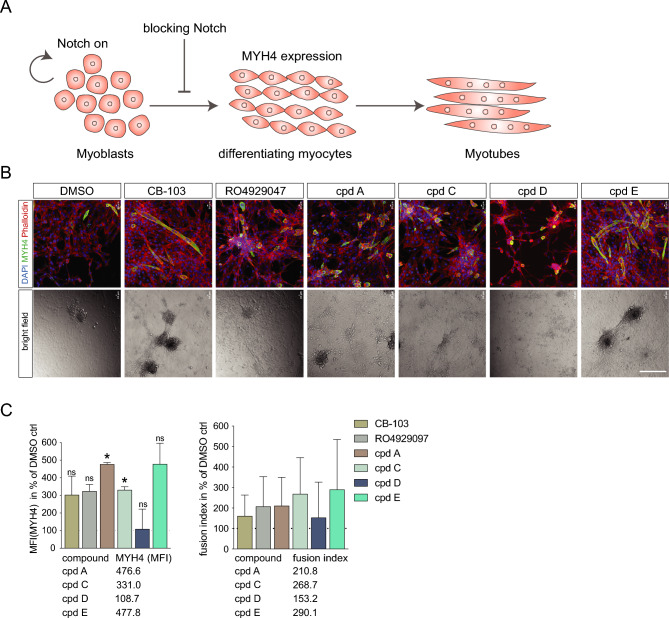
Analysis of compounds A-E in an orthogonal screen based on myogenic differentiation of C2C12 cells. (**A**) Schematic representation of the C2C12 myogenic assay system and how Notch blocks myogenic differentiation. (**B**) Immunocytochemistry (ICC) analysis of C2C12 cells five days after treatment with 10 µM of compound A, C, D and E, using DMSO as a reference and CB-103 and RO4929047 as controls (MYH4 antibody staining in green, Phalloidin in red, DAPI in blue). Below, bright field microscopy images of cells treated with compound A-E are shown. Scale bar: 400 µm. (**C**) Median fluorescence intensity of MYH4 (a myocyte marker) ICC staining in the C2C12 cells five days after treatment with 10 µM of compound A, C, D and E, and with DMSO as a reference. Treatment with CB-103 and ROI4929047 was included as controls. (**D**) Fusion of nuclei (as an indicator of differentiation) in the C2C12 cells five days after treatment with 10 µM of compound A, C, D and E, with DMSO for cells grown in differentiation medium as a reference, and CB-103 and RO4929047 as controls. Two-tailed student’s *t*-tests were performed (Welch’s test) against the reference with *p ≤ 0.05, ns = not significant.

To corroborate their status as Notch inhibitors, we tested four of the five compounds (A, C, D, E) in an orthogonal assay for Notch function. Notch signalling plays an important role in myogenic differentiation^26^, and blocking Notch signalling promotes myogenic differentiation in the myoblast cell line C2C12^27^ (Fig. 5A). C2C12 cells can be maintained as myoblasts but upon serum starvation spontaneously differentiate to MYH4-positive multinucleated myotubes, a process which is accelerated by Notch blockade^27^. When subjected to the four compounds or to RO4929097 as GSI control for five days, C2C12 myoblasts showed elevated expression of the differentiation marker MYH4 for three of the candidate compounds (A, C, E) as well as for RO492097, accompanied by a transition towards a polynucleated myotube morphology (Fig. 5B). Treatment with compound D did not produce elevated MYH4 levels but resulted in cell shape changes, with a transition towards a spindle-like morphology, indicating an onset of differentiation. Compound E, as well as the positive controls RO492097 and CB-103^7^, increased MYH4 levels, although not reaching statistical significance (Fig. 5B,C; for calculation of mean fluorescence intensity (MFI) and fusion index see Supplemental Table 5). Together, these data suggest that compounds A, C and E are endowed with Notch-inhibitory activity in the myogenic differentiation assay.

### Analysis of growth-inhibitory effects in tumour cell lines

**Fig. 6:**
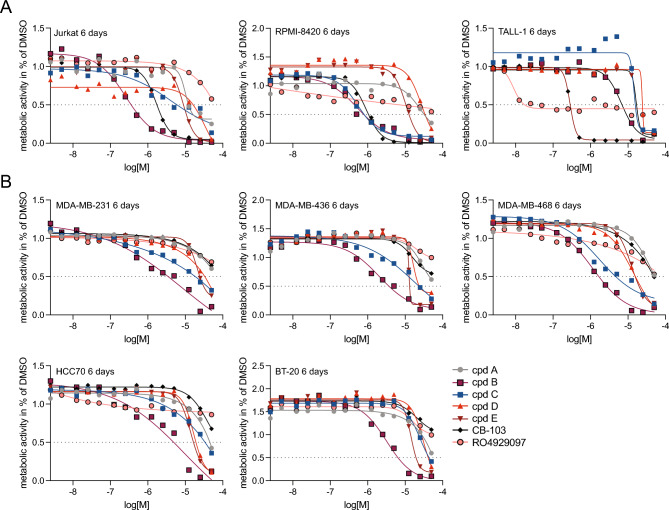
Analysis of compounds A-E for growth-inhibitory effects on T-ALL and breast cancer cell lines. (**A**) Graphs showing 14-point growth-response curves for the level of metabolic activity in three T-ALL cell lines (JURKAT, RPMI-8402 and TALL-1) at six days after treatment with compound A-E, and CB-103 and RO4929097 as controls. (**B**) Graphs showing 14-point growth-response curves for the level of metabolic activity in five breast cancer cell lines at 6 days after treatment with compound A-E, and CB-103 and RO4929097 as controls. Results of two biological assays were averaged for potential hit compounds.

We next investigated whether compounds A-E affected growth and viability of cell lines from T-cell acute lymphoblastic leukaemia (T-ALL) and breast cancer, two tumour types in which elevated Notch signalling has been implicated in tumorigenesis^3^. We analysed the metabolic activity (CellTiter-Glo, Promega) in three T-ALL and six breast cancer cell lines exposed to 14 different concentrations (50 µM to 2.5 nM) of compounds A-E. Growth of the NOTCH1-addicted T-ALL cell lines JURKAT and RPMI-8402 was significantly reduced upon compound B or C treatment, resulting in approximated IC50s of 0.3 µM and 4.3 µM in JURKAT cells and 0.5 µM and 0.6 µM in RPMI8402 cells after six days, for compound B and C, respectively (Fig. 6A). Furthermore, the NOTCH3-addicted TALL-1 cell line showed growth inhibition after treatment with compound B for six days with an approximated IC50 of 6.7 µM. As control, the Notch inhibitor CB-103 reduced growth with similar potency as compound B and C in JURKAT and RPMI-8402 cells (approximated IC50 1.7 µM and 0.9 µM after six days) and reduced growth of TALL-1 cells most efficiently after 6 days (approximated IC50 0.3 µM). When testing analogues of compounds B and C, five of the seven B analogues and one of the two C analogues produced dose–response curves and IC50s, which however were higher compared to the original hits (Supplemental Fig. 5A; IC50s are presented in Supplemental Table 6).

Growth of the breast cancer cell lines MDA-MB-231 and HCC70 was only marginally affected by compound A-E treatment after three days (IC50s > 25 µM) (Supplemental Fig. 5B, Supplemental Table 6), but after six days of treatment with compound B, there was a robust decrease in metabolic activity in the MDA-MB-436, MDA-MB-468 and BT-20 cells (approximated IC50s of 2 µM, 1.1 µM, and 3.2 µM, respectively). Treatment with compound C for six days resulted in an approximated IC50 of 1.7 µM in MDA-MB-468 cells, but higher IC50s (> 10 µM) in the other breast cancer cell lines (Fig. 6B). Compound A, D and E treatment resulted in IC50 values > 10 µM for all cell lines. We also tested three analogues to compound B and one analogue to compound C. The compound C analogue produced a dose–response curve fit with IC50 > 5 µM in MDA-MB-468 cells and > 10 µM in MDA-MB-231 cells, whereas the compound C analogue produced IC50s > 10 µM in MDA-MB-468 and MDA-MB-231 cells (Supplemental Fig. 5C; for IC50s and AUC values see Supplemental Table 6 and a heatmap for IC50 and AUC values is presented in Supplemental Fig. 5D). To assess the effects of compound A-E in a cell line with very low levels of Notch signalling we repeated the growth inhibition assay in HEK293T cells. Compound A, D and E did not exert a growth inhibitory effect, while compound B and C did (Supplemental Fig. 5E).

Next, we were interested in learning whether compounds A-E affect expression of Notch downstream genes. Analysis of Notch downstream gene (HES1, c-MYC, JAGGED1 and HES4) expression in the tumour cell lines revealed a dose-dependent downregulation of HES1 and c-MYC expression in T-ALL cells upon treatment with compound B, C, D and E (Supplemental Fig. 6A). HES1 was downregulated in MDA-MB-231 cells when treated with compound A, D and E, and expression of HES4 was reduced after treatment with A, B, and D, but upregulated when treated with compound C, while the transcript levels of the Notch ligand Jagged1 were reduced when cells were treated with compound A, B, D and E in MDA-MB-231 cells. HES4 expression was downregulated in MDA-MB-468 cells when treated with compound B-E. CB-103 treatment reduced c-MYC expression in T-ALL cell lines but unexpectedly led to a trend towards upregulation of c-MYC in the MDA-MB-231 cell line and upregulation of HES4 in MDA-MB-468 cells (Supplemental Fig. 6B,C). In contrast, the GSI RO4929097 consistently reduced HES1 and HES4 target gene expression across the panel of cell lines, and c-MYC in T-ALL cells, but did not reduce c-MYC levels in MDA-MB-231 and MDA-MB-468 cells (Supplemental Fig. 6B,C). In sum, compounds B and C induced growth inhibition and downregulation of Notch target genes in several breast cancer and T-ALL cell lines.

### Compound C is a dihydroorotate dehydrogenase inhibitor

**Fig. 7:**
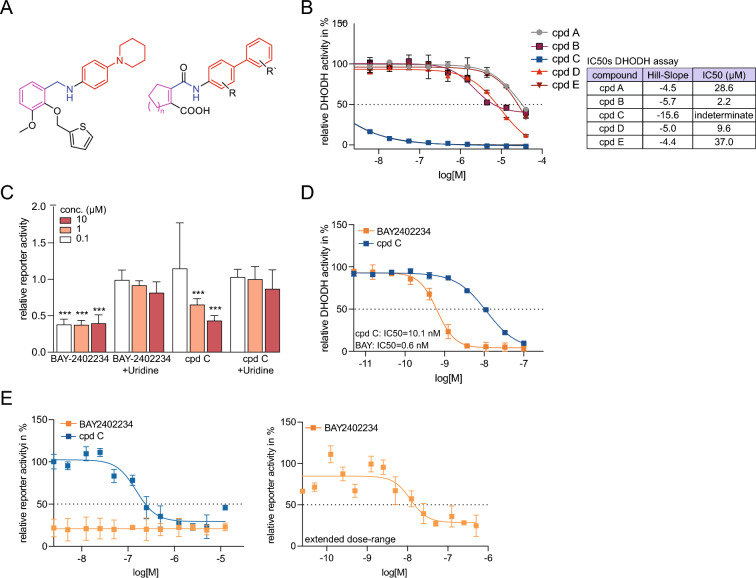
Compound C is a DHODH inhibitor. (**A**) Chemical structures of compound C and a previously proposed potent DHODH inhibitor^28^. The colours indicate similarities between the compounds, with two hexacyclic ring arrangements (red) connected through an amine-like linker (blue) to a second ring arrangement (magenta). (**B**) Relative DHODH activity for compound A-E. IC50 levels are presented to the right. (**C**) The levels of Notch reporter activity after 24 h treatment with DHODH inhibitor BAY2402234 and compound C at 0.1, 1 and 10 µM in presence or absence of 100 µM uridine. Bars represent reporter activity relative to DMSO as control for the BAY2402234 and the cpd C columns and with DMSO + 100 µM uridine as control for the BAY2402234 + uridine and the cpd C + uridine columns. Bars are presented as mean and error bars display SD of three biological assays. Multiple unpaired t-test with Welch’s correction was performed comparing each treatment with its corresponding control as reference. Changes were considered significant with *p < 0.05, **p < 0.01, ***p < 0.001. (**D**) Relative DHODH activity for compound C and BAY2402234. IC50 values are presented in the bottom left of the graph. (**E**) (Left) Activity of BAY2402234 and compound C in the Notch reporter assay at a dose range from 50 µM-2.5 nM, and with an IC50 for compound C of 150 nM. (Right) Activity of BA2402234 in the Notch reporter assay at a dose range from 500 nM-25 pM, and with an IC50 for BAY2402234 at 11 nM.

Compound C had an overall attractive profile, accelerated myogenic differentiation and potently inhibited tumour cell line growth, and we therefore decided to explore it in more detail. When comparing its structure to that of other known classes of inhibitors, we noted that compound C was partially structurally related to an earlier-generation inhibitor of dihydroorotate dehydrogenase (DHODH)^28,29^ (Fig. 7A). DHODH is an enzyme localized to the inner mitochondrial membrane, where it converts dihydroorotate to orotate in the de novo pyrimidine synthesis pathway^30^. The fact that a genetic link between Notch and DHODH had previously been published^31^ further spurred us to test whether compound C was endowed with DHODH-inhibiting properties. Testing of compounds A-E in an assay for DHODH enzyme activity demonstrated that compound C appeared to reduce DHODH enzyme activity across the dose response range, while none of the other four compounds exhibited DHODH enzyme-blocking activity (Fig. 7B, Supplemental Table 7A). This observation was corroborated by the finding that compound C reduced the reporter assay activity at 1 and 10 µM concentrations, and this effect could be reversed by addition of 100 µM uridine (Fig. 7C, Supplemental Fig. 7A), which is known to abrogate the effect of DHODH inhibitors^32^. When compound C was compared to the well-established DHODH inhibitor BAY2402234 at a broader dose range, we observed that BAY2402234 was approximately 15-fold more potent than compound C as a DHODH inhibitor (Fig. 7D). Spurred by BAY2402234’s higher DHODH-inhibiting activity, we finally assessed whether BAY2402234 would also be a potent Notch inhibitor. At an expanded dose range (500 nM-25 pM, Fig. 7E), BAY2402234 blocked Notch reporter activity with an IC50 of 11 nM, compared to 150 nM for compound C (Fig. 7E). Reassessing the hit compounds in the same expanded dose range as compound C (Fig. 7E; 50 µM-2.5 nM) led to improved approximated IC50 values (Supplemental Fig. 7B; for IC50s and Hill-slope values see table Fig. 7E and for AUC values see Supplemental Table 7B).

### Compound C reduces the level of the Notch downstream protein HES-1

**Fig. 8:**
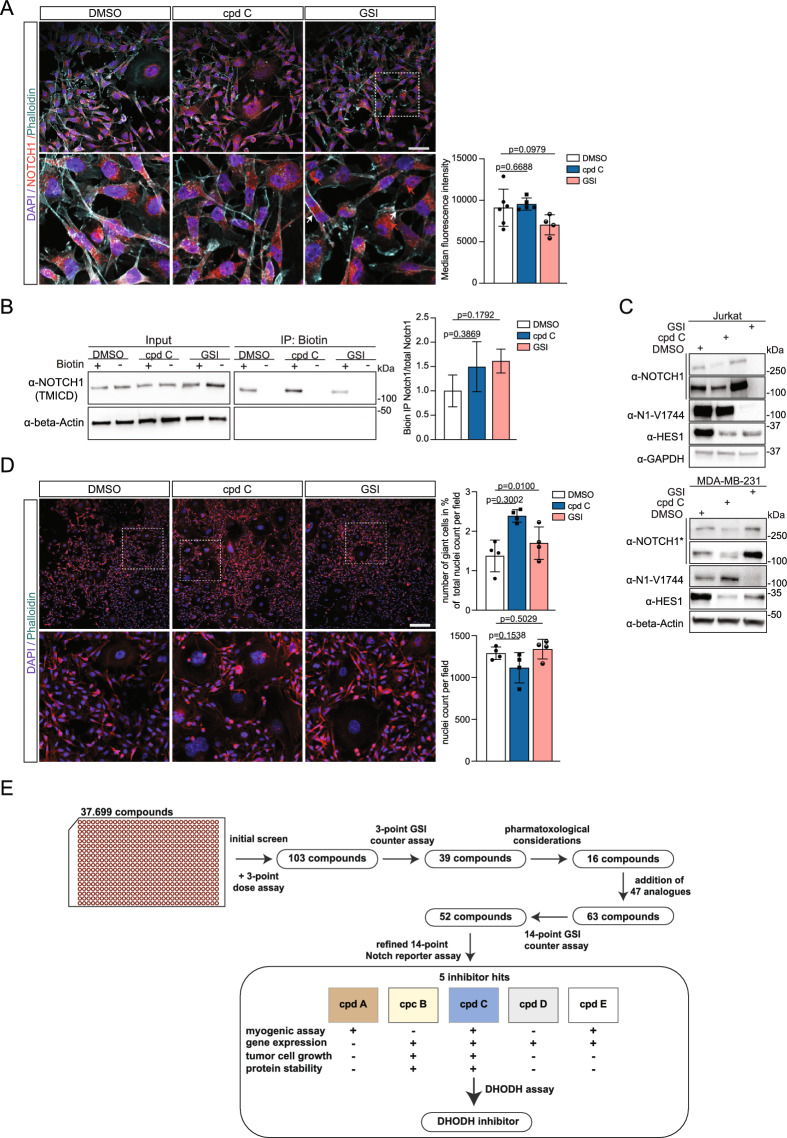
Assessment of effects of compound C on Notch signalling. (**A**) Immunocytochemistry (ICC) analysis of NOTCH1, phalloidin and DAPI staining in MDA-MD-231 cells cultured for 24 h in the presence of 2 µM compound C (cpd C), 2 µM GSI (RO4929097) or DMSO, as control. White arrows indicate signal overlap between NOTCH1 and phalloidin and red arrows point towards NOTCH1 signals. To the right, quantification of NOTCH1 ICC based on data from six (DMSO), five (cpd C) and four images (GSI). Two-tailed student’s *t*-tests were performed (Welch’s test) with DMSO as reference. The dotted area in the upper panel is enlarged in the panel below. Scale bar: 50 µm. (**B**) Western Blot analysis of surface biotinylation experiment of MDA-MB-231 cells after 24 h treatment with 2 µM compound C, GSI (RO4929097), or DMSO as control. Cells were either subjected to biotinylation or left untreated as controls (input, left panel), followed by pull-down of biotinylated proteins (right panel). Results were visualised with anti-NOTCH1 antibody, and beta-actin antibody as loading control. Input samples were run on one gel and the corresponding immunoprecipitation (IP) samples on a second gel. After transfer, the membrane was cut, and the upper part was probed with anti-NOTCH1 and the lower part with anti-beta-actin antibody. To the right, quantification of biotin-immunoprecipitated NOTCH1 based on data from two independent experiments. Two-tailed student’s *t*-tests were performed (Welch’s test) with DMSO as reference. (**C**) Western blot analysis of NOTCH1 and HES1 protein levels in Jurkat (upper) and MDA-MB-231 (lower) cells in response to 48 h treatment with DMSO, 2 µM compound C and GSI (RO4929097). Beta-actin or GAPDH staining were included as loading controls. The a-NOTCH1 antibody recognizes both the full length, TMIC and ICD forms of NOTCH1, with the full-length form at approximately 250 kDa and processed forms at around 100 kDa. Activated NOTCH1 was visualized using an antibody targeting the S3 cleaved form of NOTCH1 (a-N1-V1744). The MDA-MB-231 samples were run on the same gel, and the membrane was cut after transfer. The lower part was incubated with anti-beta-actin antibody and the upper part with anti-NOTCH1 antibody. The upper part depicts a longer exposure time of full-length NOTCH1. The asterisk denotes that the Western blot membrane was stripped and reprobed with the a-NOTCH1 antibody a-N1-V1744. After development of beta-Actin signals, the membrane was cut and probed with anti-HES1 antibody. Jurkat cell samples were separated on the same gel. The upper part was developed with an a-N1-V1744 and the lower part with a-HES-1 antibody. The blot was stripped and the lower part was reprobed with a-GAPDH and the upper part was reprobed with a-NOTCH1 antibody. (D) Immunocytochemistry of NOTCH1 in MDA-MB-231 after 24 h of treatment with 2 µM of indicated compounds. F-actin was visualised with phalloidin (magenta) and nuclei were stained with DAPI. The quantification of total nuclei counts of four fields per condition and the number of giant cells in percent of nuclei count per field is shown in the graphs to the right count (top, respectively bottom). Two-tailed student’s *t*-tests were performed (Welch’s test) with DMSO as reference. (**D**) Immunocytochemistry (ICC) analysis of NOTCH1, phalloidin and DAPI staining in MDA-MD-231 cells cultured for 24 h in the presence of 2 µM compound C (cpd C), RO4929097 (GSI) or DMSO, as control. To the right, quantification of the number of giant cells in % of total nuclei count per field (upper) and the nuclei count per field (lower) are shown, based on four different fields per condition. Two-tailed student’s *t*-tests were performed (Welch’s test) with DMSO as reference. The dotted area in the upper panel is enlarged in the panel below. Scale bar: 200 µm. (**E**) A summary of the small compound screening strategy leading to the identification of compound C as a novel DHODH inhibitor. The various steps in the compound screening procedure are depicted. The numbers in the boxes illustrate the number of candidate hits after each screening step. In the bottom panel, the performance of compound A-E in the various deconvolution steps is shown (+ indicates that the compound had an effect; − indicates that there was no significant effect in the respective assay). When assaying for DHODH inhibitor activity, compound C turned out to be a novel DHODH inhibitor.

We next explored how compound C inhibited Notch signalling in the MDA-MB-231 cells, a cell line chosen because it has a robust endogenous level of Notch signalling. The overall level of NOTCH1 was not affected by compound C in MDA-MB-231 cells, while there was a trend towards reduced levels after GSI treatment (Fig. 8A, the corresponding MFI values are presented in Supplemental Table 8A). Cell surface biotinylation experiments revealed that NOTCH1 surface presentation was not hindered in compound C-treated cells; the amount of NOTCH1 at the cell surface was approximately the same as in control cells or cells treated with GSI (Fig. 8B). To determine whether the reduced levels of Notch1 ICD resulted in lowered levels of Notch downstream signalling, we assessed the amount of HES1 protein in MDA-MB-231 cells, and treatment with 2 µM compound C for 48 h caused a reduction in HES1 levels (Fig. 8C). Similarly, the level of HES-1 protein was reduced by compound C in JURKAT cells (Fig. 8C). In MDA-MB-231 cells, we also observed an increased fraction of cells with large nuclei following compound C treatment for 24 h compared to DMSO control, and a smaller increase was observed following GSI exposure (Fig. 8D). Data from a 72-h treatment point and the corresponding nuclei and giant cell counts are presented in Supplemental Fig. 8A and Supplemental Table 8B, respectively.

We also asked whether the stability of the cleaved form of the receptor was affected by compound C. Cycloheximide experiment to measure the half-life of NOTCH1 showed that the cleaved form of the receptor is more stable in DMSO controls compared to compound C treated cells (t_½_ 5.6 h and 4.5 h, respectively) (Supplemental Fig. 8B, see Supplemental Table 8C for densitometric measurements). Treatment with a GSI resulted in enhanced protein stability (Supplemental Fig. 8B), which is in line with the expected accumulation of unprocessed protein within the cells. To corroborate the notion that compound C may reduce Notch1 levels, we assessed the amount of Notch1 ICD in an alternative manner, using a mouse Notch1ΔE (mNotch1DE) moiety^22^, which spontaneously generates mNotch1 ICD. After engineering a stable cell HEK293T cell line with mNotch1ΔE fused to an HiBiT tag (Supplemental Fig. 8C), exposure to compound C in a 14-point dose–response assay resulted in a dose-dependent decrease of luciferase signal (Supplemental Fig. 8D, Supplemental Table 8D); for signal stability and variation see Supplemental Fig. 8E; for corroborating data from a second clonal cell, see Supplemental Fig. 8F). As controls, treatment with Bafilomycin A1, which prevents protein degradation by inhibiting autophagosome-lysosome fusion^33^, or the proteasome inhibitor MG132 increased the HiBiT signal (Supplemental Fig. 8G). In conclusion, these data suggest that compound C does not hinder surface presentation or processing of the NOTCH1 receptor but may reduce protein half-life and HES-1 protein levels.

## Discussion

Hyperactivated Notch signalling is linked to several types of disease and cancer, and there is thus a need to develop novel Notch inhibitors^18,19^. In this report, we conducted an unbiased large-scale small molecule screen to identify potential Notch inhibitors based on a novel reporter assay for immediate downstream Notch signalling as the read out. An initial screening of 37,966 compounds was followed by a counter screen for GSI activity and an orthogonal screen for blockade of myogenic differentiation, which eventually led to the identification of five hits (compounds A-E); the stepwise screening is schematically depicted in Fig. 8E. The five hits have distinct chemical structures, indicating that they may target Notch signalling by distinct mechanisms and that the screen indeed can capture different types of Notch inhibitor candidates.

Among the five inhibitor hits, we focused our attention on compound C, which showed structural similarities to dihydroorotate dehydrogenase (DHODH) inhibitors. Compound C was confirmed as a DHODH inhibitor in a DHODH enzymatic assay, and uridine treatment furthermore abrogated the inhibition of Notch signalling by compound C in the Notch reporter assay, in keeping with DHODH’s critical role for pyrimidine production^30^. The well-established DHODH inhibitor BAY2402234 turned out to inhibit DHODH activity with higher efficacy than compound C, and when BAY2202234 was analysed in the Notch reporter assay, it inhibited reporter activity with an IC50 of 11 nM, making it a highly potent Notch inhibitor. Our findings of a link between DHODH and Notch signalling are consistent with several earlier studies. In the fruit fly, *Drosophila melanogaster*, chemical DHODH inhibition by 5-methyl orotate phenocopied *Notch* mutations, including notches of wings and bristle multiplication^31^. Furthermore, it was recently shown that DHODH inhibition, via administration of the DHODH inhibitor brequinar, was curative in a Notch1 ICD-induced mouse model of T-ALL^34^. Finally, a recent report demonstrated that increased expression of DHODH may lead to elevated levels of Notch1 ICD^35^. Together, these studies substantiate a link between DHODH function and Notch signalling and supports the notion that DHODH inhibition may be an interesting avenue to explore for novel Notch inhibitors.

The mechanistic coupling between DHODH function and Notch signalling largely remains to be elucidated, but our data from deconvolution studies of compound C reveal a reduction in the amount of NOTCH1 ICD in MDA-MB-231 cells, which may indicate a decreased stability and more rapid turnover of NOTCH1 ICD. Alternatively, compound C may cause a shift in localization, although the distribution of NOTCH1 at the cell surface was not significantly altered. As the DHODH enzyme is primarily localized to the inner mitochondrial membrane it is unlikely that it directly affects Notch receptor stability. The reduction of NOTCH1 ICD levels may therefore be an indirect effect of DHODH acting via proteins that posttranslationally modify Notch ICD and reduce Notch ICD stability, for example kinases such as PIM kinases^36,37^ and E3 ubiquitin ligases such as cdc4^38^. Hydroxylases or sumoylases may also be involved as intermediates^39^. Another possibility is that the effect on Notch instead is mediated by downstream effects of DHODH inhibitors. For example, it has been demonstrated that DHODH inhibitors block the degradation of p53^32^, and there is evidence for a functional relationship between Notch signalling and p53^40,41^. Furthermore, DHODH inhibitors may regulate MYC^42^, which also is linked to Notch signalling^43,44^, and a downregulation of c-MYC is observed after compound C treatment in MDA-MB-231 cells. Recently, it has also been shown that DHODH inhibitors induce specific metabolomic changes in lung cancer cell lines^45^, but whether this affects Notch signalling remains to be investigated. Alternatively, as addition of uridine reversed the effect of compound C, it is also possible that the role of DHODH in pyrimidine nucleotide synthesis is somehow related to the effect of compound C on Notch signalling. Additional research will be required to decipher the precise mechanistic link between Notch and DHODH.

The finding that DHODH inhibitors reduce Notch signalling is also of importance for a better understanding of potential side effects emerging from off-target Notch inhibition when DHODH inhibitors are used in specific disease settings. DHODH inhibitors, such as leflunomide and teriflunomide, are currently used in therapy for rheumatoid arthritis, multiple sclerosis, psoriasis and diabetes^30,46,47^ and the range of diseases in which DHODH inhibition may be beneficial is increasing: there is an emerging interest in DHODH inhibition in malaria treatment^48,49^ and for acute myeloblastic leukaemia therapy, where DHODH inhibition may enhance myeloid differentiation^50^. In these situations, it is important to learn about potential consequences of the DHODH inhibitors simultaneously reducing Notch signalling; adverse effects of off-target reduction of Notch signalling have been painfully learned from the use of GSIs in clinical trials for Alzheimer’s disease. As GSIs not only block APP processing but also abrogate Notch receptor cleavage, patients in such trials showed reduced Notch signalling, accompanied with for example gastrointestinal toxicity, immune suppression and increased risk for skin cancer^18^. In comparison with other recent Notch inhibitors, compound C performs somewhat differently in the various assays (Supplemental Fig. 9). Notably, the GSI LY-411575 showed the highest activity in the Notch reporter assay, while CB-103 showed a lower activity than compound C. In the myogenic assay, compound C also showed a stronger inhibitory effect as compared with CB-103. Unexpectedly, c-MYC levels were enhanced by CB-103 and the GSI RO4929097. The different performances may at least in part be attributed to that the various inhibitors have been identified through different assays and therefore may interfere with Notch signalling in different ways: Compound C was identified through a Notch downstream reporter assay while NADI-351 and Z271-0236 were identified through assays based on disruption of the Notch transcriptional complex in various ways^8,9^ and CB-103 was identified by a transient transfection reporter assay^7^.

In conclusion, we show that an unbiased small compound screen monitoring the level of Notch downstream activation yielded distinct types of inhibitor hits and importantly unravelled a link between DHODH and Notch signalling. This will lay the foundation for further exploration of novel Notch inhibiting strategies based on DHODH inhibition and may also inform on potential Notch off-target effects from the current use of DHODH inhibitors in the clinic.

### Limitations of the study

One limitation of this study is that we have not yet fully elucidated the mode of action for compound C and how Notch and DHODH interact at a deeper molecular level. While our data suggest that the amount of NOTCH1 ICD is reduced, other modes of action are plausible, including an involvement of phosphoglycerate kinase 1 (PGK1), based on the notion that compound C was also identified in a screen for PGK1 inhibition and there are indirect links between Notch and PGK1 via PI3K signalling. Another limitation is that compound C showed a growth inhibitory effect also in HEK293T cells, a cell line with very low levels of Notch signalling. We however believe that this is attributed by its function as a DHODH inhibitor, as the well-established DHODH inhibitor BAY2202234 exerted a similar growth inhibitory effect (Supplementary Fig. 5E), likely a result of pyrimidine depletion after six days of culturing. In line with this reasoning, the observation that compound C and BAY2402234 showed strong effects in HEK293T cells but not in some other cell lines may be explained by different sensitivity to pyrimidine deprivation among the cell lines. This suggests that it may be difficult to strictly assess compound C’s growth inhibitory effects in cell lines, and that alternative assays need to be deployed prior to moving to evaluating its efficacy as a Notch inhibitor in experimental animal models. Finally, as the Notch and APP screens are based on the same promoter, they may eliminate compounds acting on downstream targets that are shared between Notch and APP, such as ER exportin inhibitors like FLI-06.

## Materials and methods

### Generation of the Notch reporter construct and the reporter cell line

A de novo Notch reporter construct, composed of six dimeric CSL binding sites linked to a basic b-globin promoter and followed by a firefly luciferase gene and a PEST degradation domain was synthesised (GeneArt). The construct was stably introduced into HEK293T cells, and zeocin (0.1 mg/ml) or Puromycin (0.05 mg/ml (ThermoFisher, cat #R25001, #A1113803) were used as selection markers. A clonal cell line (clone #A1) with robust renilla expression and enhanced firefly luciferase expression upon transient transfection of Notch1 ICD or Notch1ΔE constructs was selected, and a UB6 promoter-Notch1DE construct was stably introduced into this cell line using Blasticidin (0.1 mg/ml; ThermoFisher, cat #A1113903) as a selection marker. Stable cell lines were generated by serial dilution and only clonal colonies were selected for further evaluation. To measure luciferase reporter activity, cells were washed in cold PBS, detached, and collected in pre-warmed cell culture medium and centrifugated for five min at 200xg. The cell pellet was resuspended in medium, cells were counted, and 4,000 cells were seeded in each well of a white 384 well plate (Corning #3670) in 20 µl medium containing either DMSO or the GSI DAPT (N-[N-(3,5-Difluorophenacetyl)-L-alanyl]-S-phenylglycine t-butyl ester, Sigma-Aldrich, cat #565784-M) and incubated for 24 h. The efficacy of the reporter gene system was determined by monitoring the level of luciferase activation in the absence or presence of different amounts of DAPT in several cell clones. Luciferase activity was assessed using the dual-GLO luciferase detection kit from Promega (cat # E2920), following the manufacturer’s instructions. A minimum of two technical replicates were measured per clone using a SpectraMaxi3 plate reader (Molecular Devices). Reporter activity of different clones and in response to DAPT was assessed by normalising firefly (Notch activity) to renilla luciferase signals. Normalised signals of individual clones cultivated in DMSO were then compared to corresponding signals generated with DAPT-treated cells. The cell clone with the strongest signal to control ratio (DMSO to DAPT) was then selected for further exploration (clone #A1-11).

### Generation of the HiBit reporter construct and cell line

The Notch1ΔE construct used to generate the Notch reporter cell line (see above) was first cloned in frame into the pBiT3.2-C [TK/HiBiT/Blast] Vector (Promega) and then stably introduced into HEK293T wild type cells, using Lipofectamine transfection, as described. Analogous to the Notch reporter cell line, Blasticidin was used as a selection marker (0.1 mg/ml) and stable cell lines with robust luciferase signals were generated. HiBit signals correlated with cell numbers and clone #A4F2 showed the overall best luciferase signal to control and signal-to-noise ratio and was selected for further analysis. A clone (#D2) with an empty HiBit vector stably integrated was used as control.

### RNA isolation and qRT-PCR

Total RNA from cell lines was isolated using the RNeasy Mini kit (cat #74104, Qiagen), according to the manufacturer’s instructions. qRT-PCR was performed as described elsewhere^51^ with some minor modifications: RNA was reverse transcribed to cDNA using iScript Reverse Transcription Supermix (cat # 1708841, Bio-rad). cDNA was then diluted 1:5 in nuclease free H_2_0. qRT-PCR was performed on a C1000 Touch thermal cycler (Bio-rad) and gene expression was detected with SsoAdvanced Universal SYBR Green Supermix (cat # 1725274, Bio-rad). Gene expression was determined by normalizing to GAPDH mRNA expression levels and fold expression change was calculated using the ΔΔCT method (ΔΔCT = ΔCt sample − Δct control). Primers used in qPCR are shown in Supplemental Table 9.

### Cell lines

The breast cancer cell lines, HCC70, MDA-MB-468, and MDA-MB-231 and the T-ALL cell line Jukart were acquired from ATCC (cat #CRL-2315, #HTB-132, #CRM-HTB-26, #TIB-152), and the T-ALL cell lines RPMI-8402 and TALL-1 were acquired from DMSZ (cat #290, #521). Breast cancer and T-ALL cell lines were maintained in RPMI 1640 medium (ATCC’s modification, cat #A1049101, ThermoFisher) with 10% fetal bovine serum (FBS, heat inactivated, cat #A3840402, ThermoFisher) and 1% penicillin–streptomycin (cat #15140-122, Thermo Fisher Scientific). The HEK293T cell lines used in the small compound screening assays, including the Notch reporter cell lines and counter assay cell lines, were maintained in DMEM (ThermoFisher, cat #11995065) supplemented with 1% GlutaMAX (ThermoFisher cat #35050061), 10% fetal bovine serum (FBS, heat inactivated, cat #A3840402, Thermo Fisher) and 1% penicillin–streptomycin (cat #15140-122, Thermo Fisher). The HEK293T cell line used in orthogonal assay was cultivated without addition of GlutaMAX. Accutase (cat # A6964-100ML, Sigma-Aldrich) was used to detach the cells prior to seeding on coated plates and during maintenance. All cell lines were cultivated with 5% CO_2_ at 37 degrees. C2C12 cells were obtained from Sigma-Aldrich (cat #91031101) and maintained in proliferation DMEM high glucose-based medium (ThermoFisher, cat #12430054) containing 20% FBS and 1% PenStrep (100U/ml) and were splitted 1:5 upon reaching 50% confluence. Cells were allowed to differentiate in DMEM high glucose medium containing 2% donor equine serum (HyClone cat #SH30074.02). Prior to seeding, cells were counted using automated cell counting devices. Scepter 2.0 (MerckMillipore) was used for counting cells for small compound screens and Bio-rad’s TC20 cell counter was used for cell counting in orthogonal assays.

### Transfection of cells and generation of clones

Cells were transfected using Lipofectamine 3000 (ThermoFisher, cat #L3000001), according to the manufacturer’s instructions. Two days post transfection, cells were detached, counted, diluted and single cells were seeded into 96-well plates. Seven days post seeding, wells containing monoclonal colonies were labelled and medium was changed. Clones were cultivated to 90% confluence and then further expanded.

### Screening of the small compound library

37,966 compounds were spotted by the compound center at SciLifeLab (https://www.scilifelab.se/) on a total of 120 plates (Greiner #781080) at a fixed concentration of 10 µM and the screening process spanned five consecutive sessions. The layout of each plate contained one column for DMSO and one for DAPT controls to determine the quality and reliability of the data. DAPT controls were spotted at 10 µM and DMSO was spotted in an equimolar volume. In addition, plates spotted with only DMSO were utilized to investigate the impact of plate effects before and after each session. Furthermore, an alternative set of controls, including several Notch inhibitors and GSIs, was used to verify the functionality of the assay and to ensure that its performance aligned with the expected dynamic downregulation upon blockage of Notch. Compound-spotted plates were stored at -20 °C and were equilibrated to room temperature, followed by one minute centrifugation at 1000 × g before seeding of cells. HEK293T Notch reporter cells were grown to 75% confluence, detached and 20 µl containing 4,000 cells were seeded in each well of a 384-well compound plate using automated cell dispenser systems (Multidrop, ThermoFisher). 24 h prior seeding, dispenser cassettes were equilibrated, and tubing was thoroughly flushed with several washes of PBS followed by medium. Tubing was left in medium for 10 min before seeding cells to equilibrate conditions and ensure even dispensing. Cells seeded on compound plates were cultivated for 24 h and placed in a box within the cell incubator with additional tissues soaked in sterile H_2_0 to avoid evaporation from compound plates. Luciferase reporter activity was assessed using the dual-GLO luciferase kit from Promega (cat #E2940). First, three compound plates per time were allowed to equilibrate to room temperature. Next, 20 µl firefly luciferase substrate was added to each well using an automated dispenser systems (Multidrop, ThermoFisher) followed by incubation with thorough agitation for 10 min in the dark. Firefly luciferase signals were then measured on a Wallac Victor3 1420 plate reader (Perkin Elmer) with a signal integration time of 0.5 s. After reading firefly signals of all compound plates, the dispenser cassette was changed to avoid contamination between the different reporter signals. Next, 20 µl of renilla firefly substrate was added as before and plates were incubated for 10 min in the dark while shaking. Subsequently plates were incubated for an additional 10 min in the dark before signals were read. Activity of compounds was assessed by calculating the ratio of firefly and renilla for each well, followed by normalising each value to the averaged DMSO control values of each plate. Normalised DMSO and DAPT control values were determined, and the respective standard deviation was then used to calculate the Z-factor for each plate to determine the quality of the screen and assay window. A Z-factor of > 0.5 was considered excellent.

Inhibitor hits were selected by setting the threshold close to a theoretical exclusion criterium. The threshold was defined by first calculating the sum of the three-fold standard deviation (SD) for normalized DMSO respectively DAPT controls, averaged across all plates. The sum of the averaged inhibition by DAPT and the combined three-fold SDs of DAPT and DMSO was then calculated. This theoretical criterium threshold was 72%, meaning that compounds that do not reduce the signal to more than 72% of DMSO should be excluded. The hit threshold was set to 70%. The three-dose compound confirmation screen (1.25, 10, 25 µM) was conducted using the same conditions as in the primary screen.

### The APP reporter-based counter screen

For the APP three dose (1.25, 10, 25 µM) counter dose response assays HEK293T wild type cells were transiently transfected with three different plasmids coding for the components described. Plasmids for APP and luciferase are described in^24^ and the renilla plasmid described above generated for this project was used to normalize firefly values. 24 h post transfection cells were seeded on compound plates and cells were cultivated for 24 h before luciferase signals were read, as described.

### Dose–response analysis

Compounds for the 11-point (4.00E−05, 2.00E−05, 1.00E−05, 5.00E−06, 2.50E−06, 1.25E−06, 6.25E−07, 3.12E−07, 1.56E−07, 7.81E−08, 3.91E−08M) dose response assays (APP reporter assay and Notch reporter assay) were distributed by two step serial dilutions from compound stocks in master plates using a Bavo automated liquid handling system (Agilent). Two-fold concentrations of compounds were diluted in 10 µl cell culture medium and brought to one-fold by seeding 10 µl containing 4000 cells of the respective cell type on each well of a 384-well plate, as described above. Analogous to the previous assays, all plates contained one DMSO and DAPT control column with the Notch reporter cell line (#A1-11) and a DMSO only and Notch inhibitor control plate was read before the measuring the screening plates. In addition, DAPT and DMSO were spotted in a dose response fashion, and a minimum of four replicates were read. Dose-responses of compounds were recorded in triplicates for each concentration reading out the dual luciferase reporters, as described above. Dose response assays were read together in one session. To measure compound effects, the different assays dose–response curves were fitted using the log(inhibitor) vs. response – Variable slope (model: Y = Bottom + (Top–Bottom)/(1 + 10^((LogIC50-X)*HillSlope)) equation.

The 14-point dose–response analysis was conducted in a similar fashion, but compounds were spotted on plates via an Echo 650 liquid dispenser instead of diluted from stocks. As described, compounds were sorted into three different baskets corresponding to different dose-ranges (concentration for compounds in basket #1: 4.00E−05, 1.33E−05, 4.44E−06, 1.48E−06, 4.94E−07, 1.65E−07, 5.49E−08, 1.83E−08, 6.10E−09, 2.03E−09, 6.77E−10, 2.26E−10, 7.53E−11, 2.51E−11M, basket #2: 4.00E−05, 2.00E−05, 1.00E−05, 5.00E−06, 2.50E−06, 1.25E−06, 6.25E−07, 3.13E−07, 1.63E−07, 7.50E−08, 3.75E−08, 2.50E−08, 1.25E−08, 4.88E−09M basket #3: 5.00E−06, 1.25E−06, 5.63E−07, 1.88E−07, 6.25E−08, 2.50E−08, 6.88E−09, 2.25E−09, 7.50E−10, 2.50E−10, 8.50E−11, 2.75E−11 1.00E−11, 2.50E−12M). A DMSO plate was read before measuring luciferase activity of compound spotted plates. APP firefly luciferase reporter assays were read with a signal integration time of 0.1 s, instead of 0.5 s used for the Notch reporter to reduce signal overlap between wells. IC50 values and dose response curves were fitted as described, but the bottom of the assay was constrained to the maximum inhibition recorded with DAPT (or RO492097) or the GSI LY-411575 (APP counter assay) for each plate to define a more precise assay window. The full assay window is shown for compounds potentially exceeding the bottom constraint. The area-under-the-curve (AUC) was calculated on dose–response curves using Graphpad Prism.

### The C2C12 myogenic assay

8000 C2C12 cells were seeded on gelatin (0.1%, Sigma-Aldrich, cat #G2500) coated cover slides (BD Bioscience) and cultivated in proliferation medium. For gelatin coating, 8-well cover slide chambers (BD Bioscience) were incubated for 60 min with 0.1% gelatin in PBS. The remaining liquid was aspirated, and gelatin was allowed to dry for 24 h. Cover slides were stored at 4 °C until use. Upon reaching 85% confluence cell culture medium was exchanged to differentiation medium to enhance differentiation into myocytes/myotubes. Cells were then treated with 10 µM of the respective compound for five days and DMSO-treated cells served as a base-line control. Next, cells were washed with 1 × cold PBS (ThermoFisher, cat #10010023) fixed with 4% paraformaldehyde (PFA, Sigma-Aldrich, cat #1.00496) in PBS for 10 min at room temperature and washed again with 1 × PBS. Fixed cells were either stored at 4 °C in PBS or directly processed for immunocytochemistry staining: For immunocytochemistry, cells were permeabilized for 10 min in 0.1% TritonX-100 / PBS, then washed in PBS and unspecific bindings sites were blocked in protein free blocking solution (DAKO, cat # × 0909) for 30 min at room temperature, followed by two times washing in PBS. Then, cells were incubated with primary antibody MYH4 (ThermoFisher) in blocking solution and 0.1% TritonX-100 in PBS for 1 h at room temperature. Next, cells were washed three times in PBS and incubated with secondary Alexa fluorophore coupled antibody (Invitrogen) in a 1:400 dilution. Labeling of F-actin filaments was conducted by phalloidin-TRITC (Sigma-Aldrich, cat # P1951) in a 1:2500 dilution. Cells were washed three times in PBS and incubated with DAPI (BD Bioscience cat # 564907) for 5 min at room temperature to stain DNA/nuclei. Finally, slides were fixed with mounting medium (ProLong Antifade mounting medium, ThermoFisher, cat #P36934). Bright field and immunocytochemistry images were taken using the SP8 LSM (Leica) at different magnifications and images were recorded as Z-stacks. Images were processed using the FijI (ImageJ) software. As controls, wells with and without differentiation medium were also stained to set the baseline and contrast the differences and stainings of cells with only secondary antibodies were used as negative controls to assess cross-reactivity and background staining. For calculating the MYH4 median fluorescence intensity (MFI) images have been converted into 8-bit greyscale and a standard color threshold was applied (ImageJ). Next, MFI signals per area were measured using ImageJ and divided by the median background intensity (MBI). The nuclei fusion index was calculated as the ratio of nuclei fused per area divided by the total number of nuclei in the field. A minimum of three images has been recorded for the calculations, and two replicate experiments were conducted. For calculations of MFI and fusion index see Supplemental Table 5.

### Three dose response gene expression analysis

Adherent breast cancer cells were seeded in 24-well cell culture plates (ThermoFisher, Nunc, Delta surface, cat #144530) and T-ALL cells growing in suspension (Jurkat, RPMI-8402) were seeded in non-adherent 24-well plates (ThermoFisher, Nunc, cat #144530). 60.000 cells were seeded and treated with 0.1, 1 and 10 µM of each compound. As controls, CB-103 (a non-GSI Notch inhibitor) and the GSI RO4929097 were used with the same dose regimen. Cells were harvested 24 h post treatment and RNA isolation and gene expression analysis (qRT-PCR) was performed as described above.

### Growth inhibition in tumour and HEK293T cell lines

Metabolic activity in different tumour and HEK293T cell lines was assessed in 14-point dose response regimens (5.00E−05, 2.50E−05, 1.25E−05, 5,00E−06, 2.50E−06, 1.25E−06, 5.00E−07, 2.50E−07, 1.25E−07, 5.00E−08, 2.50E−08, 1.25E−08, 5.00E−09, 2.50E−09M). Compounds were spotted on white 384-well tissue culture plates (Greiner, cat #781080) using an Echo 525 Acoustic liquid handler (Labcyte). Cells were grown to 80% confluence, detached if required, counted and 20 µl containing 2000–4000 cells per well was then seeded in each well and cultivated for three (4,000 cells seeded) and six days (2,000 cells seeded). As a control, a minimum of 24 wells containing cells incubated with only DMSO at the same concentration as when the GSI RO4929097 or the Notch inhibitor CB-103 were included. Luciferase activity (reading out metabolic activity) was determined using the CellTiter-Glo 2.0 kit (CTG, Promega, cat #G9242) following the manufacturer’s instructions and luminescence was measured on a SpectraMaxi3 or Varioscan plate reader (Molecular Devices, Thermofisher).

### Immunocytochemistry

20,000 MDA-MB-231 cells were seeded on cover-slides (BD Bioscience), allowed to attach and treated with 2 µM compound for 24 h and 72 h upon reaching 80% confluence. For quantification of NOTCH1 signals and to monitor morphological changes immunocytochemistry staining to localize NOTCH1 was carried out as described above for C2C12 cells. Cellular morphology was visualized with Phalloidin (Thermofisher, cat # A22287), used at 1:2000 dilution and a NOTCH1 antibody used at 1:100 dilution (Cell signalling, cat #D6F11). Images were recorded using a Zeiss LSM 700 laser scanning microscope with a 40 × objective. Identical channel configuration and detector gain settings were used for quantification of fluorescence signals and a minimum of four images were recorded per condition. Secondary antibody control background signals were averaged and subtracted from each image and the same brightness and contrast adjustments were applied to all images before conducting histogram-based quantification of NOTCH1 signals. A 10 × objective was used for assessing morphological changes and a minimum of four and three images at 24 h and 72 h, respectively were recorded for quantification of nuclei count, size and number of giant cells. Images were processed using Fiji (ImageJ) software and objects were separated from background using Otsu thresholding until all nuclei were detectable. Nuclei objects were filled and nuclei particles were measured using size exclusion and circularity filter settings, 10-infinity (size) and 0,1–0,5 (circularity). Overlay images of nuclear stain and Phalloidin were used to manually count and annotate giant cells using Fiji’s cell counter.

### Cycloheximide chase experiments

HEK293T Notch reporter cells (clone #A1-11) were grown to 80% confluence, detached and counted. 50,000 cells were then seeded in each well of a 24-well tissue culture plate (Sarstedt, cat # 83.3924), cultivated to approximately 85% confluence and treated with 5 µM of the respective compound and 50 µg/ml Cycloheximide (CHX) solution (Sigma-Aldrich, cat #C4859-1ML). Each well was then incubated with 500 µl of compound and CHX solution and compound and CHX solution were added after the indicated intervals to another well for a total time of eight hours. Next, medium was aspirated off and cells were lysed in RIPA buffer (Thermofisher, cat #89900) containing Protease and Phosphatase inhibitors (Thermofisher, cat #78442) for 30 min on ice. Lysates were thoroughly vortexed, centrifugated for 10 min at > 13,000×*g* and supernatant was transferred to a fresh cup and stored at -80 °C.

### DHODH inhibitor and uridine rescue experiment

HEK293T Notch reporter cells (#A1-11) were grown to 80% confluency, detached and mixed with the indicated compounds and doses with or without uridine (Sigma-Aldrich, cat #U3003). 10,000 cells/well were then seeded into 96-well cell culture plates (ThermoFisher, cat #136102) and incubated for 24 h. DMSO treatment with or without uridine served as control for each treatment. Cells were then subjected to Notch reporter assays, and luciferase signals were recorded on a Varioscan Lux microplate reader (ThermoFisher). Reporter signals from each treatment were normalised to the respective DMSO control, as described above and three independent assays were recorded with at least two technical replicates per treatment.

### Cell surface biotinylation experiment

For labelling of membrane proteins, MDA-MB-231 cells were grown to 80% confluency in 6-well cell culture dishes (ThermoFisher, cat #104675) and treated with 2 µM of the indicated compounds for 24 h. Plates were then put on ice for 10 min to slow down cellular metabolism and halt internalisation of membrane proteins. Next, 0.5 mg/ml Sulfo-NHS-Biotin (ThermoFisher, cat #21217) in PBS was added to every second well for 40 min and non-treated wells served as controls. Excess of biotin was removed by washing the plates with PBS followed by incubation with 100 mM glycin (Sigma-Aldrich, cat #G8898) in PBS on ice. Wells were washed twice with ice-cold PBS and next, 500 µl lysis buffer was added to each well (50 mM Tris–HCl pH 7.4, 150 mM NaCl, 1% Triton X-100, (Sigma-Aldrich, cat #X100), 2 mM EDTA, including protease and phosphatase inhibitors, Thermofisher, cat #78442). Lysates were thoroughly vortexed, centrifugated at 14,000xg for 10 min at 4 °C and 50 µl of each supernatant was taken as input fraction and stored until further use at -80 °C. NeutrAvidin Agarose beads were pre-equilibrated in lysis buffer for 5 min and 50 µl was added to each sample. Pull-down fractions were incubated on an end-to-end rotator over night at 4 °C. Next, beads were washed three times in lysis buffer for at least 10 min per wash and dry beads were mixed with 4 × Laemmli buffer (Bio-rad, cat #1610747) and stored at -80 °C until use. NOTCH1 TMICD western blot band intensity was quantified using Fiji (ImageJ) after background subtraction. Relative enrichment of NOTCH1 protein present at the cell surface was calculated with IP/Input ratio (IP NOTCH1/Input NOTCH1). Input and corresponding IP fraction of a treatment were separated on the same gel and bands were measured while non-saturated. Two independent biological replicates were assessed.

### HiBit reporter assay

20 µl containing 4,000 cells were seeded on 384-well pre-spotted compound plates (Greiner, cat #781080). Cells were incubated for 24 h and viability was determined using CellTiter-Fluor (Promega, cat # G6080), following the manufacturer’s instructions. Next, cells were subjected to the HiBit assay according to the manufacturer’s instructions (Promega, cat # N3030). Prior to cell lysis, the tissue culture plates were allowed to cool down to room temperate. Fluorescence and luminescence signals were recorded using a SpectraMax i3 (Molecular devices) and signals were read at different time-points to determine optimal signal stability. Cells cultivated only with DMSO were used as a reference to determine effects of compounds. MG132, Bafilomycin A1 and the GSI RO4929097 served as additional controls. Celastrol was used to determine the assay window, and a minimum of two technical replicates were measured per assay.

### Western blot analysis

Cells were washed in ice-cold PBS and incubated for 30 min on ice with RIPA cell lysis buffer (Thermofisher, cat #89900) containing protease and phosphatase inhibitors (Thermofisher, cat #78442), scraped off and homogenized using a 25G syringe, and subsequently centrifugated for 10 min at > 13,000xg. The supernatant was mixed with 4 × Laemmli buffer (Bio-rad, cat #1610747) and heated for 5 min to 95 °C and either loaded on MiniProtean Tris–Glycine TGX gradient gels (Biorad) or stored at -80 °C until further use. Protein lysates were separated on MiniProtean 4–15% or 4–20% Tris–Glycine TGX gradient gels (Bio-rad). After electrophoresis, gels were placed directly onto a TransBlot Turbo PVDF or nitrocellulose membrane (Bio-rad, cat #1704157 and cat #1704159) and transferred using the “HIGH MW” protocol (Bio-Rad). Subsequently, membranes were blocked for 1 h at room temperature using Clear Milk Blocking Buffer (ThermoFisher, cat #37587) supplemented with 0.1% Tween20 (Sigma-Aldrich, cat #P1379). Detection of antibody binding was performed by using Clarity Western ECL-Substrate (Bio-rad, cat #1705060S). Beta-Actin protein signals were used as a loading control. Western blot membranes were in some experiments stripped after development (indicated in the figure in each respective case) by washing the membranes twice for 10 min in PBS, followed by incubation in membranes in western blot stripping buffer (ThermoFisher, cat #21059) for 30 min at room temperature and gentle agitation. Stripped membranes were washed twice with PBS and incubated for 30 min in blocking buffer before incubation with primary antibody. A list of all antibodies used in this study is provided in Supplemental Table 6. Uncropped western blot images are provided in Supplemental Fig. 8. Protein expression levels of western blot bands were quantified using *Fiji* (V.2.16) with beta-Actin signals for normalisation (Supplemental Table 8). Uncropped western blot images are provided in Supplemental Fig. 10.

### DHOHD assay

DHODH enzyme activity was determined via fluorescence-based quantification (Resorufin fluorescence as endpoint measurement) at *Reaction Biology*. Reference compound BAY2402234 was measured in singletons in two biological replicates and compounds were measured in technical replicates within the indicated dose-ranges. Values were normalized to DMSO control and dose–response curves were fitted using the log(inhibitor) versus response – Variable slope (model: Y = Bottom + (Top–Bottom)/(1 + 10^((LogIC50-X)*HillSlope)) equation.

### Data analysis and visualisation

Statistical analyses were performed using *GraphPad Prism* (V8-10). ImageJ/Fiji (V2.16) was used to process microscopy images. Figures were created using *Adobe Photoshop* (2024/2025) and *Adobe Illustrator* (2023–2024). Labelling of plots and graphs, changes to axis thickness and overall polishing were done in *Adobe illustrator* to improve clarity and readability. Datapoints were not changed. Western blot images were processed with Bio-rad’s Image Lab or Invitrogen’s iBright Image Software (V5.4).

## Supplementary Information

Supplementary Information 1.

Supplementary Information 2.

Supplementary Information 3.

Supplementary Information 4.

Supplementary Information 5.

Supplementary Information 6.

Supplementary Information 7.

Supplementary Information 8.

Supplementary Information 9.

Supplementary Information 10.

Supplementary Information 11.

Supplementary Information 12.

Supplementary Information 13.

Supplementary Information 14.

Supplementary Information 15.

Supplementary Information 16.

## Data Availability

Data generated and supporting the findings of this study are provided within the article and its supplemental information files. Additional data, such as raw small molecule compound screen data, cell lines generated or hit compounds are available upon reasonable requests.

## References

[1] Bray, S. J. Notch signalling in context. *Nat. Rev. Mol. Cell Biol.* **9**, 722–735 (2016). 10.1038/nrm.2016.94 27507209

[2] Siebel, C. & Lendahl, U. Notch signaling in development, tissue homeostasis, and disease. *Physiol. Rev.* **97**, 1235–1294 (2017). 28794168 10.1152/physrev.00005.2017

[3] Aster, J. C., Pear, W. S. & Blacklow, S. C. The varied roles of Notch in cancer. *Annu. Rev. Pathol. Mech. Dis.* **12**, 245–275 (2017). 10.1146/annurev-pathol-052016-100127 PMC5933931 27959635

[4] Braune, E., Seshire, A. & Lendahl, U. Notch and Wnt dysregulation and its relevance for breast cancer and tumor initiation. *Biomedicines* **6**, 101 (2018). 30388742 10.3390/biomedicines6040101 PMC6315509

[5] Braune, E.-B. & Lendahl, U. Notch: A goldilocks signaling pathway in disease and cancer therapy. *Discov. Med.* **21** (2016). 27115169

[6] Hurtado, C. et al. Disruption of NOTCH signaling by a small molecule inhibitor of the transcription factor RBPJ. *Sci. Rep.* **9**, 10811 (2019). 31346210 10.1038/s41598-019-46948-5 PMC6658660

[7] Lehal, R. et al. Pharmacological disruption of the Notch transcription factor complex. *Proc. Natl. Acad. Sci. USA* **117**, 16292–16301 (2020). 32601208 10.1073/pnas.1922606117 PMC7368267

[8] Alvarez-Trotta, A. et al. Pharmacological disruption of the Notch1 transcriptional complex inhibits tumor growth by selectively targeting cancer stem cells. *Cancer Res.* **81**, 3347–3357 (2021). 33820800 10.1158/0008-5472.CAN-20-3611 PMC8655881

[9] Diluvio, G. et al. A novel chemical attack on Notch-mediated transcription by targeting the NACK ATPase. *Mol. Ther. Oncolytics.* **28**, 307–320 (2023). 36938545 10.1016/j.omto.2023.02.008 PMC10015116

[10] Krämer, A. et al. Small molecules intercept Notch signaling and the early secretory pathway. *Nat. Chem. Biol.* **9**, 731–738 (2013). 24077179 10.1038/nchembio.1356

[11] Lu, Z. et al. FLI-06 suppresses proliferation, induces apoptosis and cell cycle arrest by targeting LSD1 and Notch pathway in esophageal squamous cell carcinoma cells. *Biomed. Pharmacother.* **107**, 1370–1376 (2018). 30257352 10.1016/j.biopha.2018.08.140

[12] Wu, Y. et al. Therapeutic antibody targeting of individual Notch receptors. *Nature* **464**, 1052–1057 (2010). 20393564 10.1038/nature08878

[13] Chung, J. et al. Fibroblastic niches prime T cell alloimmunity through delta-like Notch ligands. *J. Clin. Invest.* **127**, 1574–1588 (2017). 28319044 10.1172/JCI89535 PMC5373885

[14] Lafkas, D. et al. Therapeutic antibodies reveal Notch control of transdifferentiation in the adult lung. *Nature* **528**, 127–131 (2015). 26580007 10.1038/nature15715

[15] Wei, K. et al. Notch signalling drives synovial fibroblast identity and arthritis pathology. *Nature* **582**, 259–264 (2020). 32499639 10.1038/s41586-020-2222-z PMC7841716

[16] Tran, I. T. et al. Blockade of individual Notch ligands and receptors controls graft-versus-host disease. *J. Clin. Invest.* **123**, 1590–1604 (2013). 23454750 10.1172/JCI65477 PMC3613915

[17] Yan, M. et al. Chronic DLL4 blockade induces vascular neoplasms. *Nature* **463**, E6-7 (2010). 20147986 10.1038/nature08751

[18] Andersson, E. R. & Lendahl, U. Therapeutic modulation of Notch signalling--Are we there yet?. *Nat. Rev. Drug Discov.* **13**, 357–378 (2014). 24781550 10.1038/nrd4252

[19] Majumder, S. et al. Targeting Notch in oncology: the path forward. *Nat. Rev. Drug Discov.* **20**, 125–144 (2021). 33293690 10.1038/s41573-020-00091-3

[20] Keam, S. J. Nirogacestat: First approval. *Drugs* **84**, 355–361 (2024). 38409573 10.1007/s40265-024-02002-x

[21] Wu, S. et al. PiggyBac is a flexible and highly active transposon as compared to Sleeping Beauty, Tol2, and Mos1 in mammalian cells. *Proc. Natl. Acad. Sci. U. S. A.* **103**, 15008–15013 (2006). 17005721 10.1073/pnas.0606979103 PMC1622771

[22] Chapman, G., Liu, L., Sahlgren, C., Dahlqvist, C. & Lendahl, U. High levels of Notch signaling down-regulate Numb and Numblike. *J. Cell Biol.* **175**, 535–540 (2006). 17116748 10.1083/jcb.200602009 PMC2064589

[23] Beresini, M. H. et al. Small-molecule library subset screening as an aid for accelerating lead identification. *SLAS Discov.* **19**, 758–770 (2014). 10.1177/1087057114522515 24518067

[24] Karlström, H., Bergman, A., Lendahl, U., Näslund, J. & Lundkvist, J. A sensitive and quantitative assay for measuring cleavage of presenilin substrates. *J. Biol. Chem.* **277**, 6763–6766 (2002). 11744687 10.1074/jbc.C100649200

[25] Walters, W. P. & Namchuk, M. Designing screens: How to make your hits a hit. *Nat. Rev. Drug Discov.* **2**, 259–266 (2003). 12669025 10.1038/nrd1063

[26] Vargas-Franco, D. et al. The Notch signaling pathway in skeletal muscle health and disease. *Muscle Nerve* **66**, 530–544. 10.1002/mus.27684 (2022). 35968817 10.1002/mus.27684 PMC9804383

[27] Dahlqvist, C. et al. Functional Notch signaling is required for BMP4-induced inhibition of myogenic differentiation. *Development* **130**, 6089–6099 (2003). 14597575 10.1242/dev.00834

[28] Lolli, M. L. et al. New inhibitors of dihydroorotate dehydrogenase (DHODH) based on the 4-hydroxy-1,2,5-oxadiazol-3-yl (hydroxyfurazanyl) scaffold. *Eur. J. Med. Chem.* **49**, 102–109 (2012). 22245049 10.1016/j.ejmech.2011.12.038

[29] Leban, J., Saeb, W., Garcia, G., Baumgartner, R. & Kramer, B. Discovery of a novel series of DHODH inhibitors by a docking procedure and QSAR refinement. *Bioorg. Med. Chem. Lett.* **14**, 55–58 (2004). 14684297 10.1016/j.bmcl.2003.10.021

[30] Madak, J. T., Bankhead, A., Cuthbertson, C. R., Showalter, H. D. & Neamati, N. Revisiting the role of dihydroorotate dehydrogenase as a therapeutic target for cancer. *Pharmacol. Ther.* **195**, 111–131. 10.1016/j.pharmthera.2018.10.012 (2019). 30347213 10.1016/j.pharmthera.2018.10.012

[31] Thörig, G. E. W., Heinstra, P. W. H., de Ruiter, B. L. A. & Scharloo, W. The effects of recessive lethal Notch mutations of *Drosophila melanogaster* on flavoprotein enzyme activities whose inhibitions cause Notch-like phenocopies. *Biochem. Genet.* **25**, 7–25 (1987). 3107544 10.1007/BF00498948

[32] Ladds, M. J. G. W. et al. A DHODH inhibitor increases p53 synthesis and enhances tumor cell killing by p53 degradation blockage. *Nat. Commun.*10.1038/s41467-018-03441-3 (2018). 29549331 10.1038/s41467-018-03441-3 PMC5856786

[33] Yamamoto, A., Tagawa, Y., Yoshimori, T. & Moriyama, Y. Bafilomycin A1 prevents maturation of autophagic vacuoles by inhibiting fusion between autophagosomes and lysosomes in rat hepatoma cell line, H-4-II-E cells. *Cell Struct. Funct.* **23**, 33–42 (1998). 9639028 10.1247/csf.23.33

[34] Sexauer, A. N. et al. DHODH: a promising target in the treatment of T-cell acute lymphoblastic leukemia. *Blood Adv.* **7**, 6685–6701 (2023). 37648673 10.1182/bloodadvances.2023010337 PMC10641474

[35] He, D. et al. De novo pyrimidine synthesis fuels glycolysis and confers chemoresistance in gastric cancer. *Cancer Lett.* **549**, 215837 (2022). 35921972 10.1016/j.canlet.2022.215837

[36] Landor, S. K. J. et al. PIM-induced phosphorylation of Notch3 promotes breast cancer tumorigenicity in a CSL-independent fashion. *J. Biol. Chem.* **296**, 100593 (2021). 33775697 10.1016/j.jbc.2021.100593 PMC8100066

[37] Santio, N. M. et al. Phosphorylation of Notch1 by Pim kinases promotes oncogenic signaling in breast and prostate cancer cells. *Oncotarget* **7**, 43220–43228 (2016). 27281612 10.18632/oncotarget.9215 PMC5190019

[38] Oberg, C. et al. The Notch intracellular domain is ubiquitinated and negatively regulated by the mammalian Sel-10 homolog. *J. Biol. Chem.* **276**, 35847–35853 (2001). 11461910 10.1074/jbc.M103992200

[39] Antfolk, D., Antila, C., Kemppainen, K. & Landor, S. K. Decoding the PTM-switchboard of Notch. *BBA Mol. Cell Res.* **1866**, 118507 (2019). 10.1016/j.bbamcr.2019.07.002 PMC7116576 31301363

[40] Yun, J. et al. p53 modulates Notch signaling in MCF-7 breast cancer cells by associating with the Notch transcriptional complex via MAML1. *J. Cell. Physiol.* **230**, 3115–3127 (2015). 26033683 10.1002/jcp.25052 PMC4549197

[41] Jin, S. et al. Non-canonical Notch signaling activates IL-6/JAK/STAT signaling in breast tumor cells and is controlled by p53 and IKKα/IKKβ. *Oncogene*10.1038/onc.2012.517 (2012). 23178494 10.1038/onc.2012.517 PMC3795477

[42] Tsea, I. et al. DHODH inhibition suppresses MYC and inhibits the growth of medulloblastoma in a novel in vivo zebrafish model. *Cancers (Basel)*10.3390/cancers16244162 (2024). 39766063 10.3390/cancers16244162 PMC11674817

[43] Chan, S. M., Weng, A. P., Tibshirani, R., Aster, J. C. & Utz, P. J. Notch signals positively regulate activity of the mTOR pathway in T-cell acute lymphoblastic leukemia. *Blood* **110**, 278–286 (2007). 17363738 10.1182/blood-2006-08-039883 PMC1896117

[44] Palomero, T. et al. NOTCH1 directly regulates c-MYC and activates a feed-forward-loop transcriptional network promoting leukemic cell growth. *Proc. Natl. Acad. Sci. USA* **103**, 18261–18266 (2006). 17114293 10.1073/pnas.0606108103 PMC1838740

[45] Schuhknecht, L. et al. A human metabolic map of pharmacological perturbations reveals drug modes of action. *Nat. Biotechnol.*10.1038/s41587-024-02524-5 (2025). 39875672 10.1038/s41587-024-02524-5

[46] Zhang, J. et al. DHODH inhibition modulates glucose metabolism and circulating GDF15, and improves metabolic balance. *iScience* **1**, 102494 (2021). 10.1016/j.isci.2021.102494 PMC8169992 34113829

[47] Reis, R. A. G., Calil, F. A., Feliciano, P. R., Pinheiro, M. P. & Nonato, M. C. The dihydroorotate dehydrogenases: Past and present. *Arch. Biochem. Biophys.* **632**, 175–191. 10.1016/j.abb.2017.06.019 (2017). 28666740 10.1016/j.abb.2017.06.019

[48] Nie, Z. et al. Structure-Based Discovery and Development of Highly Potent Dihydroorotate Dehydrogenase Inhibitors for Malaria Chemoprevention. *J. Med. Chem.* **68**, 590–637 (2025). 39710971 10.1021/acs.jmedchem.4c02394 PMC11726676

[49] Chinnappanna, N. K. R. et al. Recent approaches in the drug research and development of novel antimalarial drugs with new targets. *Acta Pharma.* **73**, 1–27. 10.2478/acph-2023-0001 (2023). 10.2478/acph-2023-0001 36692468

[50] Sharma, P. & Borthakur, G. Targeting metabolic vulnerabilities to overcome resistance to therapy in acute myeloid leukemia. *Cancer Drug Resist.* **6**, 567–589. 10.20517/cdr.2023.12 (2023). 37842232 10.20517/cdr.2023.12 PMC10571063

[51] Braune et al. Loss of CSL Unlocks a Hypoxic Response and Enhanced Tumor Growth Potential in Breast Cancer Cells Stem Cell Reports. Stem Cell Reports, 6, 643-651. 10.1016/j.stemcr.2016.03.004 (2016) 10.1016/j.stemcr.2016.03.004 PMC4939550 27066863

