# Supplementary material for: Disrupting Notch signalling by a small molecule inhibiting dihydroorotate dehydrogenase activity
Source: Sci Rep. 2026 Jun 6;16:17538. doi: 10.1038/s41598-026-55679-3 (PMC13242511; doi:10.1038/s41598-026-55679-3)
Supplement: Supplementary file 1 — Supplementary Information 1. [file 41598_2026_55679_MOESM1_ESM.pdf]

Supplemental Figure 1

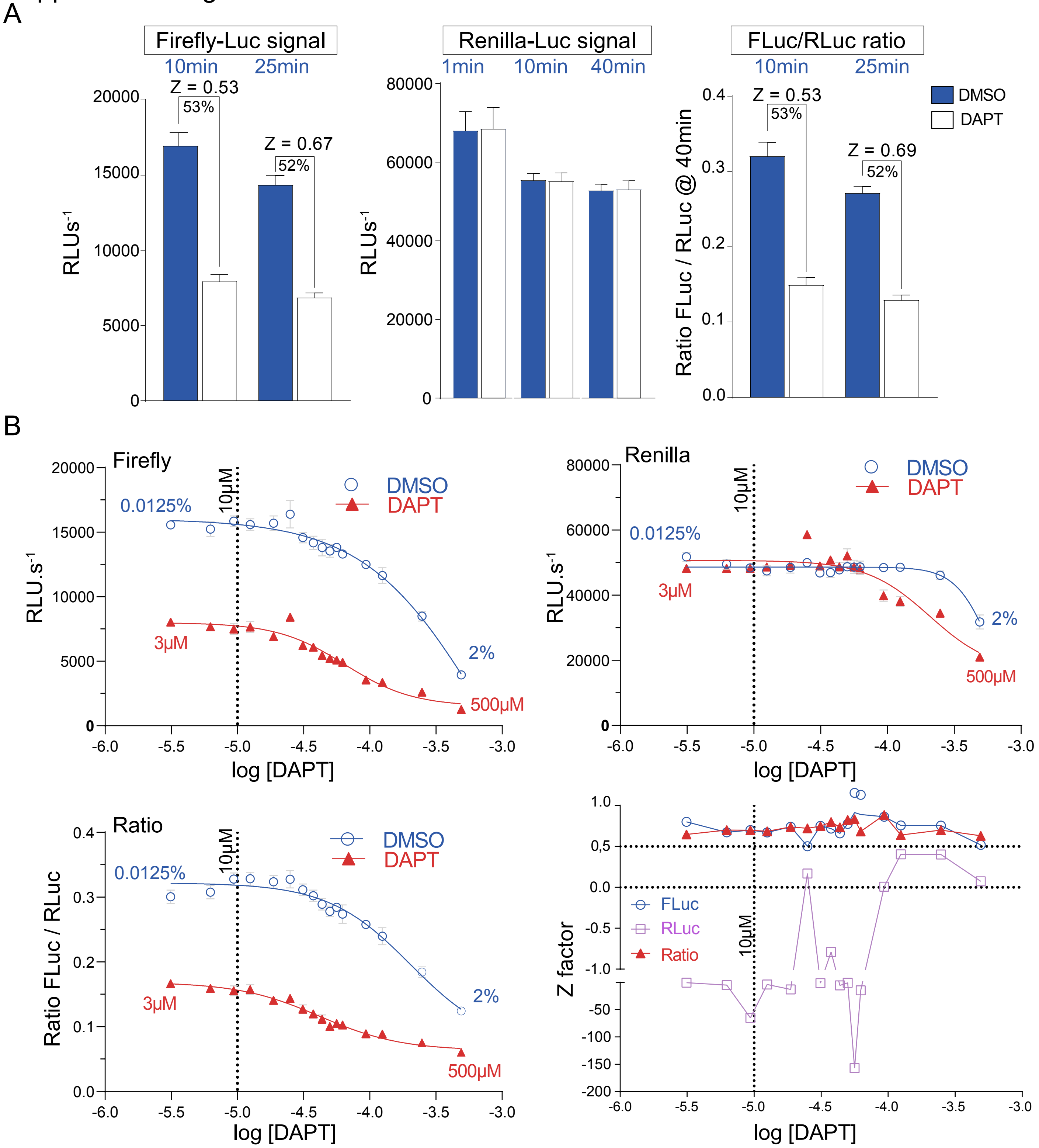

Supplemental Figure 1 continued

C

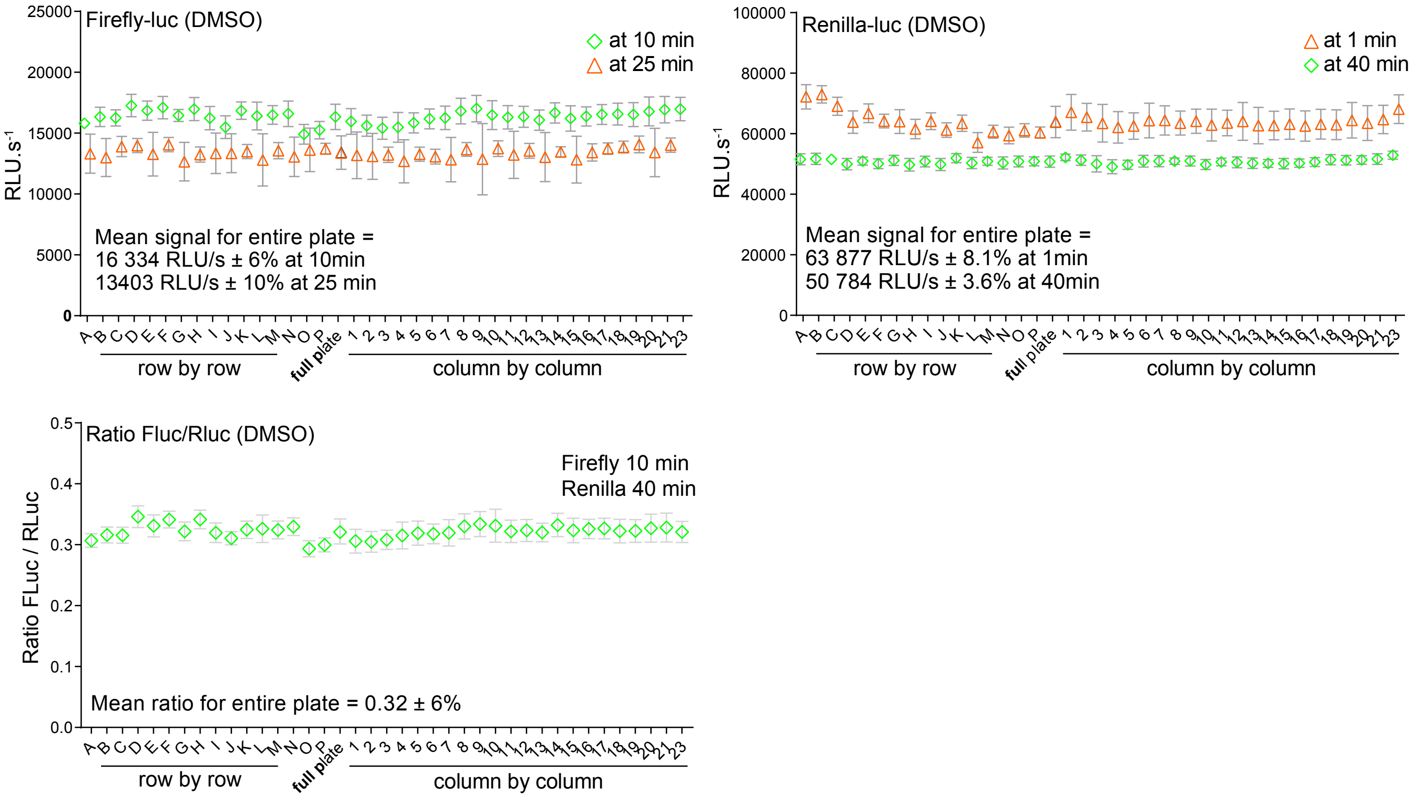

Supplemental Figure 1:

Establishing the Notch reporter assay in the 384-well format.

A) The Notch reporter (firefly) and control (renilla) luciferase activities were analysed at different timepoints (10 and 25 min for firefly and 1, 10 and 40 minutes for renilla), as indicated. The effect of DMSO versus DAPT was also analysed, as indicated. **(B)** The Notch reporter (firefly) and control (renilla) luciferase activities were analysed at different concentrations of DMSO (0.0125 to 2%) and DAPT (3 to 500 mM). The dotted line indicates the selected concentration of compounds and controls for the primary screen, 10 µM. Firefly and renilla signals are presented at the top and at the bottom the firefly/renilla luciferase ratio (left) and Z-factor of firefly, renilla and firefly/renilla ratio (right) is presented. **(C)** Signal intensities for firefly and renilla luciferase signals across DMSO plate at different timepoints, as indicated. Row numbers from A-P, column numbers from 1-23. At the bottom the firefly/renilla luciferase ratio is presented.

Supplemental Figure 2

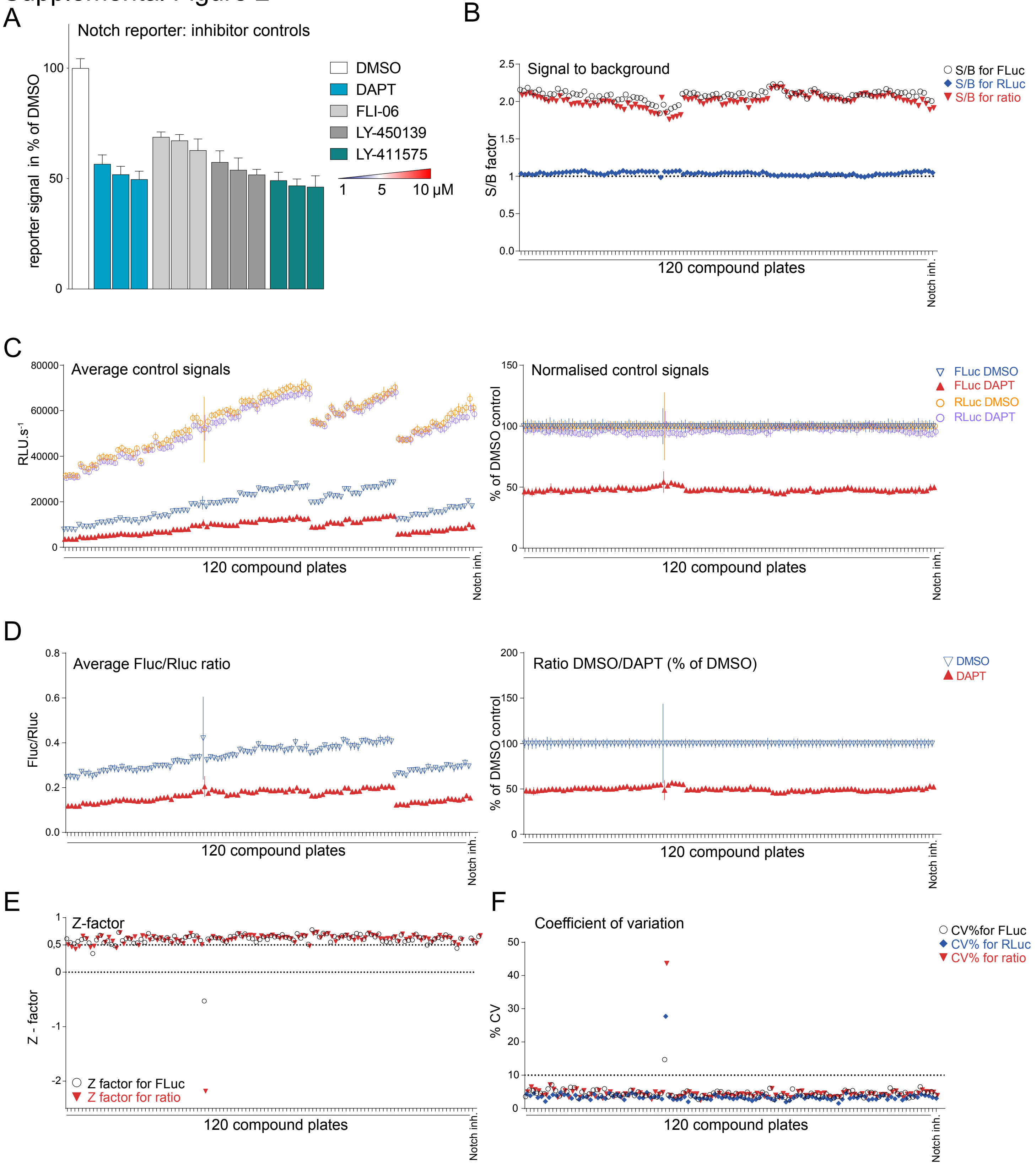

Supplemental Figure 2 continued

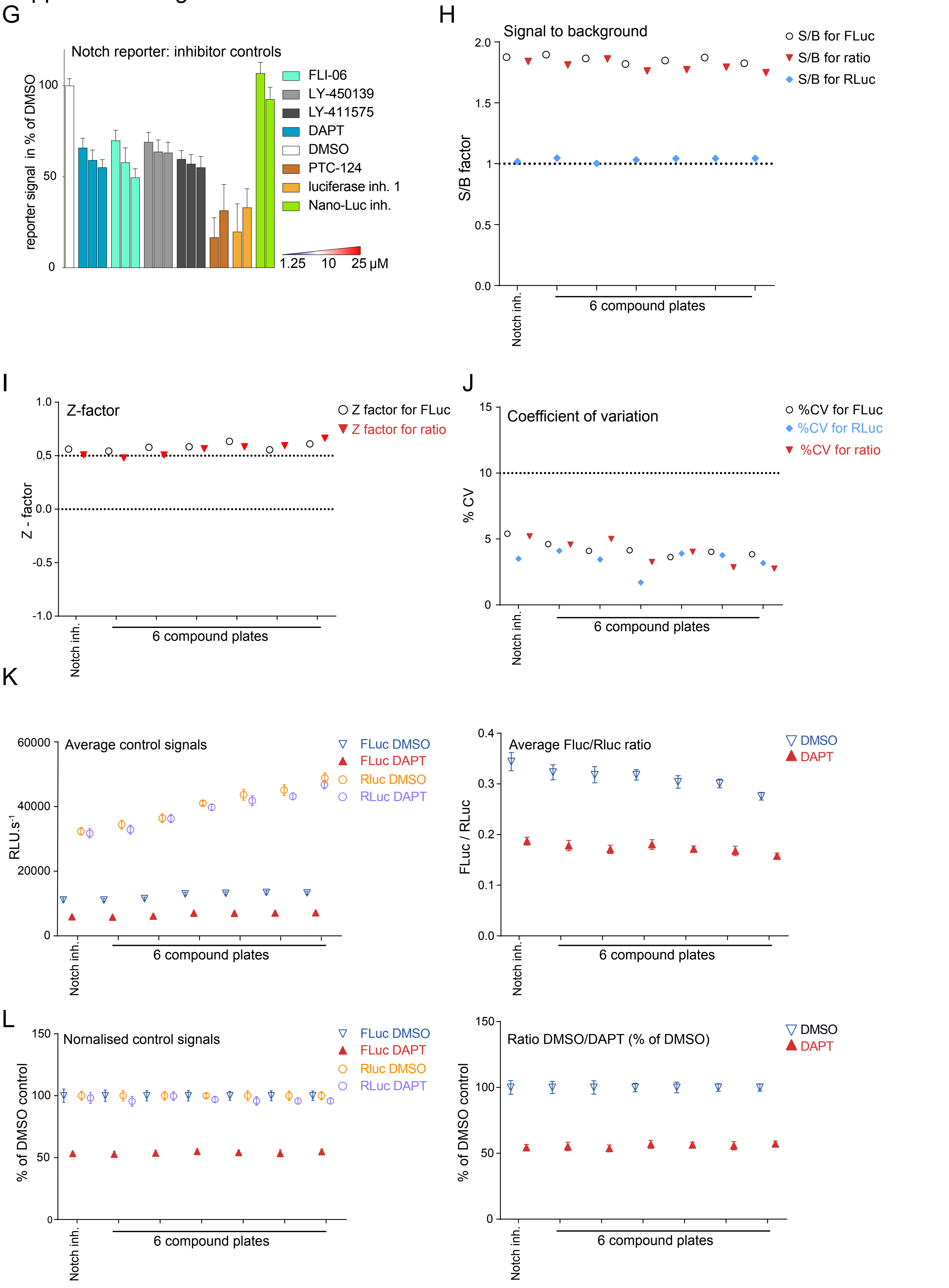

Supplemental Figure 2:

Validation of the Notch reporter assay. (A) Analysis of the effects of FLI-06, LY-450139 (Semagacestat), LY-411575 and DAPT at three doses (1, 5, 10 mM) in the Notch reporter assay. **(B)** The signal-to-background ratio of DMSO and DAPT controls across 120 plates is shown for firefly and for renilla luciferase individually and for the normalised ratio of DMSO and DAPT. **(C)** Analysis of control signals for firefly and renilla luciferase in the presence of DMSO or DAPT across 120 plates. The signals after normalization are shown below to the right. **(D)** Average (Fluc/Rluc) ratio across 120 plates. To the right, the DMSO/DAPT ratio in percent of DMSO controls across 120 plates is presented. **(E)** The Z'-factor for firefly luciferase activity and the Z'-factor for the ratio between DMSO and DAPT is shown. **(F)** The Coefficient of variation across 120 plates is shown for firefly and for renilla luciferase individually and for the normalised ratio of DMSO and DAPT. **(G)** Analysis of three luciferase inhibitors (PTC-124, luciferase inhibitor 1 and Nano-Luc inhibitor) as well as FLI-06, LY-450139, LY-411575 and DAPT in the Notch reporter assay at three different dosages (1.25, 10 and 25 mM). **(H)** The signal-to-background ratio of DMSO and DAPT control is shown for firefly and for renilla luciferase individually and for the normalised ratio of DMSO and DAPT. **(I)** The Z'-factor for firefly luciferase activity and for the ratio is shown. **(J)** The coefficient of variation is shown for firefly and for renilla luciferase and for the normalised ratio of DMSO and DAPT. **(K)** Left, average firefly and renilla luciferase signals of DMSO and DAPT controls is shown and averaged DMSO and DAPT ratio is shown to the right. **(L)** Reporter signals of DMSO and DAPT after normalization is shown to the left and the DMSO/DAPT ratio relative to the DMSO controls is shown to the right. The analysis was carried out using a total of six compound plates.

Supplemental Figure 3

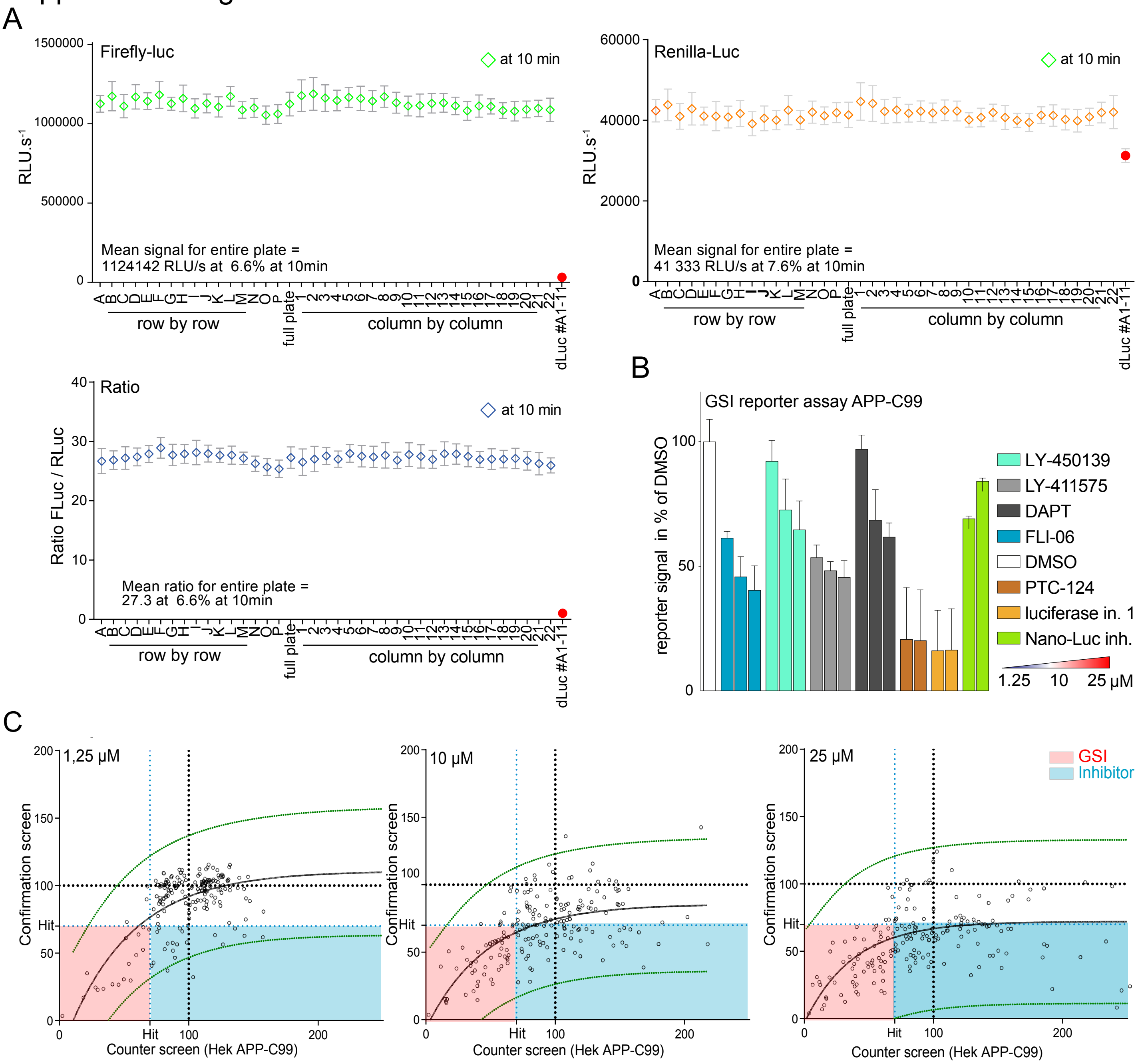

Supplemental Figure 3 continued

D

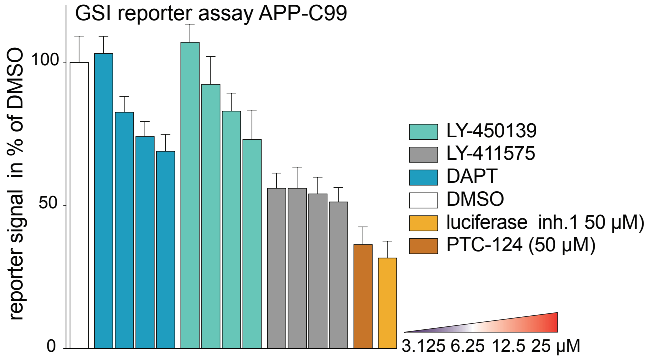

E

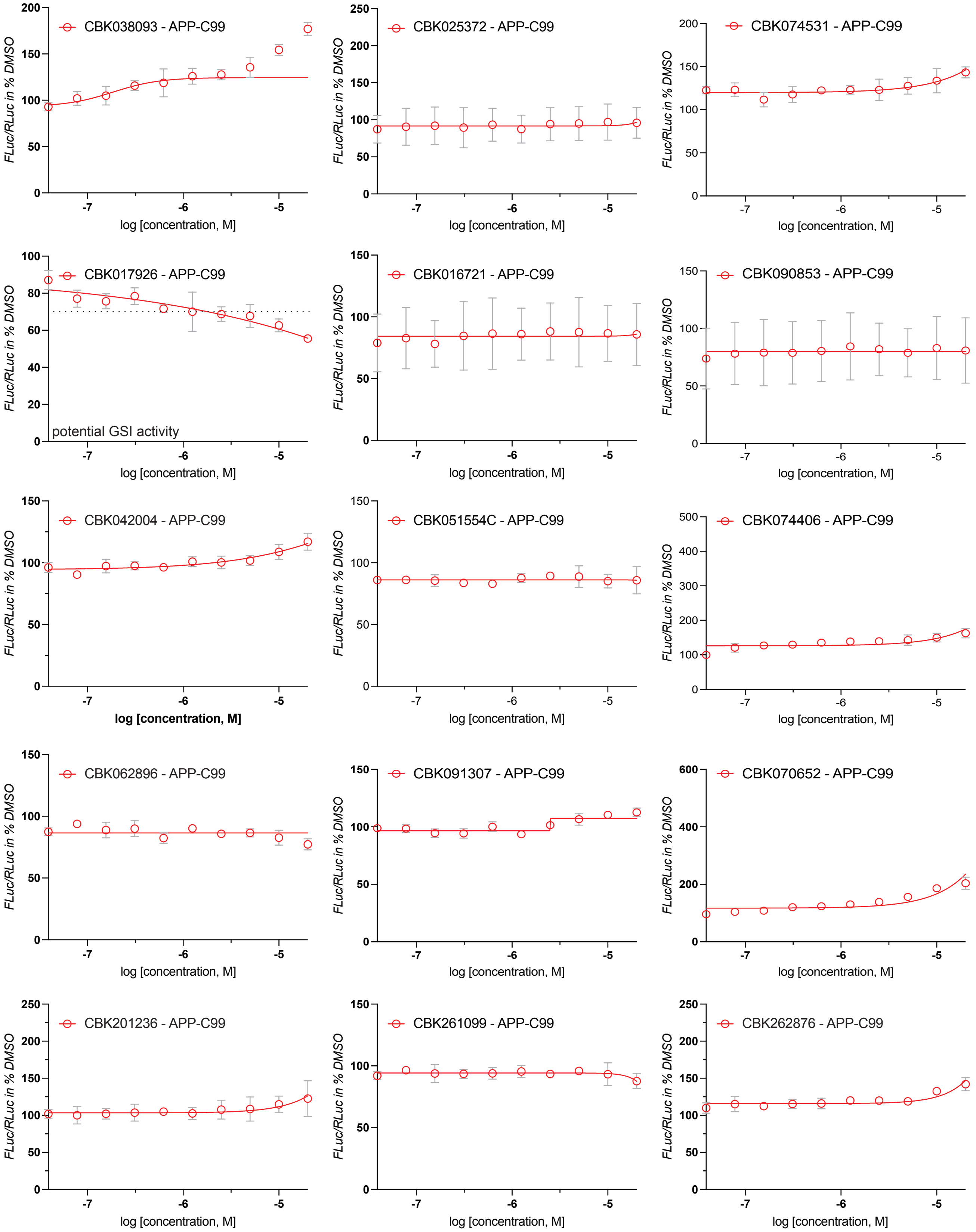

Supplemental Figure 3E continued

11-point dose-response curves: APP-C99 counter-assay

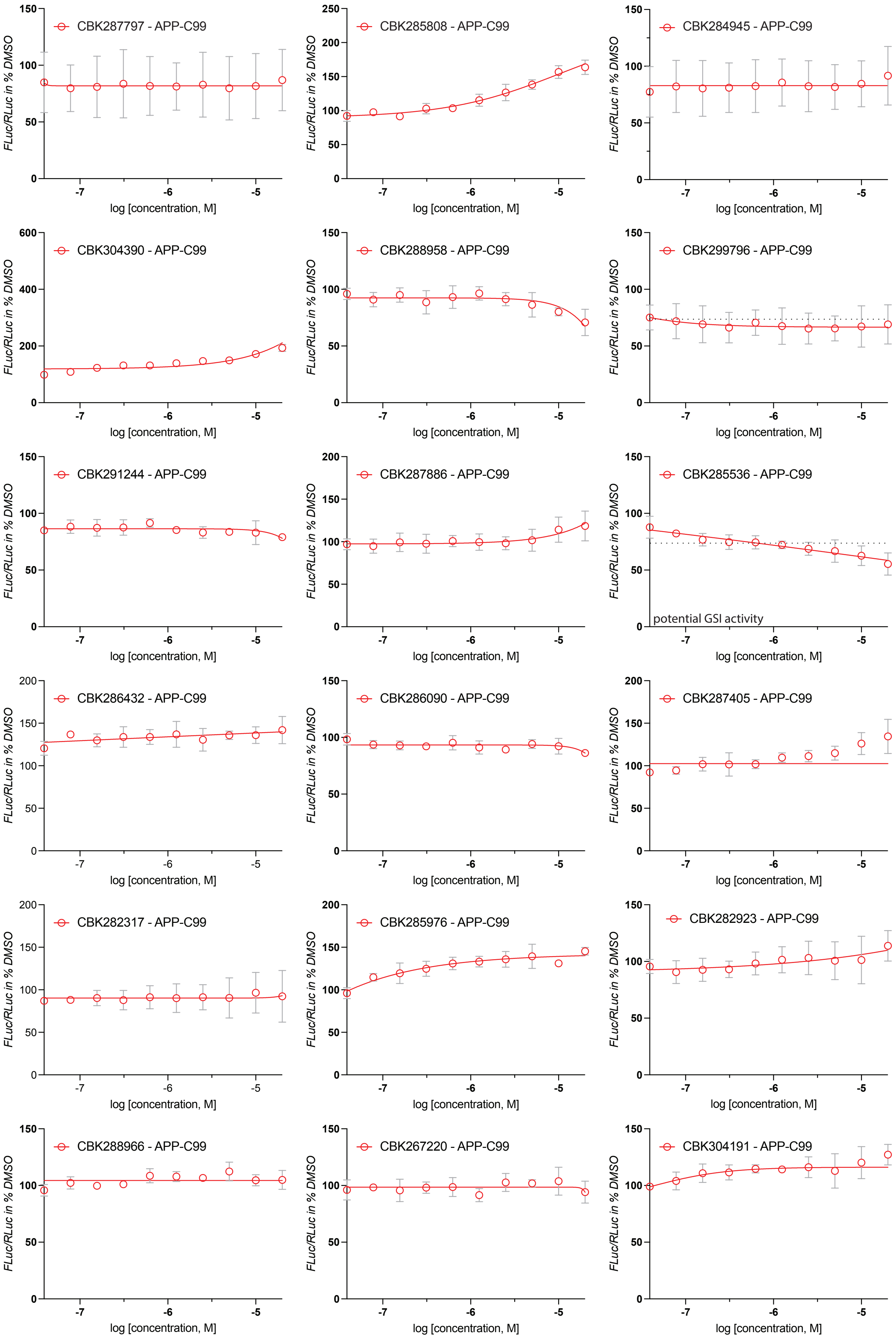

Supplemental Figure 3E continued

11-point dose-response curves: APP-C99 counter-assay

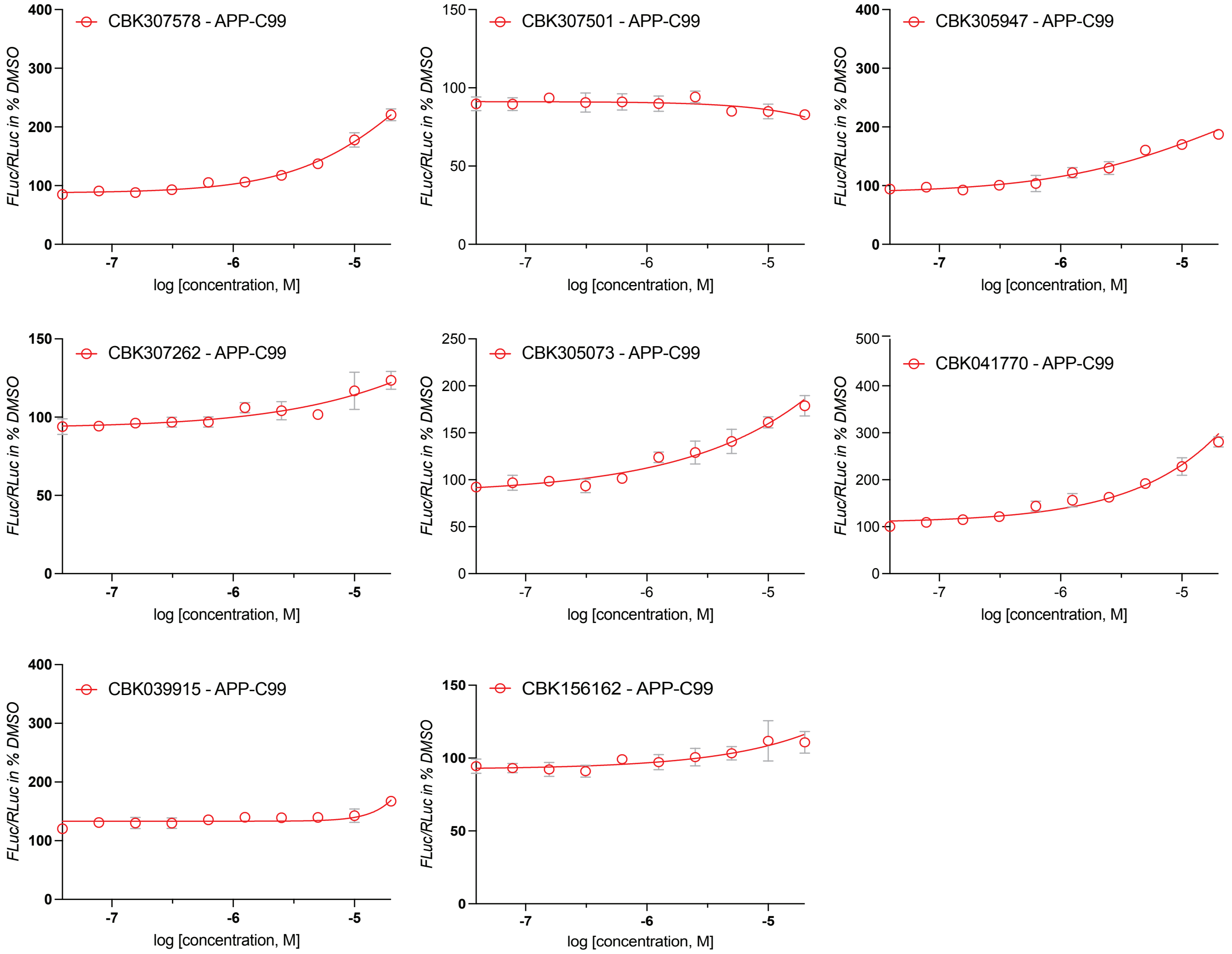

Supplemental Figure 3 continued

F

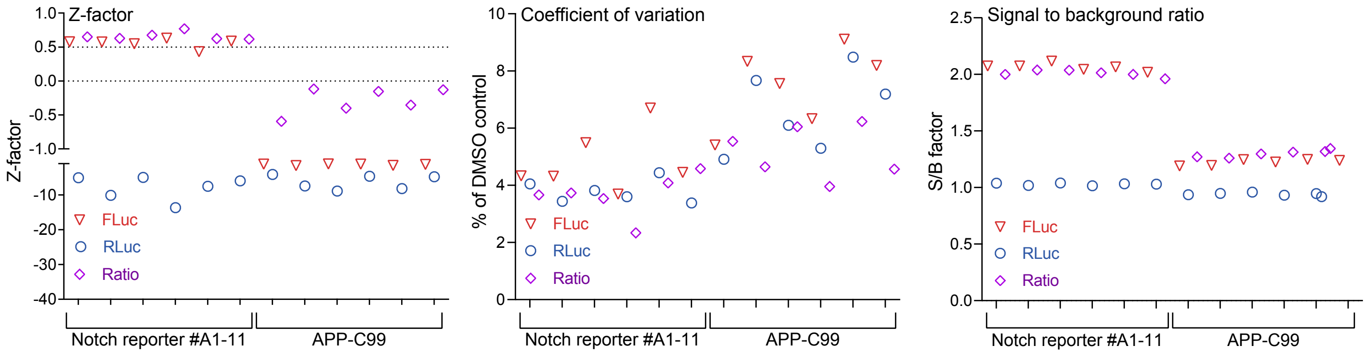

G

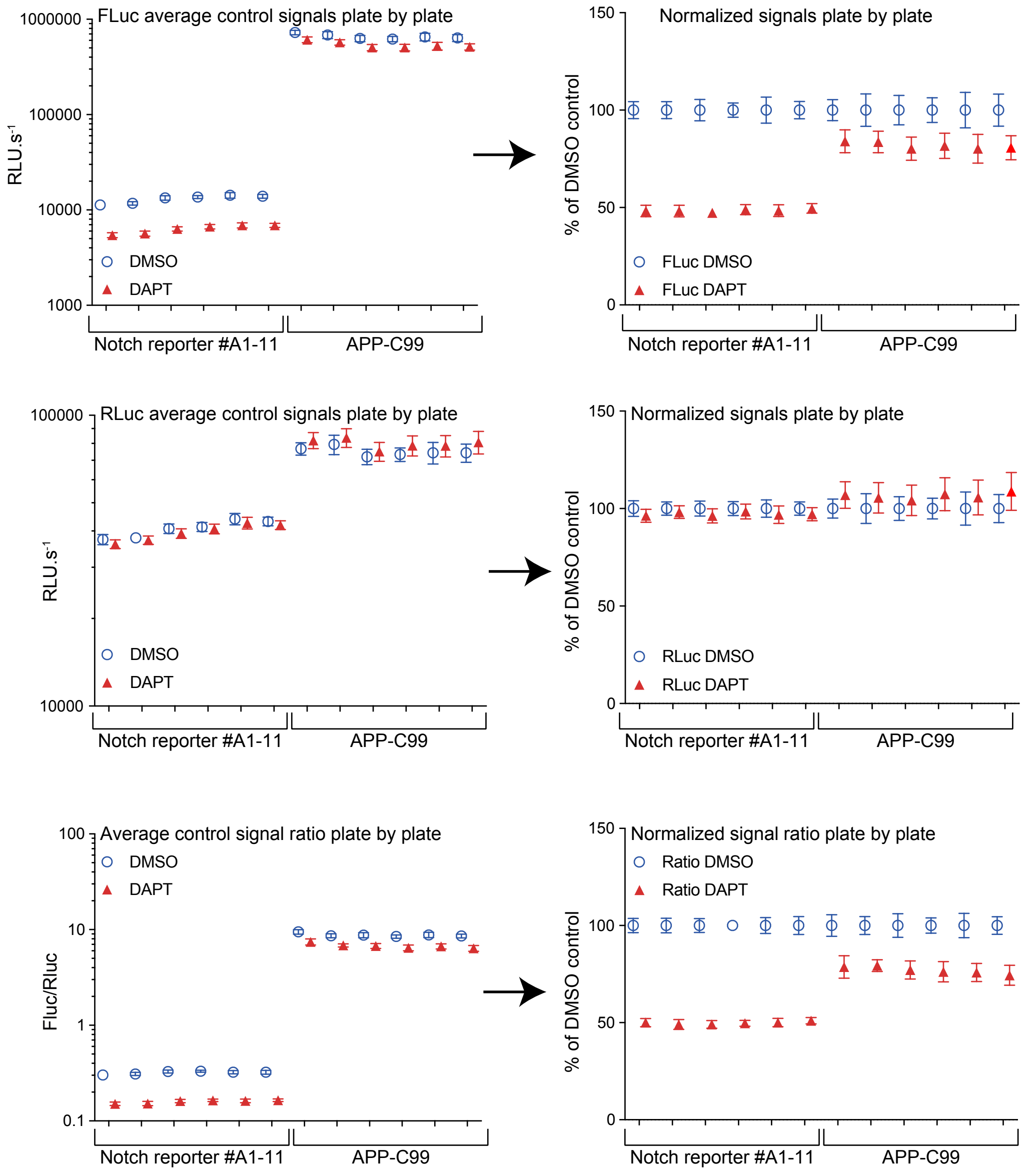

Supplemental Figure 3:

Validation of the APP-C99 counter assay. (A) Signal intensities for firefly (upper) and renilla (lower) luciferase signals across DMSO plate. Row numbers from A-P, column numbers from 1-22. At the bottom the firefly/renilla luciferase ratio is presented. The red dots indicate the signals of the Notch-reporter, that was used for comparison. **(B)** Analysis of three luciferase inhibitors (PTC-124, luciferase inhibitor 1 and Nano-Luc inhibitor) as well as FLI-06, LY-450139, LY-411575 and DAPT in the APP-C99 counter assay at three different dosages (1.25; 10 and 25 μM). **(C)** Analysis of data from the APP-C99 counter assay and the Notch confirmation assay to remove potential GSIs decreasing reporter signal to less than 70% in the counter assay at three different dosages of compound (1.25, 10 and 25 μM). The blue box marks compounds that met the criteria and were further investigated, while the red box presents compounds that failed to meet the thresholds and were sorted out. **(D)** Analysis of LY-450139, LY-411575 and DAPT in the APP-C99 screen at four different dosages (3.125; 6.25; 12.5 and 25 μM), and of luciferase inhibitor 1 and PTC-124 at 50 μM. **(E)** Dose-response curves from the Notch reporter assay (red) and the APP-C99 assay (blue) using compounds at 11 different doses. **(F)** Plate statistics for the 11 does-points APP-C99 and Notch reporter screens are shown, as indicated. **(G)** To the left, average firefly luciferase signals of DMSO and DAPT controls are shown. Averaged DMSO and DAPT ratio is shown to the right and below the respective averaged renilla luciferase signals are shown. The reporter signals of DMSO and DAPT after normalization are shown at the bottom, left and the DMSO/DAPT ratio relative to the DMSO controls is shown at the bottom, right.

Supplemental Figure 4

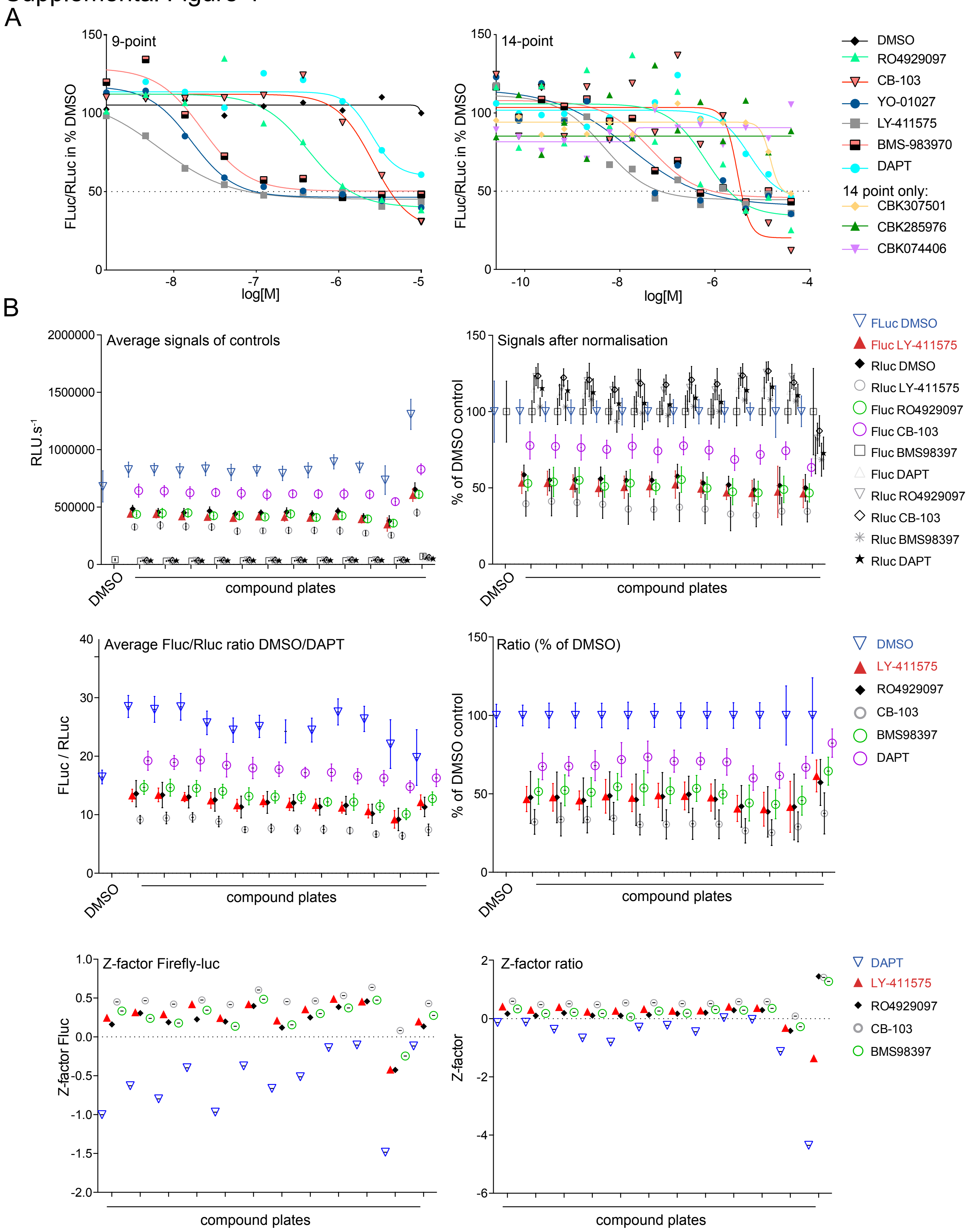

Supplemental Figure 4 continued

C

14-point dose-response curves: APP-C99 assay

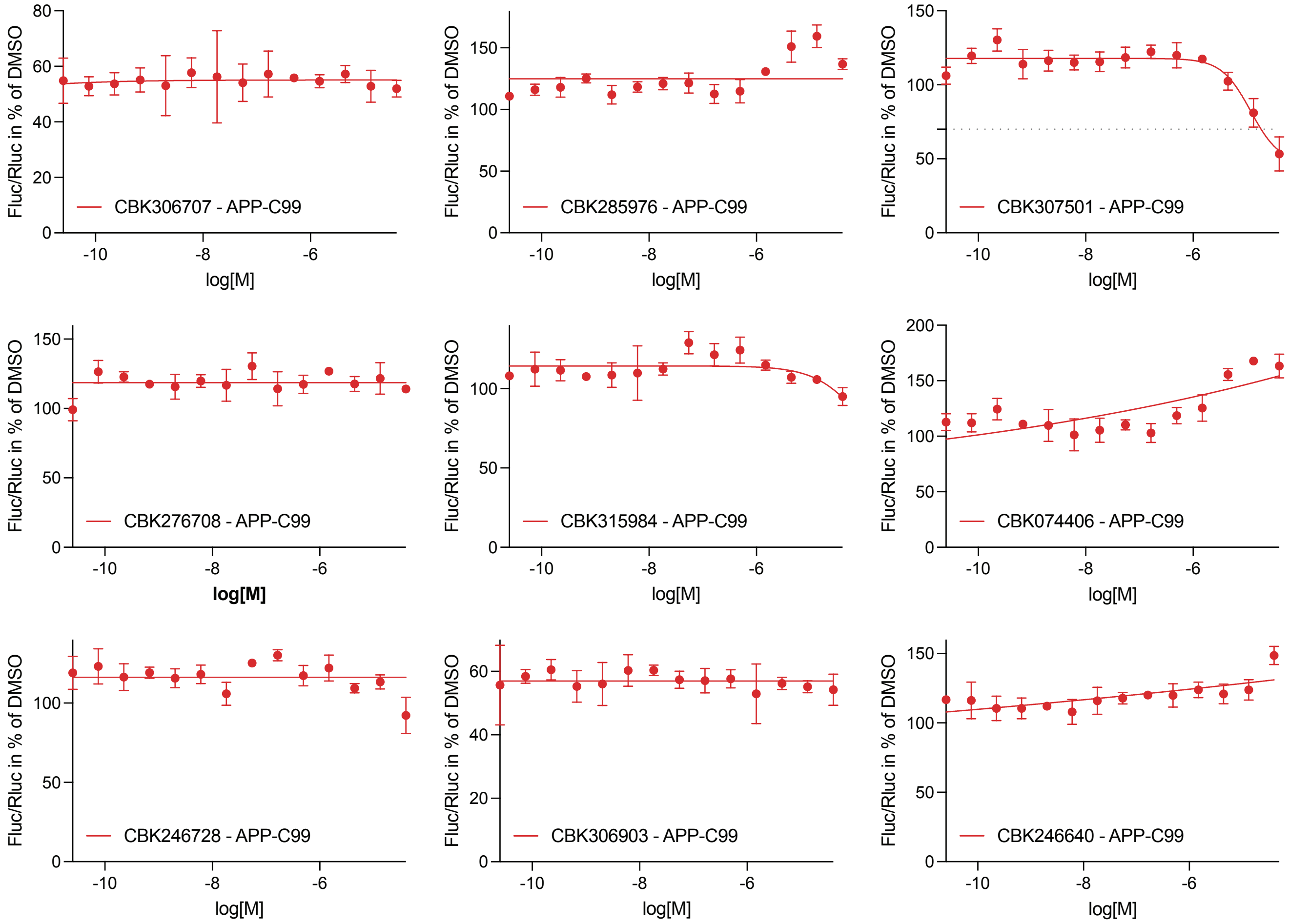

Supplemental Figure 4C continued

14-point dose-response curves: APP-C99 assay

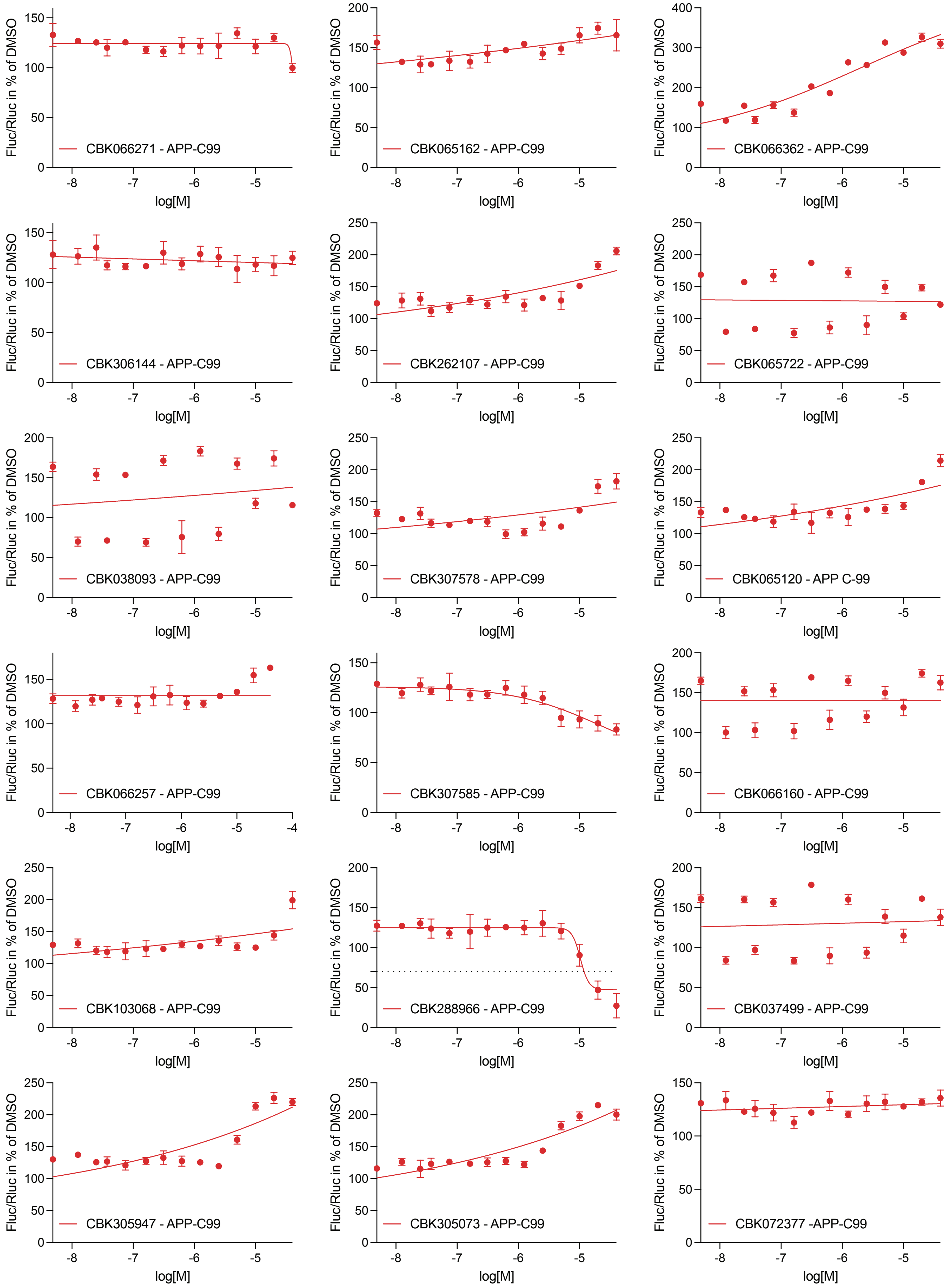

Supplemental Figure 4C continued

14-point dose-response curves: APP-C99 assay

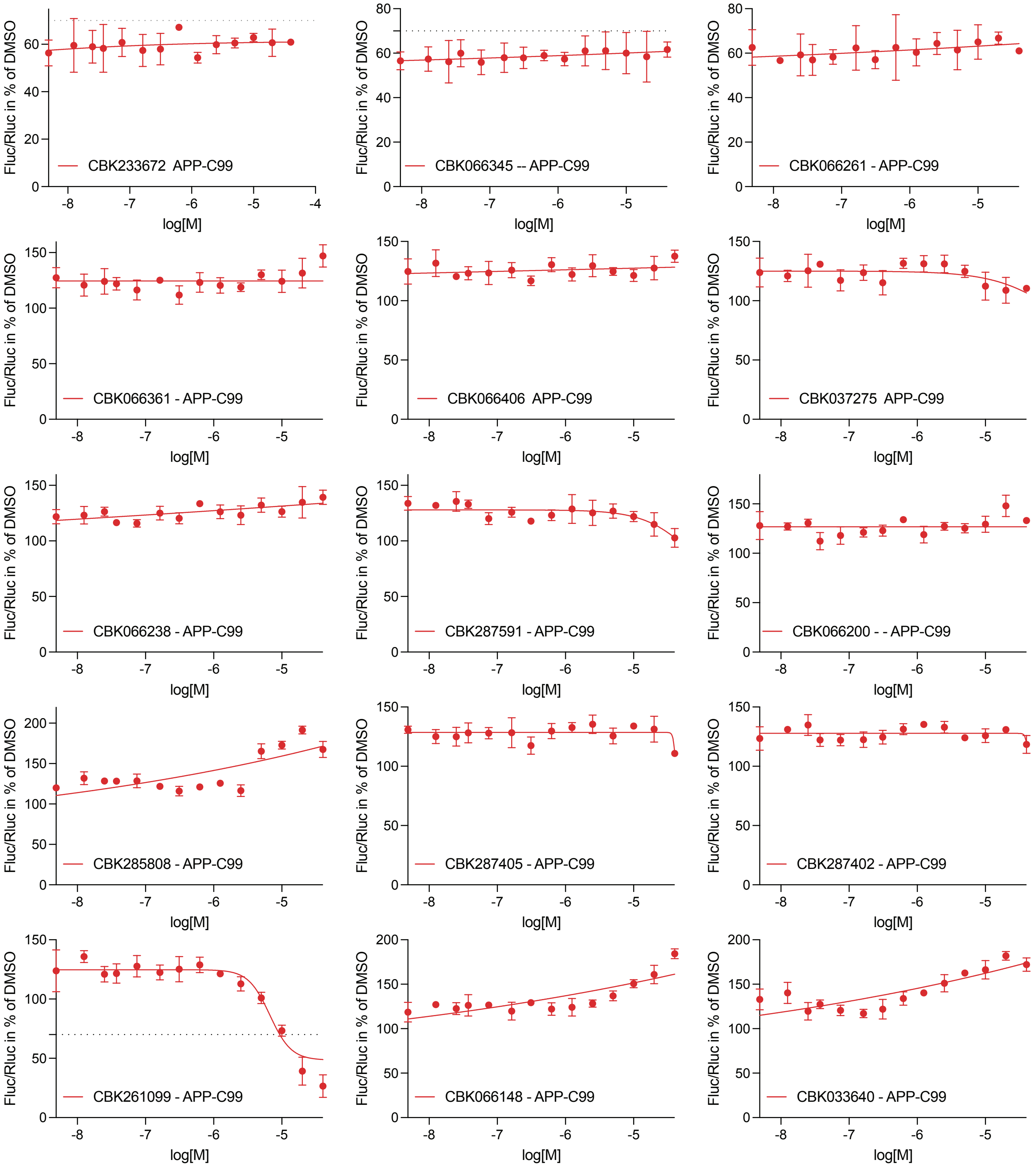

Supplemental Figure 4C continued

14-point dose-response curves: APP-C99 assay

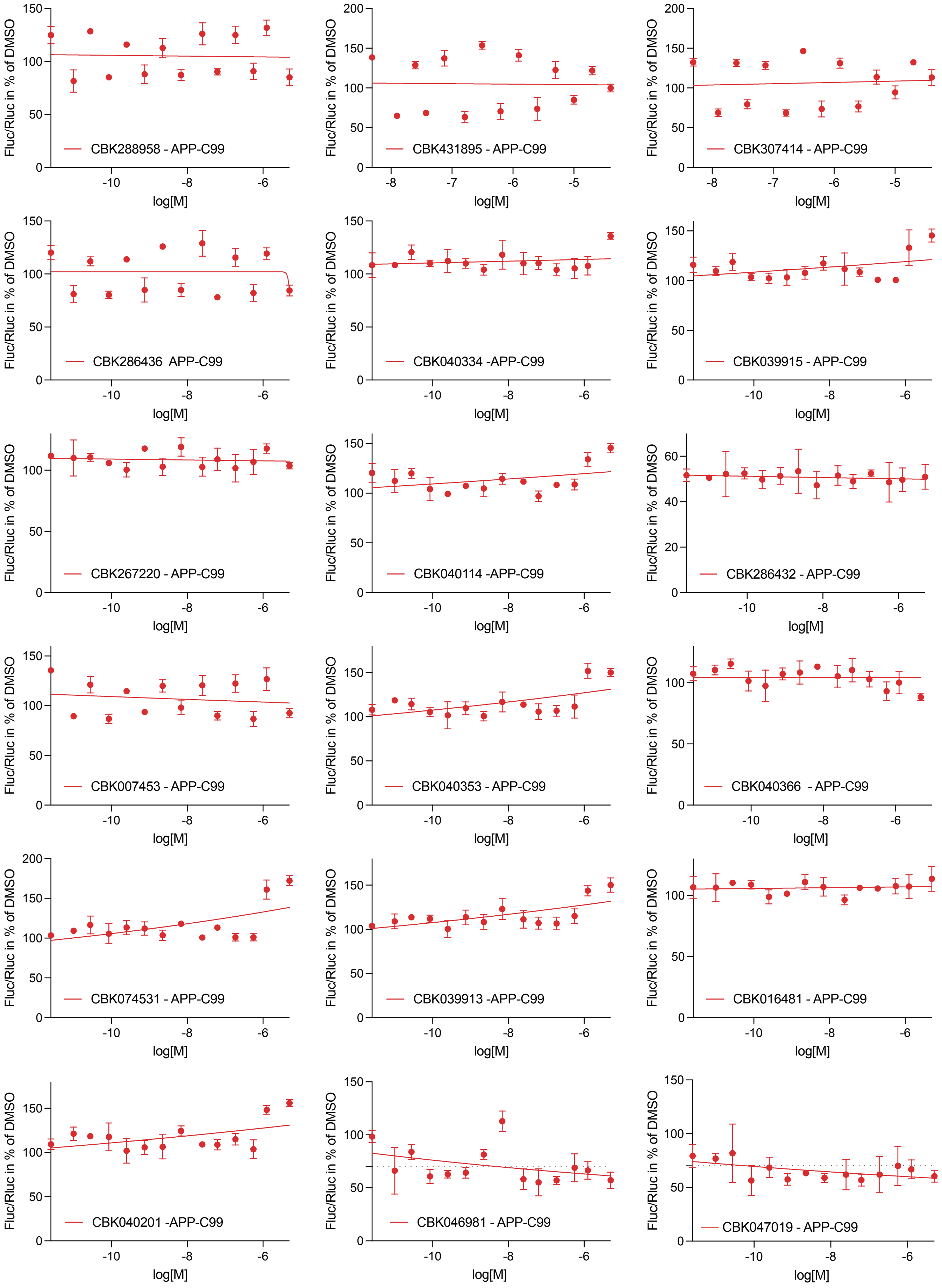

Supplemental Figure 4C continued

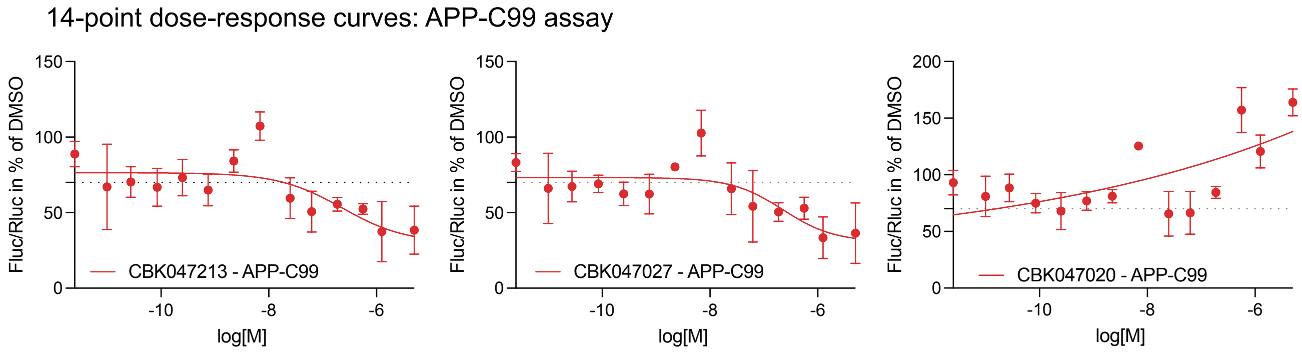

Supplemental Figure 4 continued

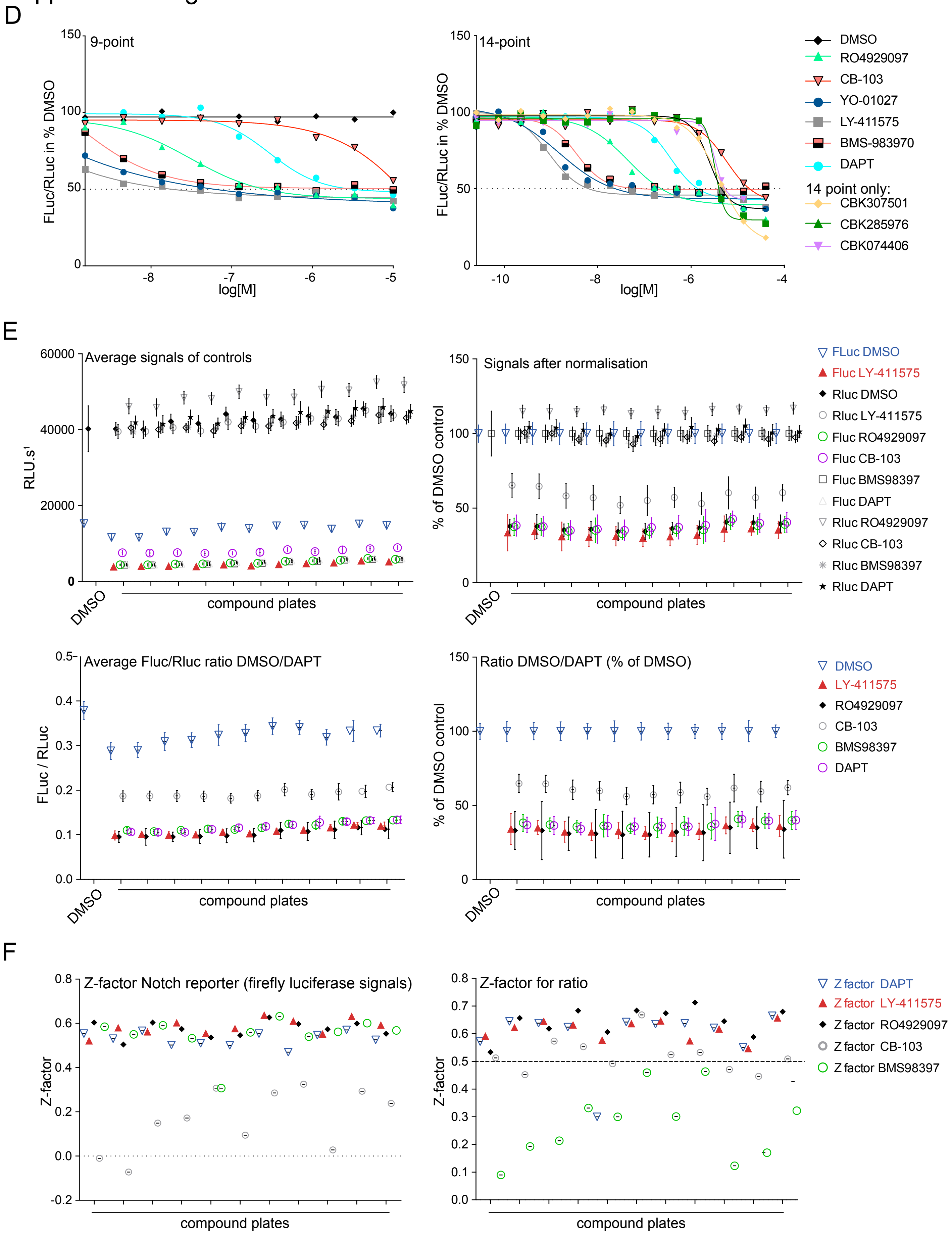

Supplemental Figure 4 continued
G

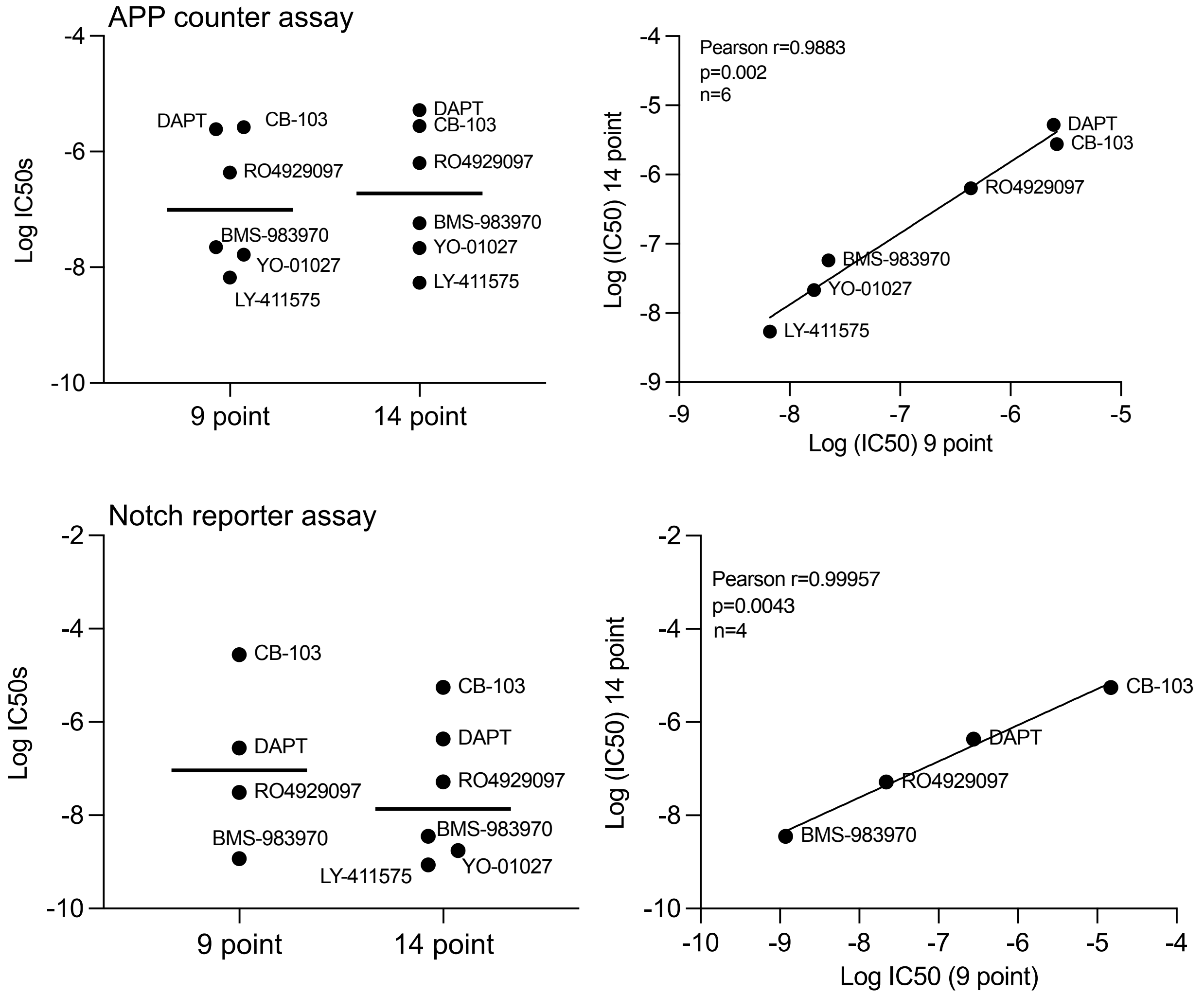

Supplemental Figure 4 continued

H

14-point dose-response curves: Notch reporter assay

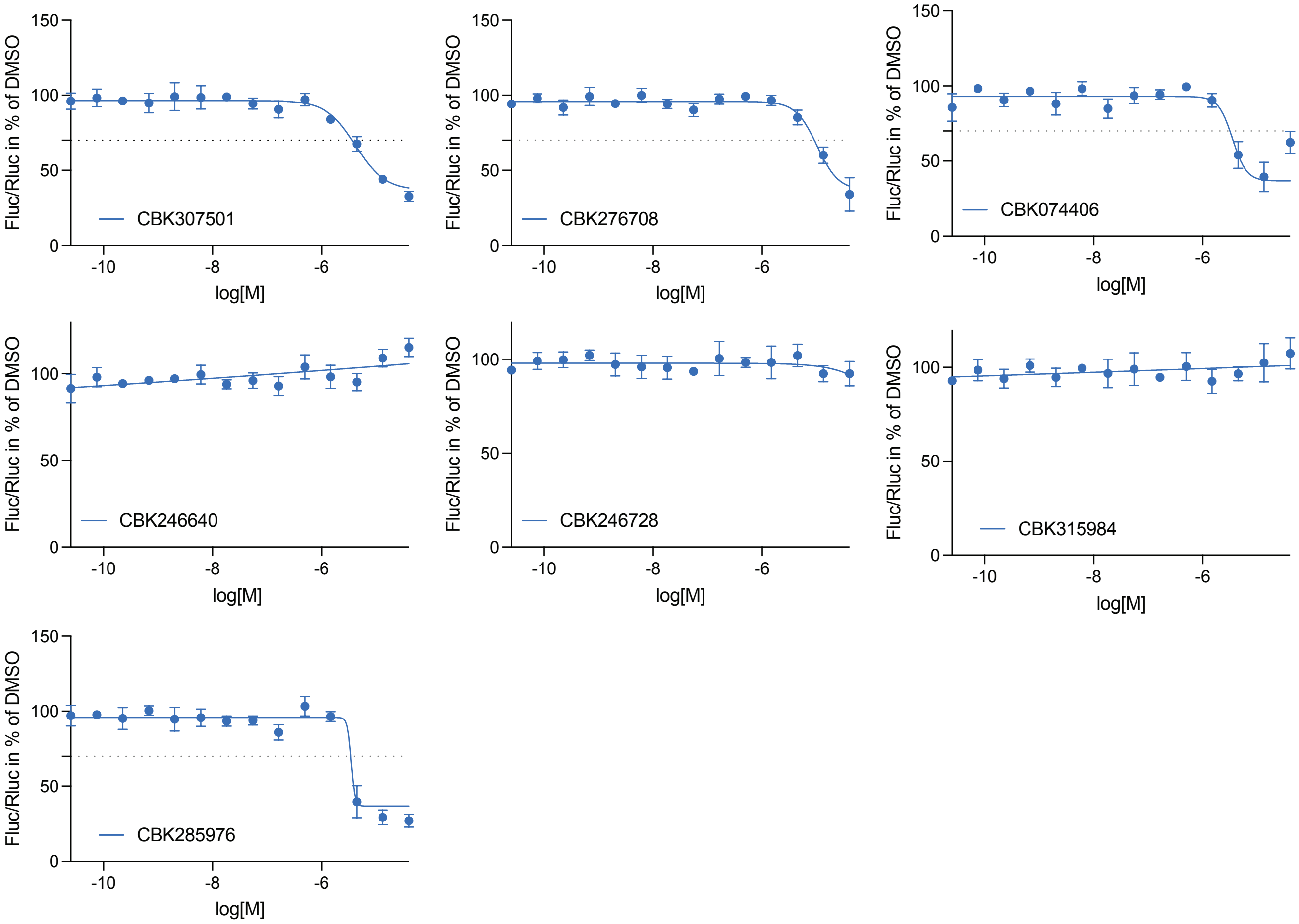

Supplemental Figure 4H continued

14-point dose-response curves: Notch reporter assay

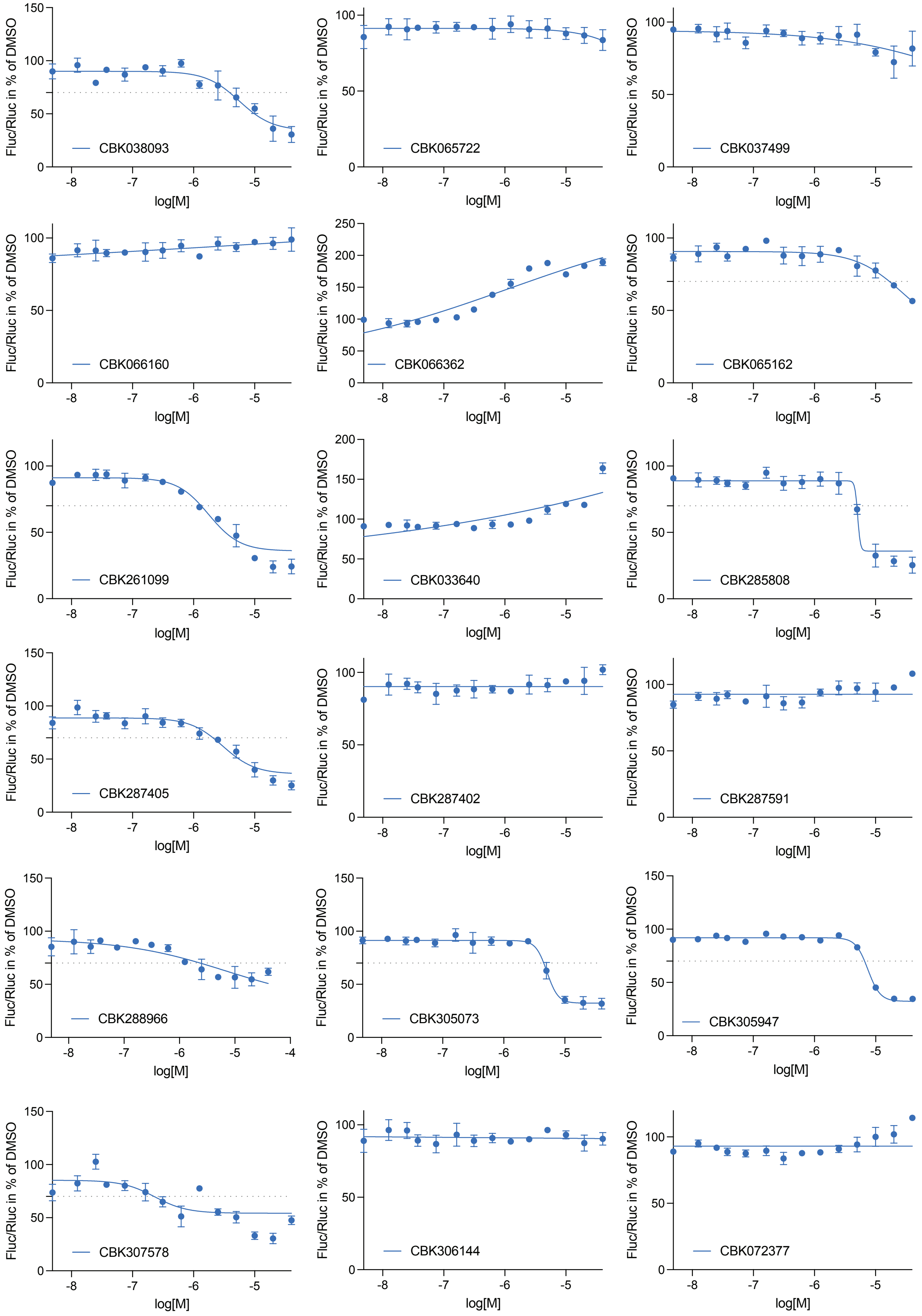

Supplemental Figure 4H continued

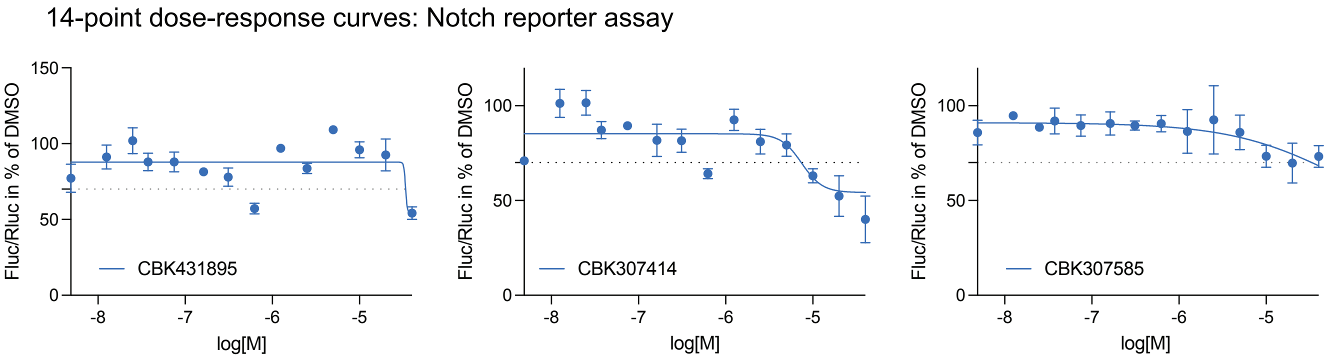

Supplemental Figure 4H continued

14-point dose-response curves: Notch reporter assay

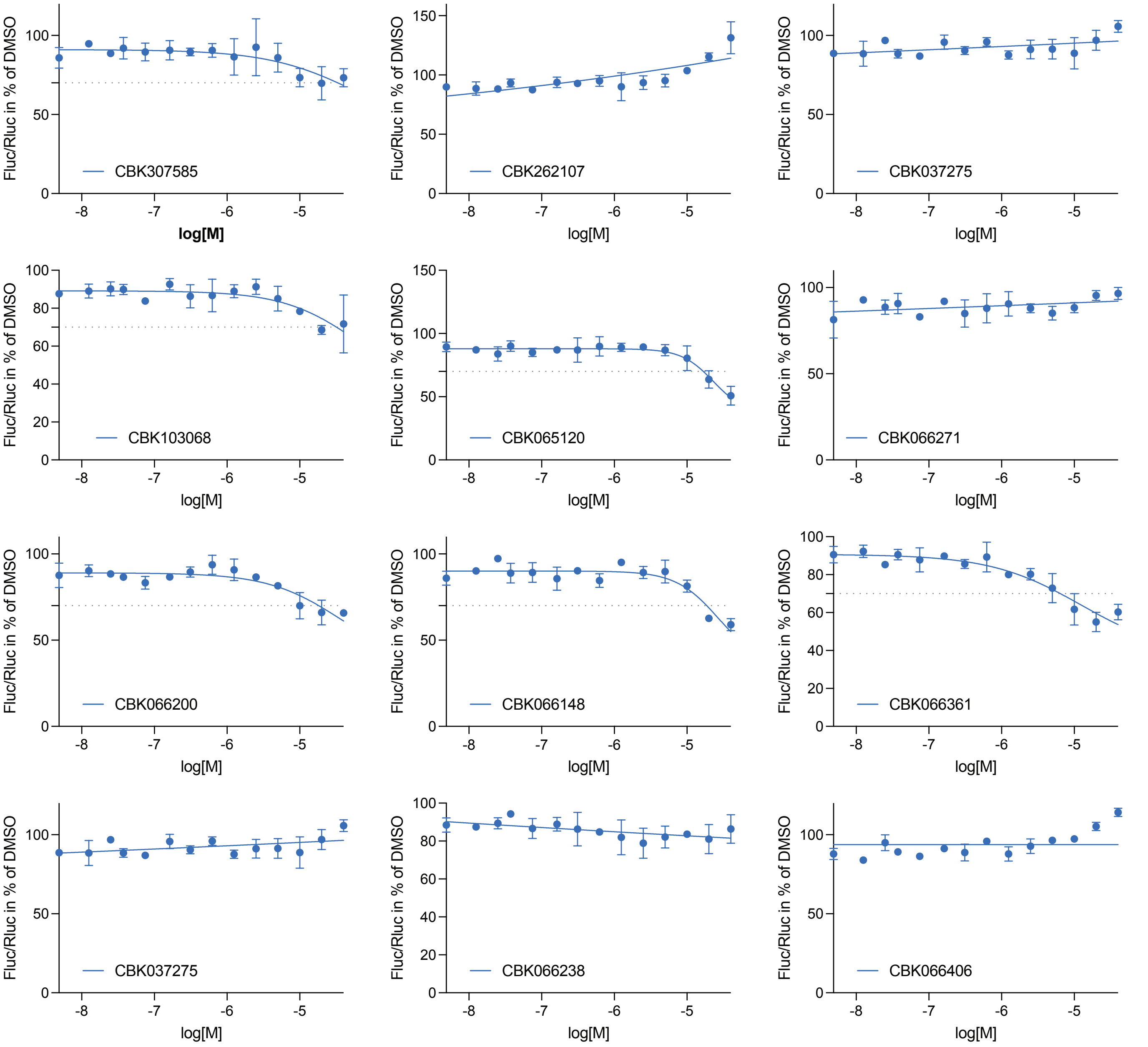

Supplemental Figure 4H continued

14-point dose-response curves: Notch reporter assay

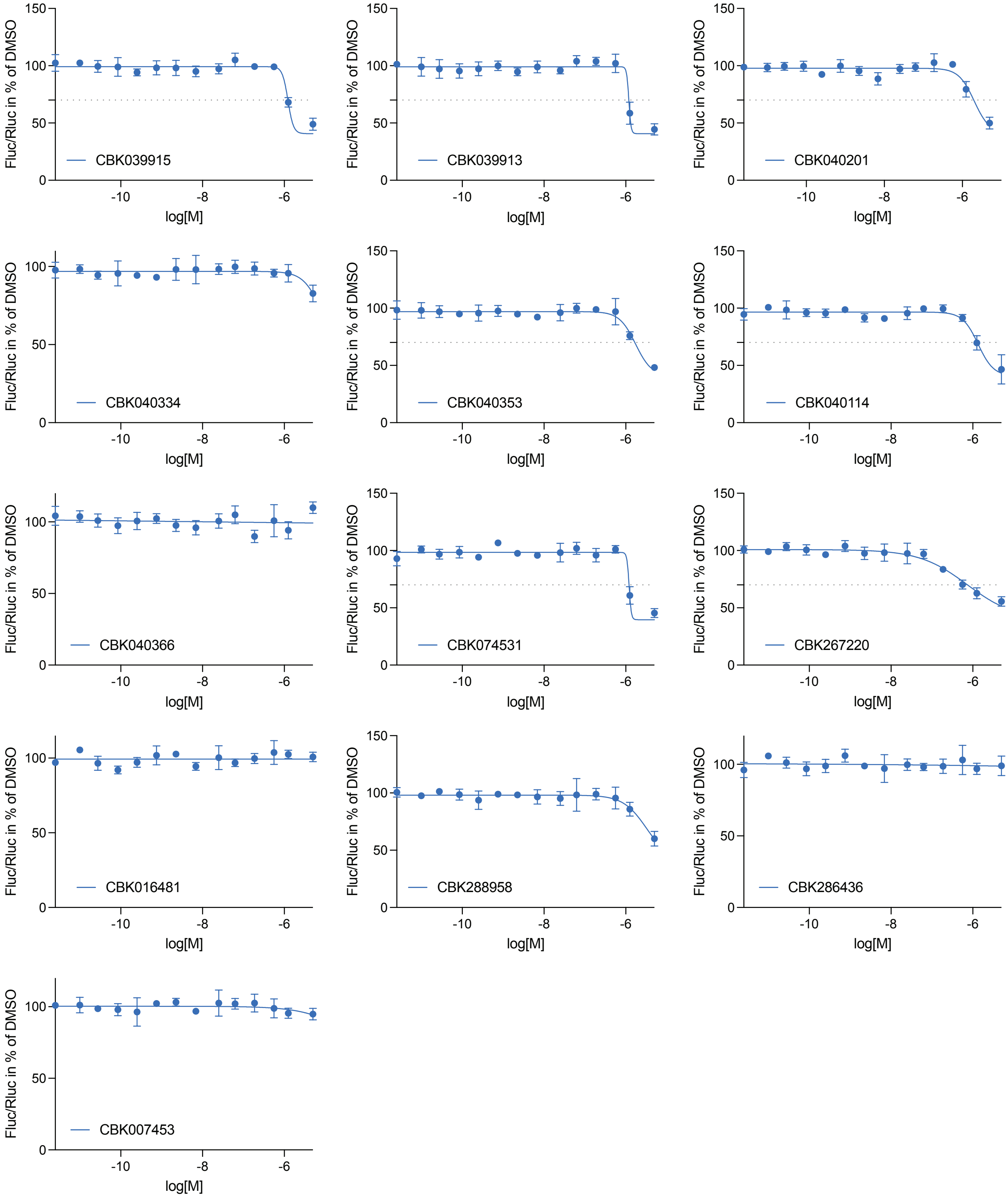

Supplemental Figure 4

I

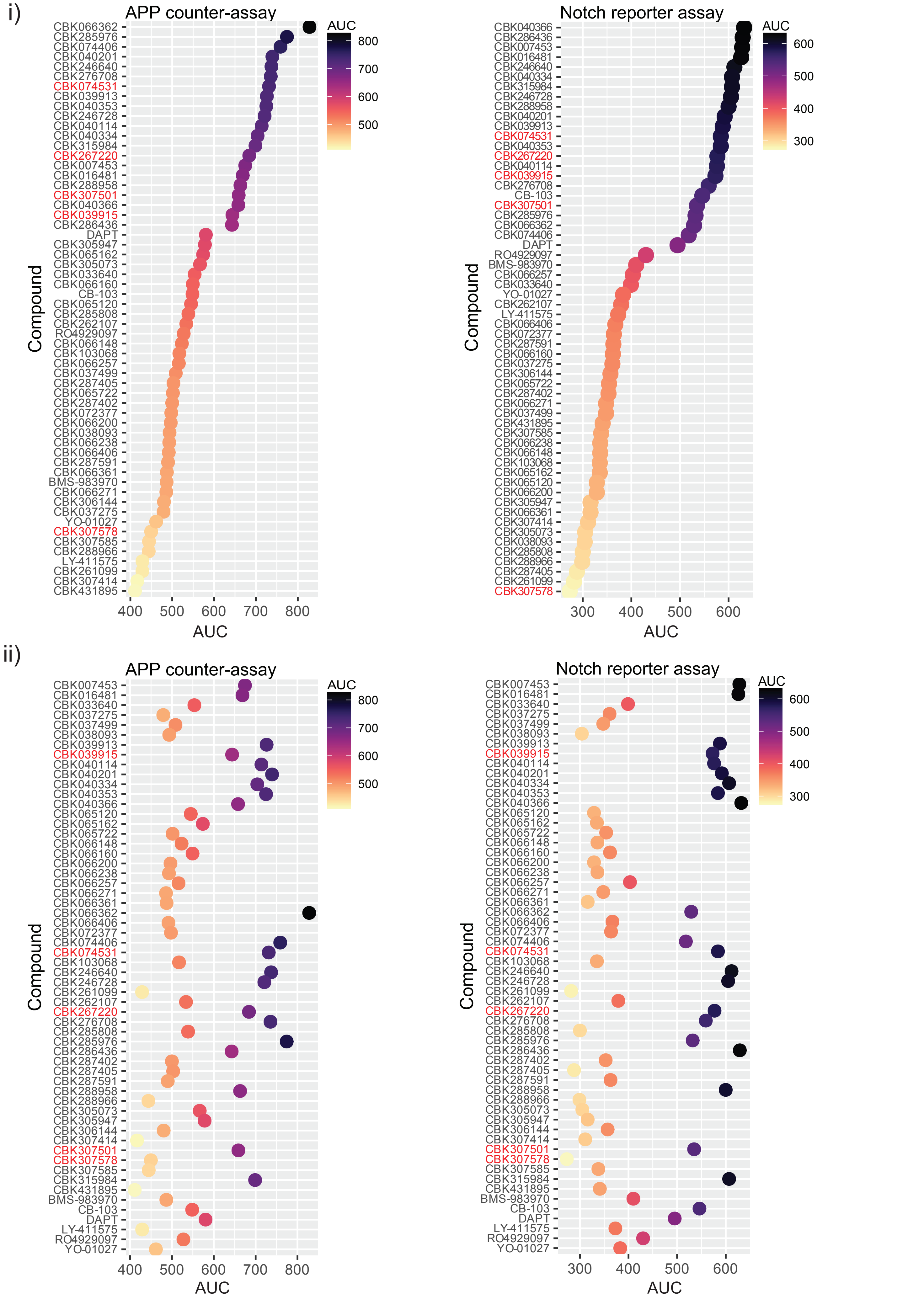

Supplemental Figure 4

J

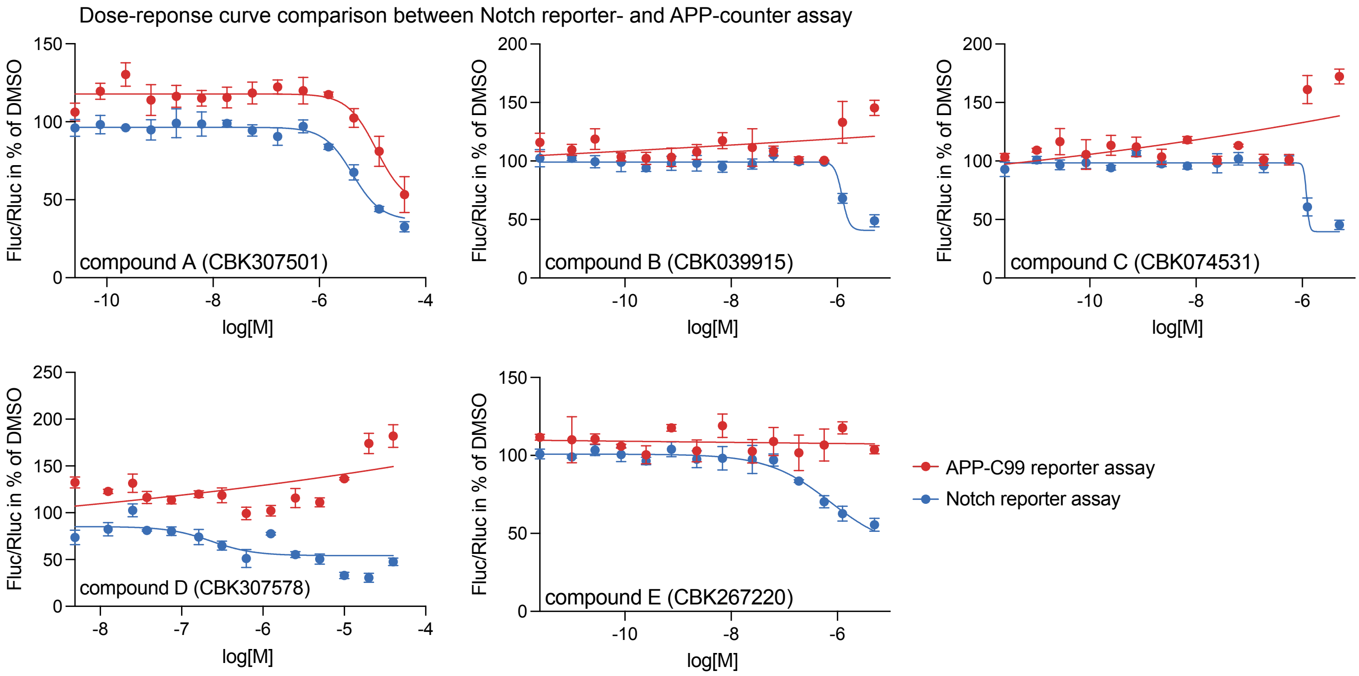

Supplemental Figure 4
K

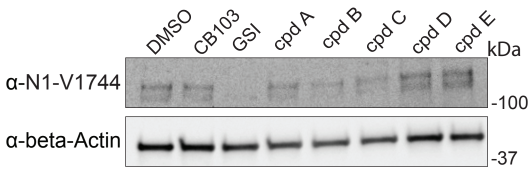

Supplemental Figure 4:

Validation of the APP-C99 counter assay in a 14-point dose-response regimen.

(A) Dose-response curves from the APP-C99 counter assay in a pilot 9-point and 14-point dose regimen for RO4929097, YO-01027, LY-411575, BMS-983970, DAPT and CB-103, as indicated. Black cell 384-well plates were also evaluated (data not shown). **(B)** Plate statistics for the APP-C99 counter assay in the 14-point dose regimen format. Average control signals, reporter signals after normalization, average Fluc/Rluc ratio in response to DAPT or DMSO, ratio of DMSO/DAPT and Z'-factor were established for the various compounds, as indicated. **(C)** Dose-response curves from the APP-C99 counter assay using compounds at 14 different concentrations. DMSO was not included as control in the 14-point assay, as it was included in the 9-point assay. **(D)** Dose-response curves from the Notch reporter assay in a pilot 9- and 14-point dose regimen for RO4929097, YO-01027, LY-411575, BMS-983970, DAPT and CB-103, as indicated. **(E)** Plate statistics for the Notch reporter assay in the 14-point dose regimen format. Average control signals, reporter signals after normalization, average Fluc/Rluc ratio in response to DAPT or DMSO, ration DMSO/DAPT and Z'-factor were established for the various compounds, as indicated. **(F)** Dose-response curves from the Notch reporter assay using compounds at 14 different concentrations. **(G)** 14-point dose response curves from the Notch reporter assays. The names of the compounds are shown in the figure. **(H)** Summary figure of the results from the APP counter and Notch reporter assay from the 9- and 14-point dose regimens. **(I)** Area under curve (AUC) results from the APP counter and Notch reporter assays presented in a waterfall format sorted by AUC value (i) or compound number (ii). Compounds A-E are highlighted in the figure. **(J)** Comparisons between dose response curves from the APP counter and Notch reporter assays for compound A-E. **(K)** Westen blot analysis of the NOTCH1 intracellular domain, analysed by the a-N1-V1744 antibody. a-beta-Actin was used as a loading control. Quantification of the western blot data are provided in Supplemental Table 4G.

Supplemental Figure 5

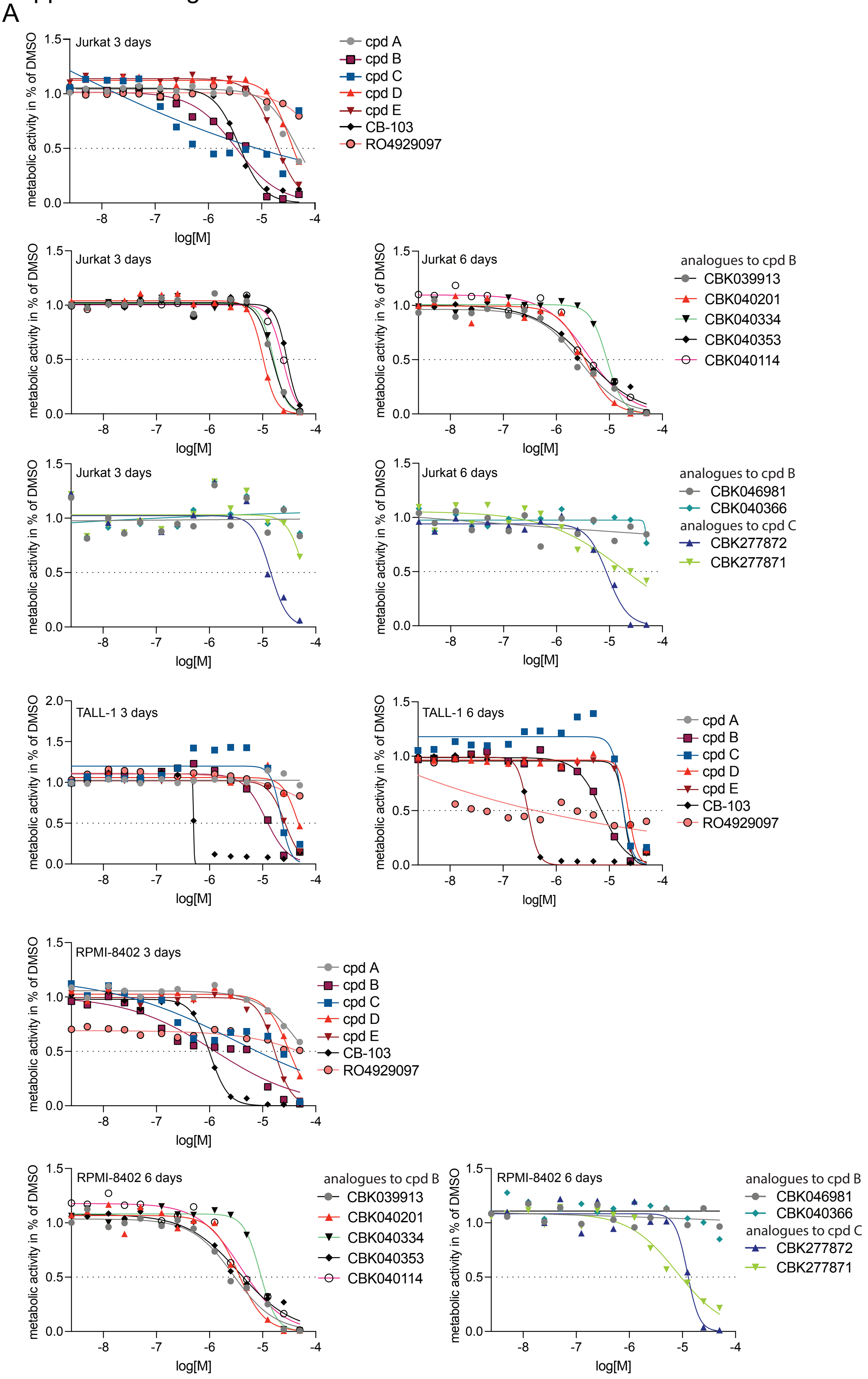

Supplemental Figure 5 continued

B

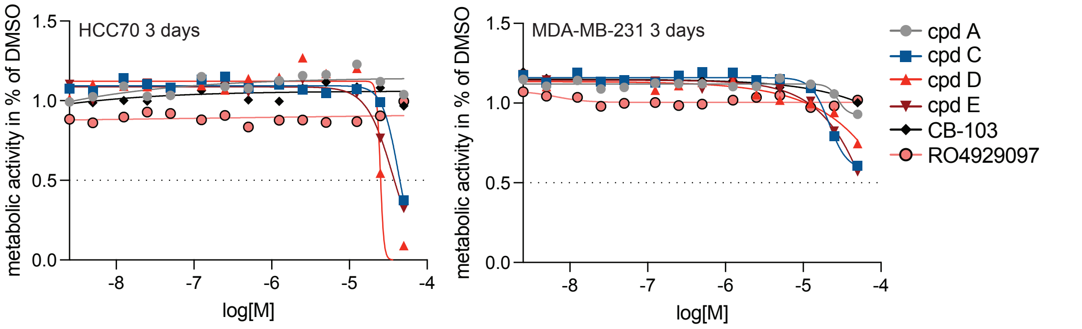

C

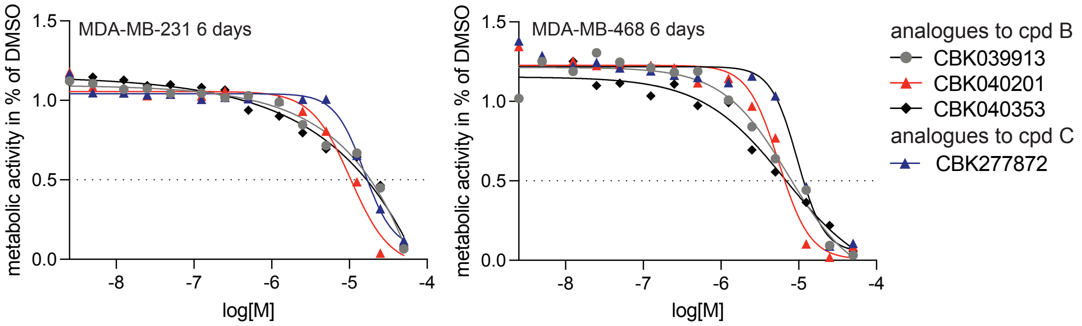

Supplemental Figure 5 continued

D

i)

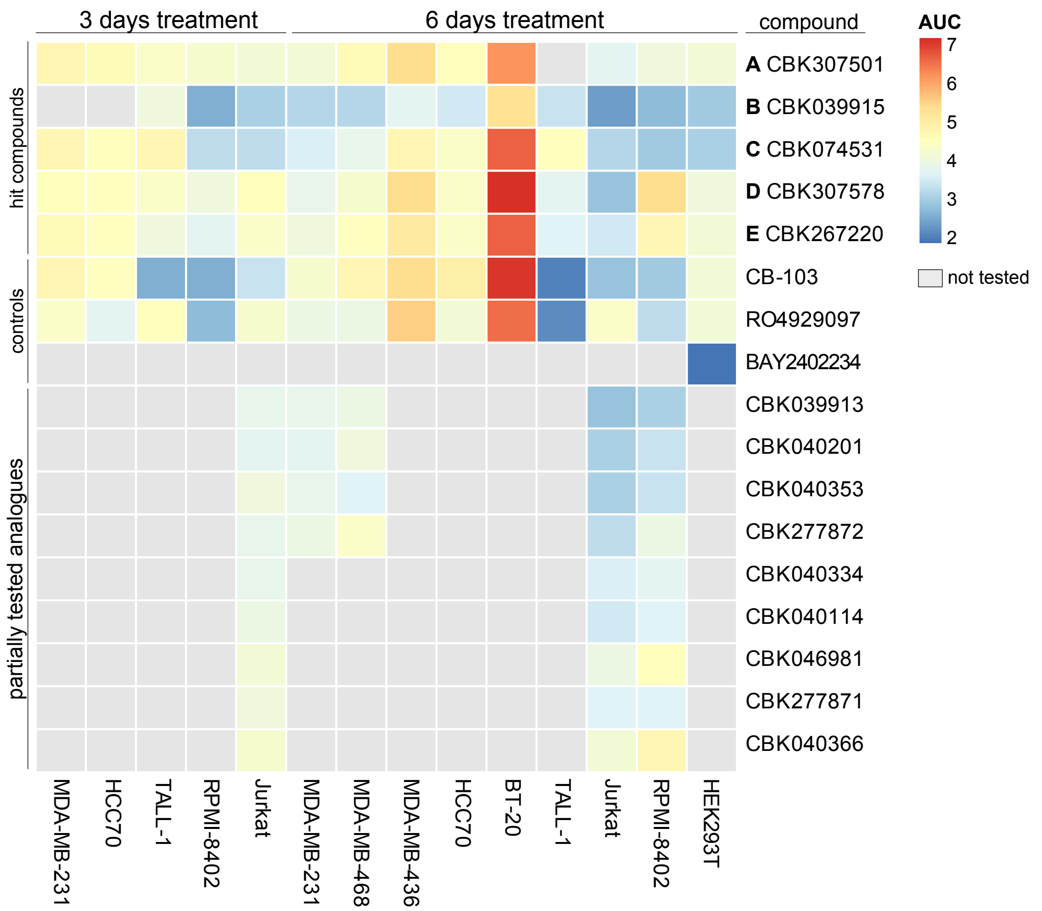

ii)

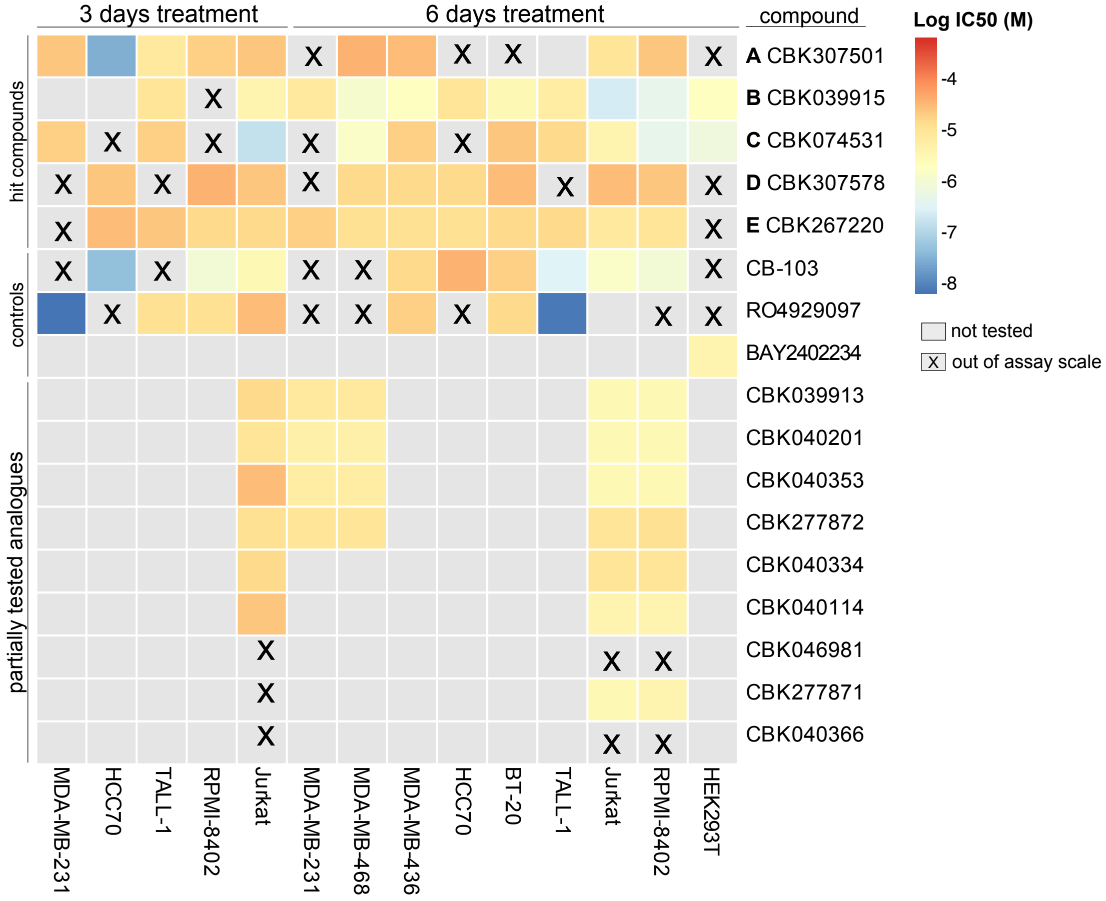

Supplemental Figure 5 continued

E

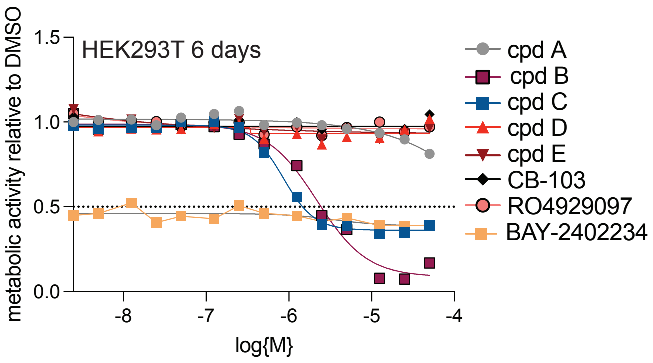

Supplemental Figure 5:

Analysis of compounds B and C and respective analogues for growth-inhibitory effects on T-ALL and breast cancer cell lines. Graphs showing 14-point growth-response curves for the level of metabolic activity in three T-ALL cell lines (JURKAT, TALL-1 and RPMI-8402) at three and six days after treatment with compound B, C and respective analogues, and CB-103 and RO4929097 as controls, as indicated. **(B)** Graphs showing 14-point growth-response curves for the level of metabolic activity in two breast cancer cell lines (MDA-MB-231 and HCC70) three days after treatment. **(C)** Graphs showing 14-point growth-response curves for the level of metabolic activity in two breast cancer cell lines (MDA-MB-231 and MDA-MB-468 after six days of treatment in different cell lines with analogous to compound B and C, as indicated. **(D)** A summary of the results from three- and six-days treatment with the various compounds, presented in a heatmap format. **(E)** Graph showing 14-point growth-response curves for the level of metabolic activity in HEK293T cells after six days treatment with compound A-E, CB-103, RO4929097 and BAY2402234.

Supplemental Figure 6

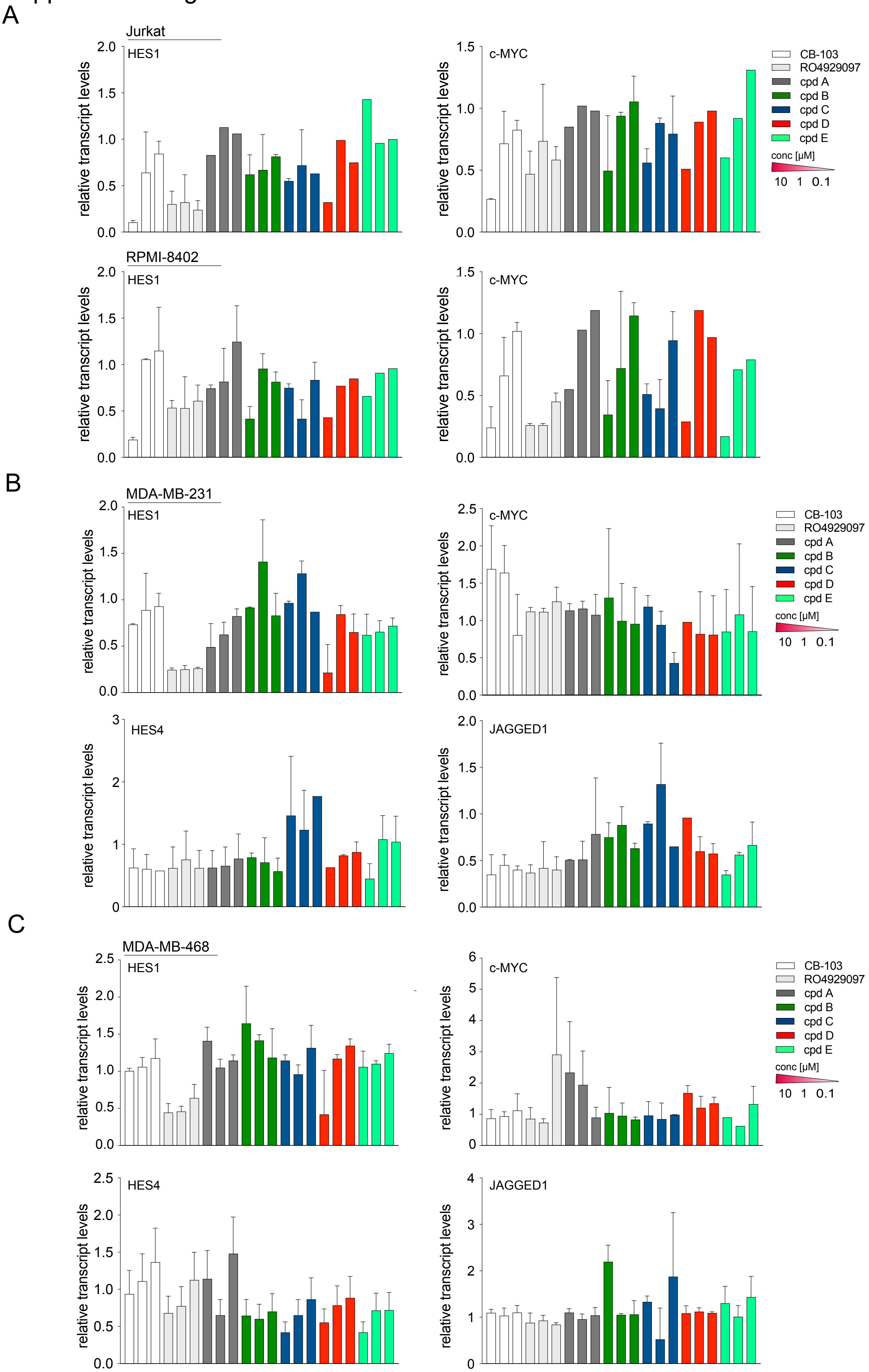

Plots with error bars represent mean ± SD of two biological replicates. Bars without error bars indicate that only technical replicates (n=1) were available

Supplemental Figure 6:

Analysis of Notch downstream gene expression in response to compounds A-E. (A) Analysis of mRNA expression levels of Notch downstream genes analysed by qPCR, as indicated. Values in response to three different doses (0.1; 1 and 10 μM) of compound A-E, and as controls CB-103 and RO4929097, in JURKAT (upper) and RPMI-8402 (lower) cell lines are shown. Cells were incubated for 24 hours with each compound. **(B, C)** Corresponding analysis as in **(A)** for MDA-MB-231 **(B)**, MDA-MB-468 **(C)** cell lines are shown. Plots with error bars represent mean ± SD of two biological replicates. Bars without error bars indicate that only technical replicates (n=1) were available.

Supplemental Figure 7

A

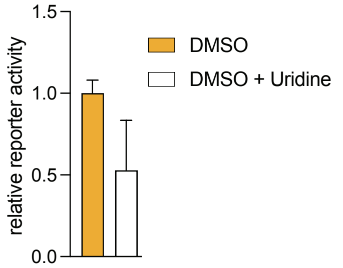

B

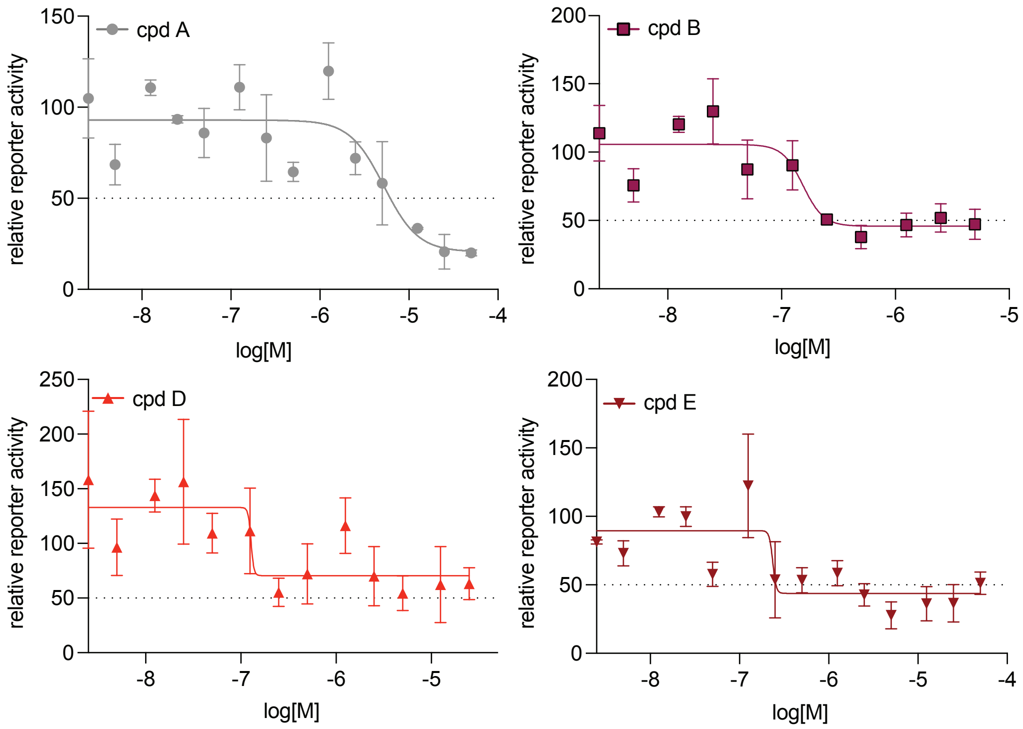

Supplemental Figure 7:

Assessing hit compounds in expanded dose range. **(A)** Relative Notch reporter activity in response to DMSO or DMSO supplemented with 100 μM uridine. **(B)** Compound A, B, D and E were assessed in the Notch reporter assay at extended dose range. High dose-points associated with cytotoxicity were removed for compound B at concentrations higher than 5 μM, and for C and D at concentrations higher than 12.5 μM to improve robustness of curve fits.

Supplemental Figure 8

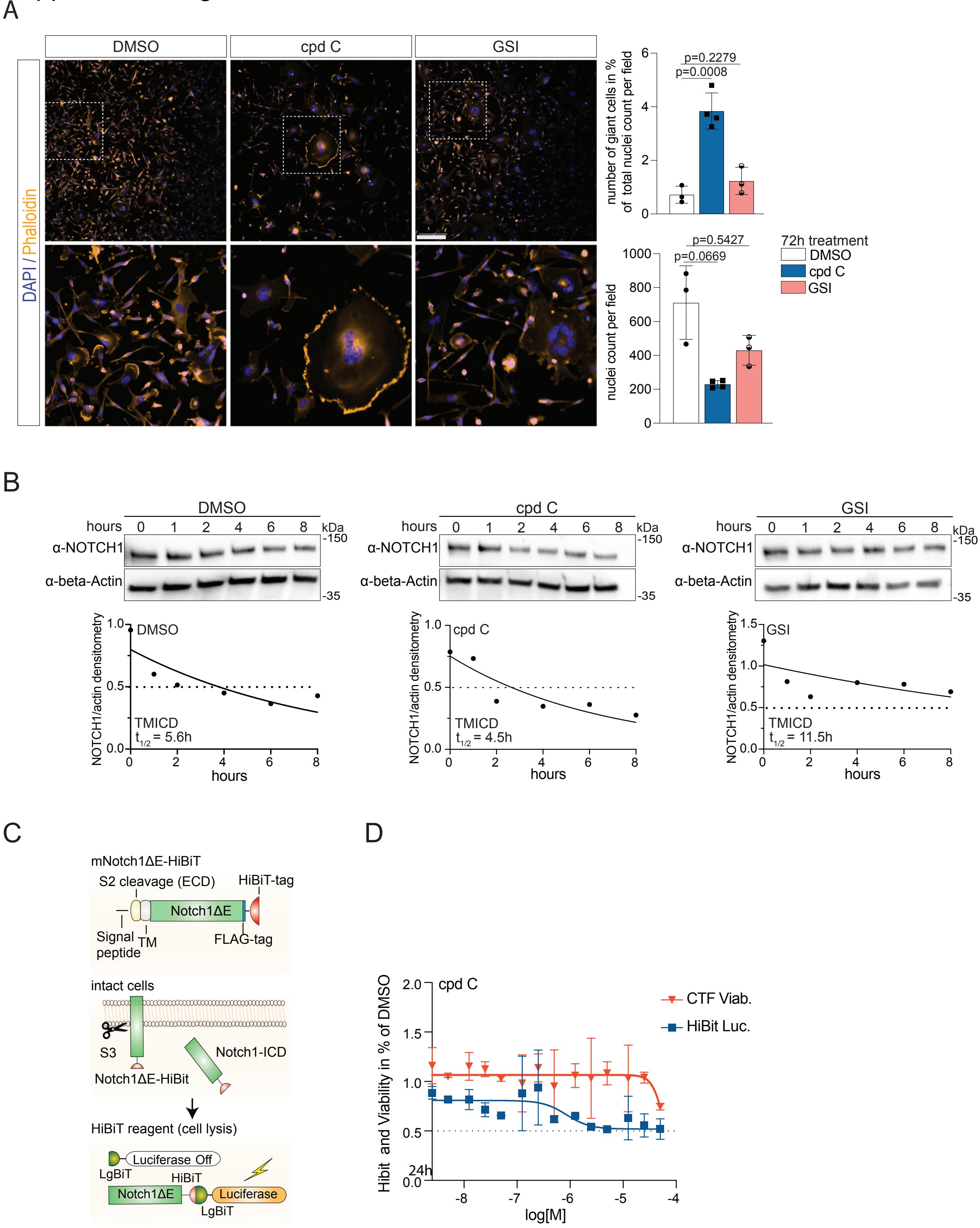

Supplemental Figure 8 continued

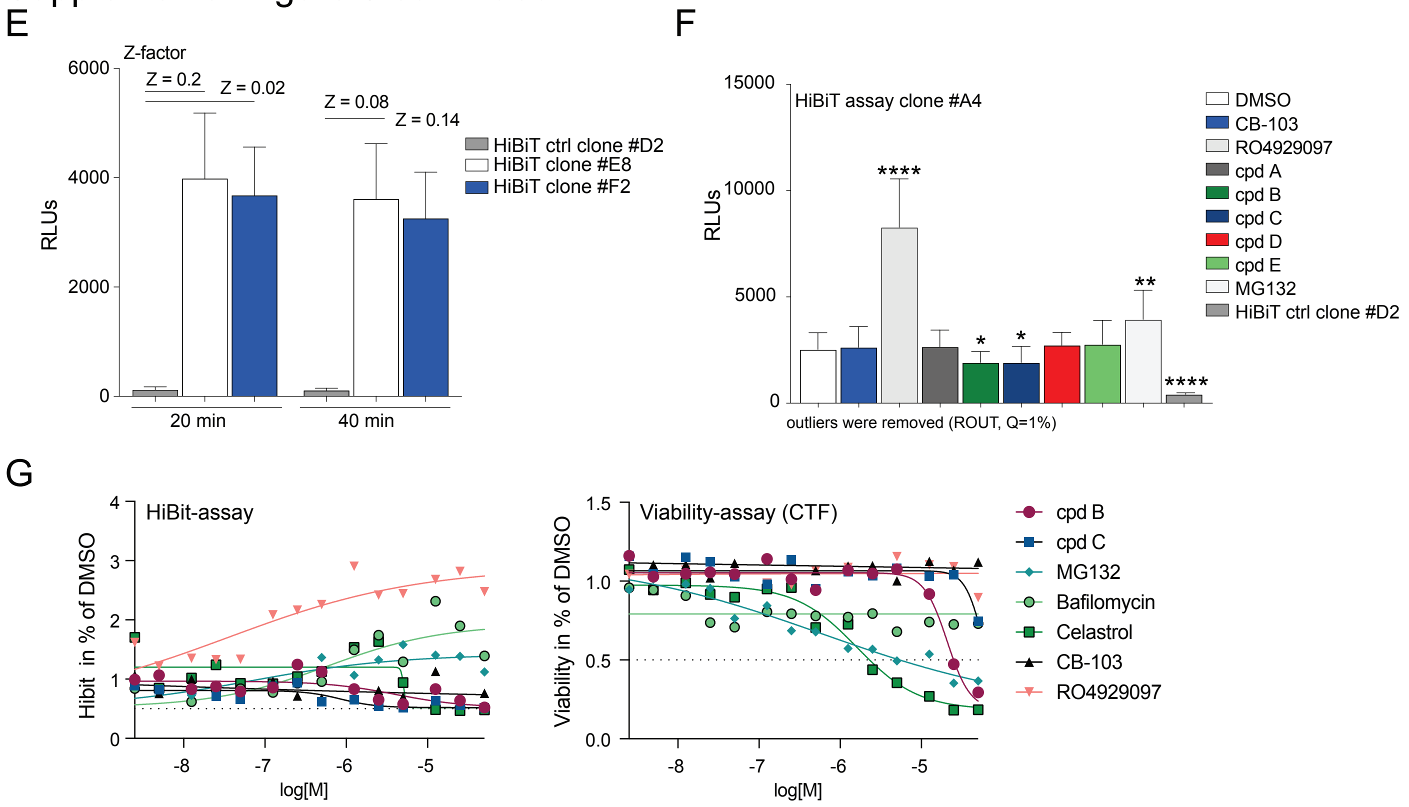

Supplemental Figure 8:

Analysis of Notch stability in response to compound C. (A) Immunocytochemistry (ICC) analysis of NOTCH1, phalloidin and DAPI staining in MDA-MD-231 cells cultured for 72 hours in the presence of 2 µM compound C (cpd C), 2 µM GSI (RO4929097) or DMSO, as control ol. To the right, quantification of the number of giant cells in % of total nuclei count per field (upper) and the nuclei count per field (lower) are shown, based on three (DMSO, GSI) and four (compound C) images. Two-tailed student's *t*-tests was performed (Welch's test) with DMSO as reference to assess statistical significance. The dotted area in the upper panel is enlarged in the panel below. Scale bar: 200 µm. **(B)** Analysis of cleaved NOTCH1 longevity by Western blot in the HEK293T Notch reporter cell line (#A1-11) 0-8 hours after addition of cycloheximide (CHX) to block translation. The effects of DMSO and 5 µM compound C and GSI (RO4929097) are shown. A pan-NOTCH1 antibody was used to visualise NOTCH1 bands and beta-actin staining was included as loading control. The membrane was first stained for anti-NOTCH1 and subsequently stripped and reprobed with a beta-actin antibody, as control. Samples treated with DMSO and compound C were loaded on the same gel, GSI (RO4929097)-treated samples were separated on a different gel. Graphs at the bottom show calculation of half-lives for cleaved NOTCH1 after CHX treatment from Western blot. The half-life reduction is expressed relative to NOTCH1 in DMSO control. **(C)** Schematic depiction of the HiBiT assay. The Notch1DE-HiBiT construct is depicted on top, and the cleavage by g-secretase is shown in the middle panel. At the bottom, the interaction with the LgBiT moiety, generating luciferase activity, is shown. **(D)** Graphs showing 14-point growth-response curves for the level of HiBiT luciferase activity in the HiBiT reporter cell line of two averaged experiments 24h after treatment with compound B and C at dose-ranges from 50 mM to 25 nM. In addition, dose-response curves for cell viability (labelled CTF Viab; using the CellTiter-Fluor Viability assay, CTF) are shown. **(E)** Luciferase activity in the HiBiT assay for two different HiBiT clones (#E8 and #F2) and HiBiT control clone #D2 (empty vector) 20 minutes (upper) or 40 minutes (lower) after lysis. The Z-factors for control cell line #D2 and HiBit clones #E8 and #F2 are also presented. **(F)** Analysis of luciferase activity from subclone #A4 in response to compounds A-E, CB-103, RO4929097 and MG132 for 24h at 10 mM concentration. The HiBiT control clone #D2 was included as control. Outliers were removed using ROUT (Q=1%), as indicated. **(G)** Graphs showing 14-point dose-response curves for the level of HiBiT luciferase activity in the HiBiT reporter cell line of two averaged experiments 24h after treatment with compound B and C at dose-ranges from 50 mM to 25 nM. In addition, dose-response curves for cell viability (labelled CTF Viab; using the CellTiter-Fluor Viability assay, CTF) are shown. RO4929097 and CB-103 were used as Notch inhibitor controls, Bafilomycin and MG132 were used to block protein degradation and Celastrol was used as a control to induce apoptosis. Data for compounds B and C are also part of Figure 8D.

Supplemental Figure 9

compound	Notch assay	APP assay	myogenic diff.	viability assay	DHODH assay	cell morphology	Notch targets
cpd C	nanomolar potency	inactive	yes	context specific	active	strongest effect	condition dependent
LY-411575	highest potency	highest potency	n.d.	not determined	n.d.	n.d.	n.d.
DAPT	comparable	higher potency	n.d.	context specific	n.d.	n.d.	n.d.
RO4929047	higher potency	higher potency	yes	context specific	n.d.	moderate effect	condition dependent
YO-01027	higher potency	higher potency	n.d.	n.d.	n.d.	n.d.	n.d.
CB-103	lower potency	weak potency	no	context specific	n.d.	n.d.	condition dependent
BMS-983970	higher potency	higher potency	n.d.	n.d.	n.d.	n.d.	n.d.
BAY2402234	higher potency	higher potency	n.d.	reduced	higher potency	n.d.	n.d.
FLI-06	active, potency n.d.	active, potency n.d.	n.d.	n.d.	n.d.	n.d.	n.d.
LY-450139	active, potency n.d.	active, potency n.d.	n.d.	n.d.	n.d.	n.d.	n.d.

Supplemental Figure 9:

Overview of other Notch inhibitors in comparison to compound C in the various assays used in this study. Context dependent refers to differences between cell lines. Condition dependent refers to results obtained in different cell lines and concentrations, n.d. = not determined.

Supplemental Figure 10
A

uncropped western blot images corresponding to Figure 1F

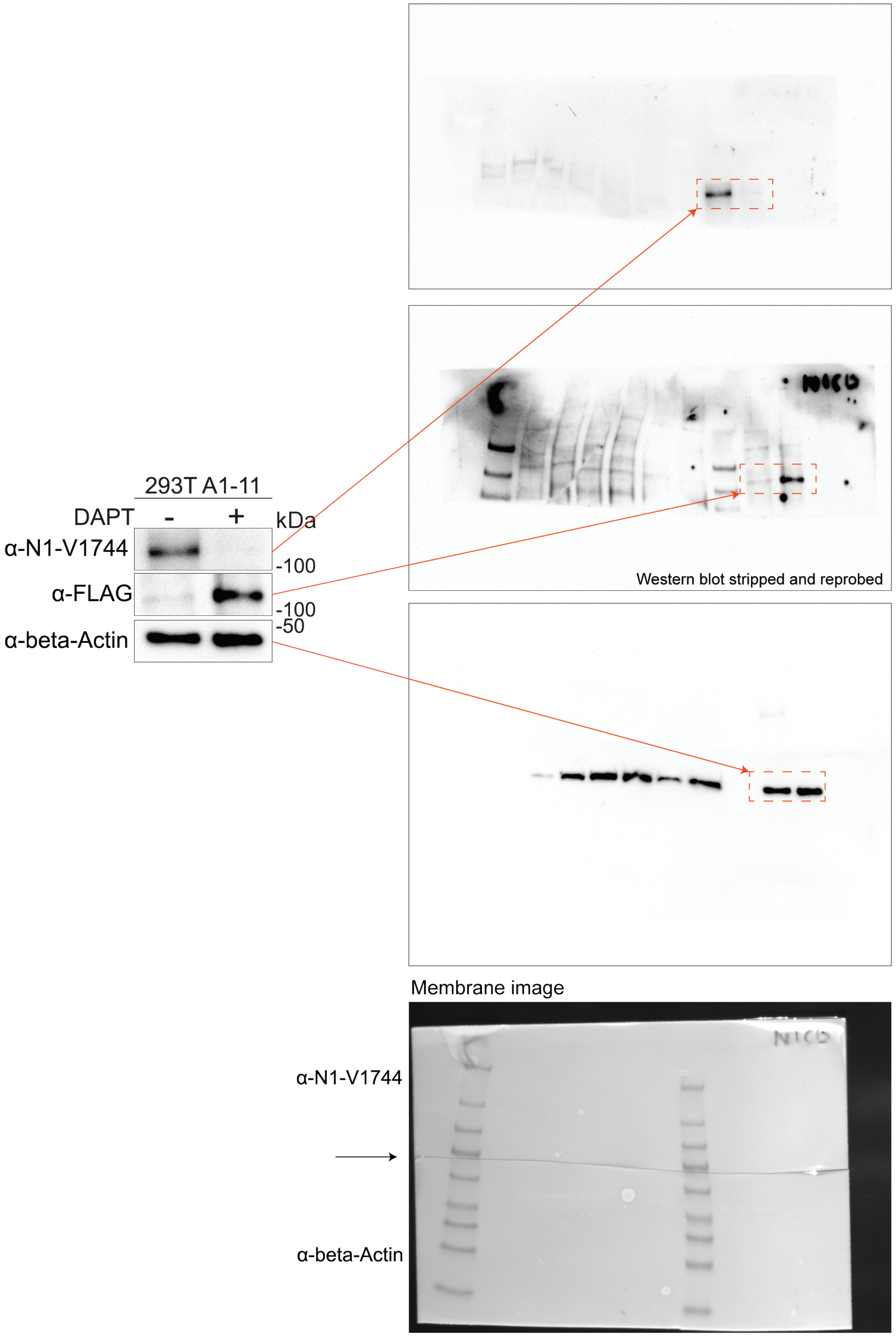

Supplemental Figure 10 continued
B

uncropped western blot images corresponding to Supplemental Figure 4K

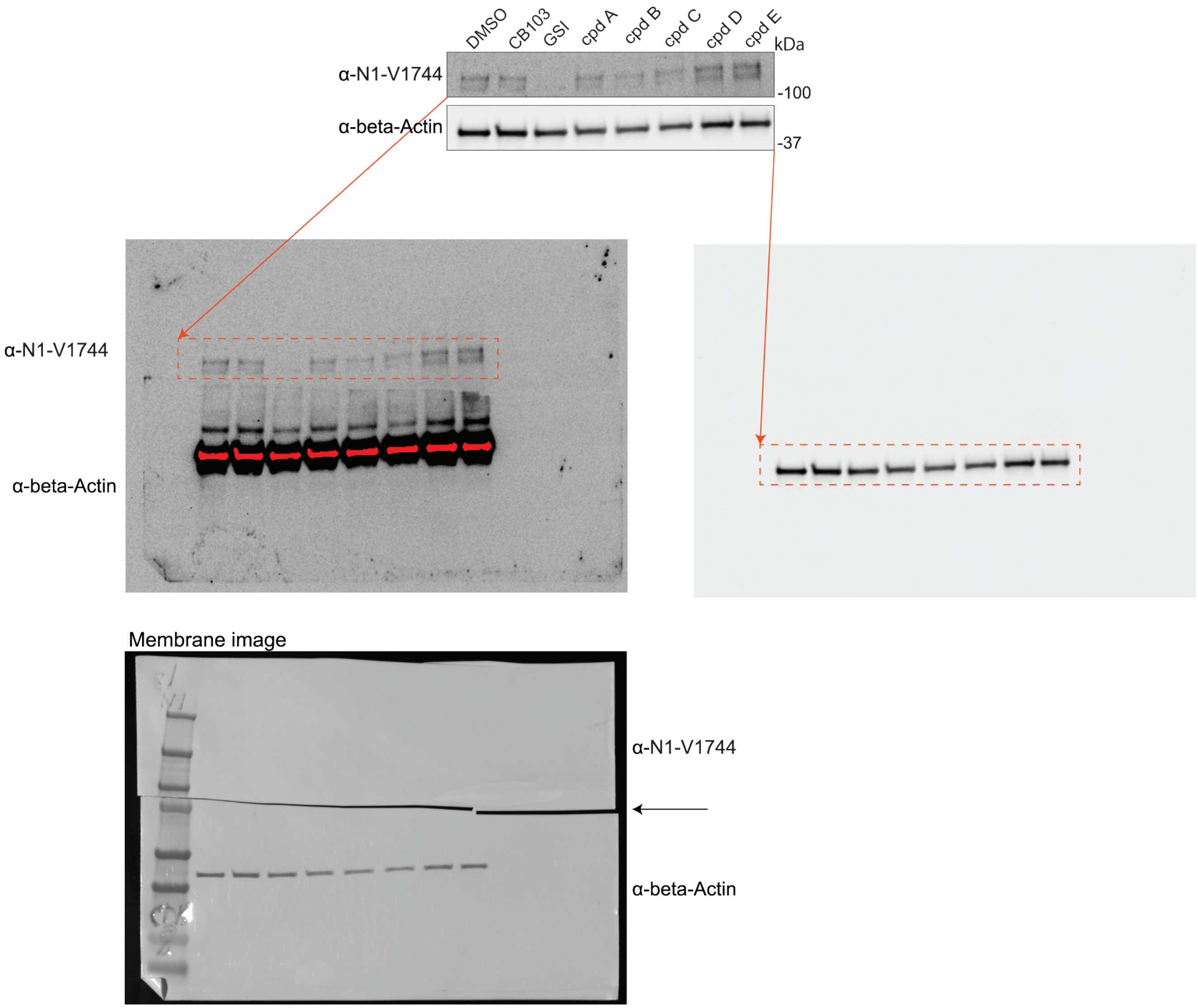

Supplemental Figure 10 continued
C

uncropped western blot images corresponding to Figure 8B

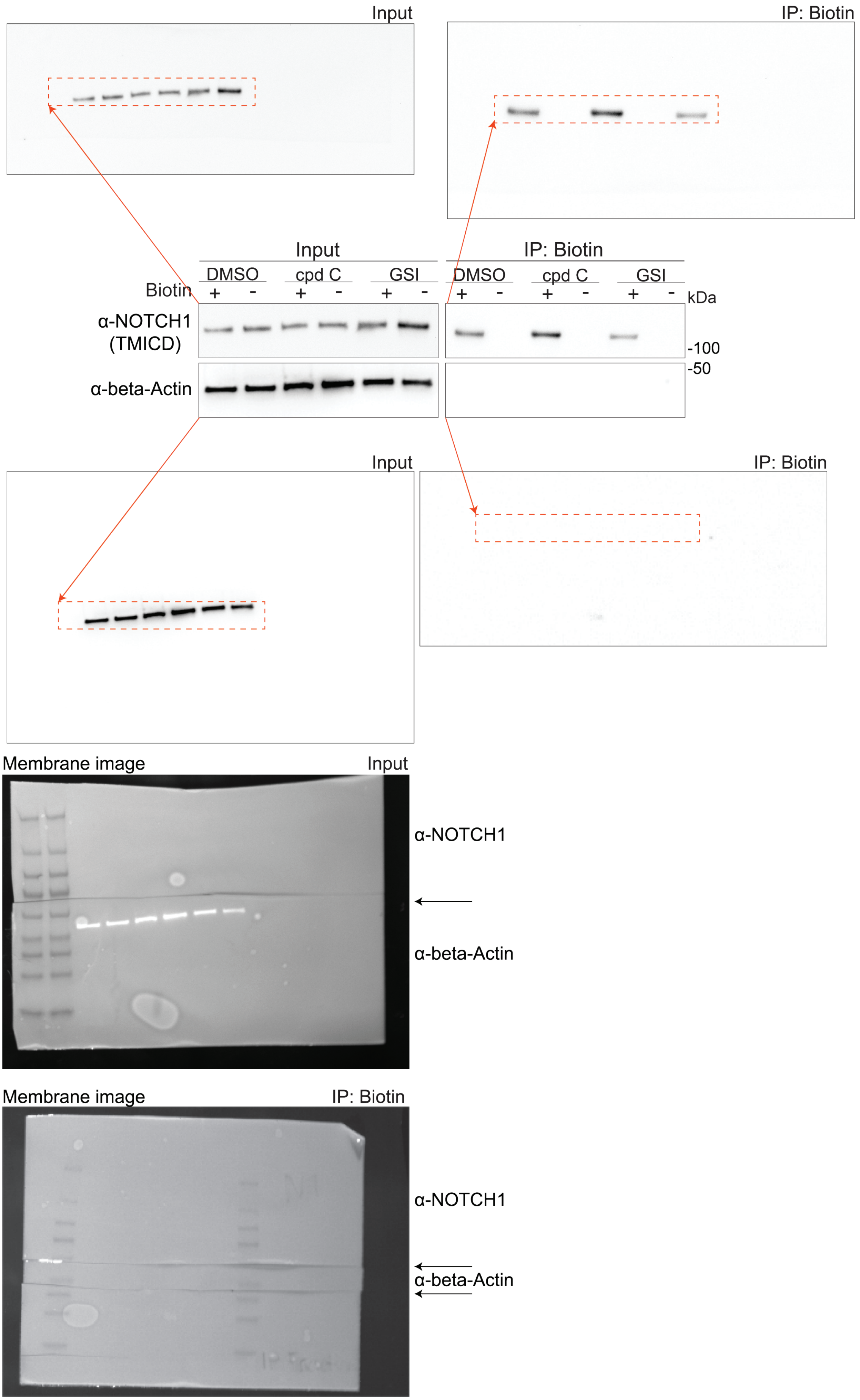

Supplemental Figure 10 continued

D

i) uncropped western blot images corresponding to Figure 8C

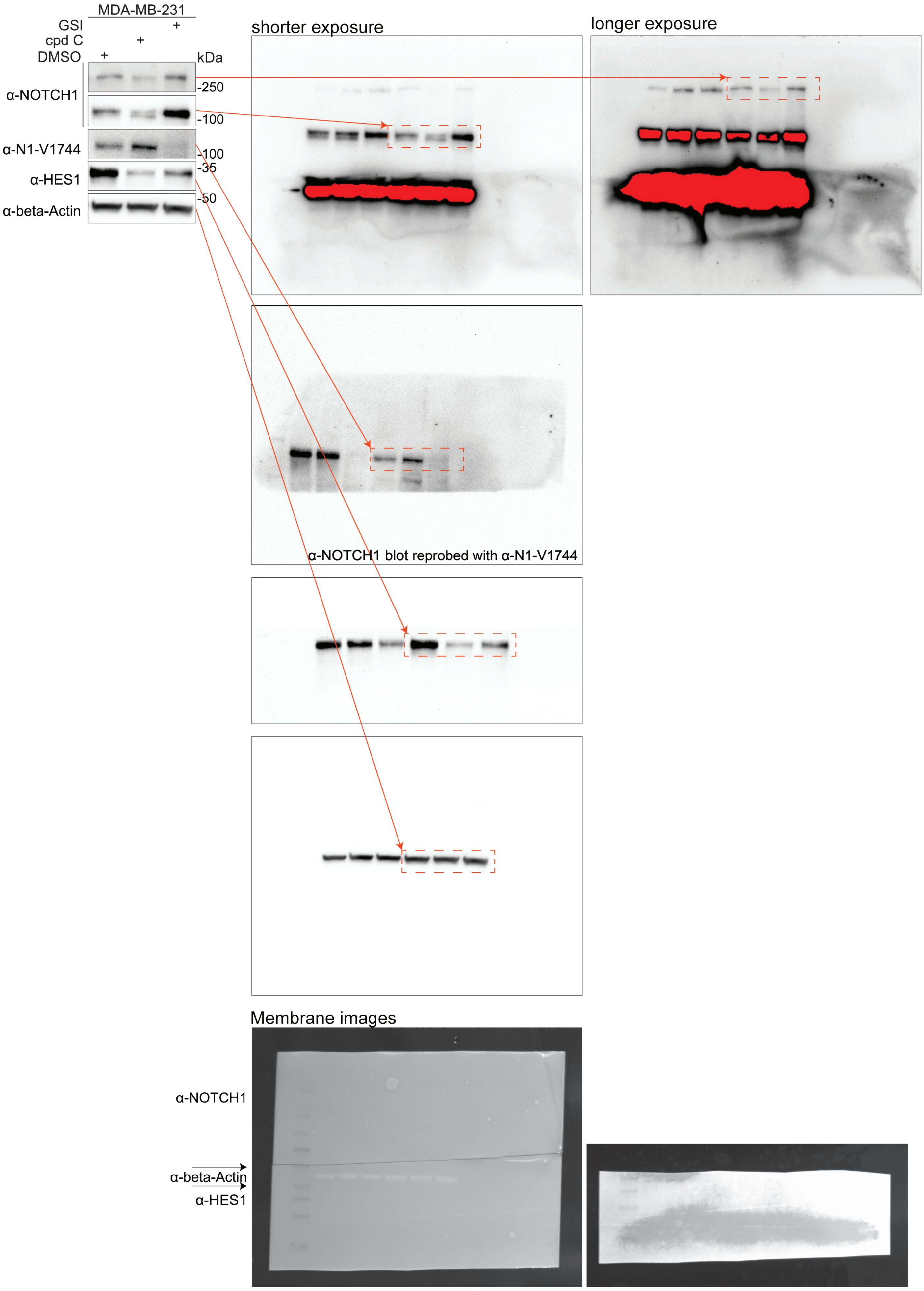

Supplemental Figure 10D continued

ii) uncropped western blot images corresponding to Figure 8C

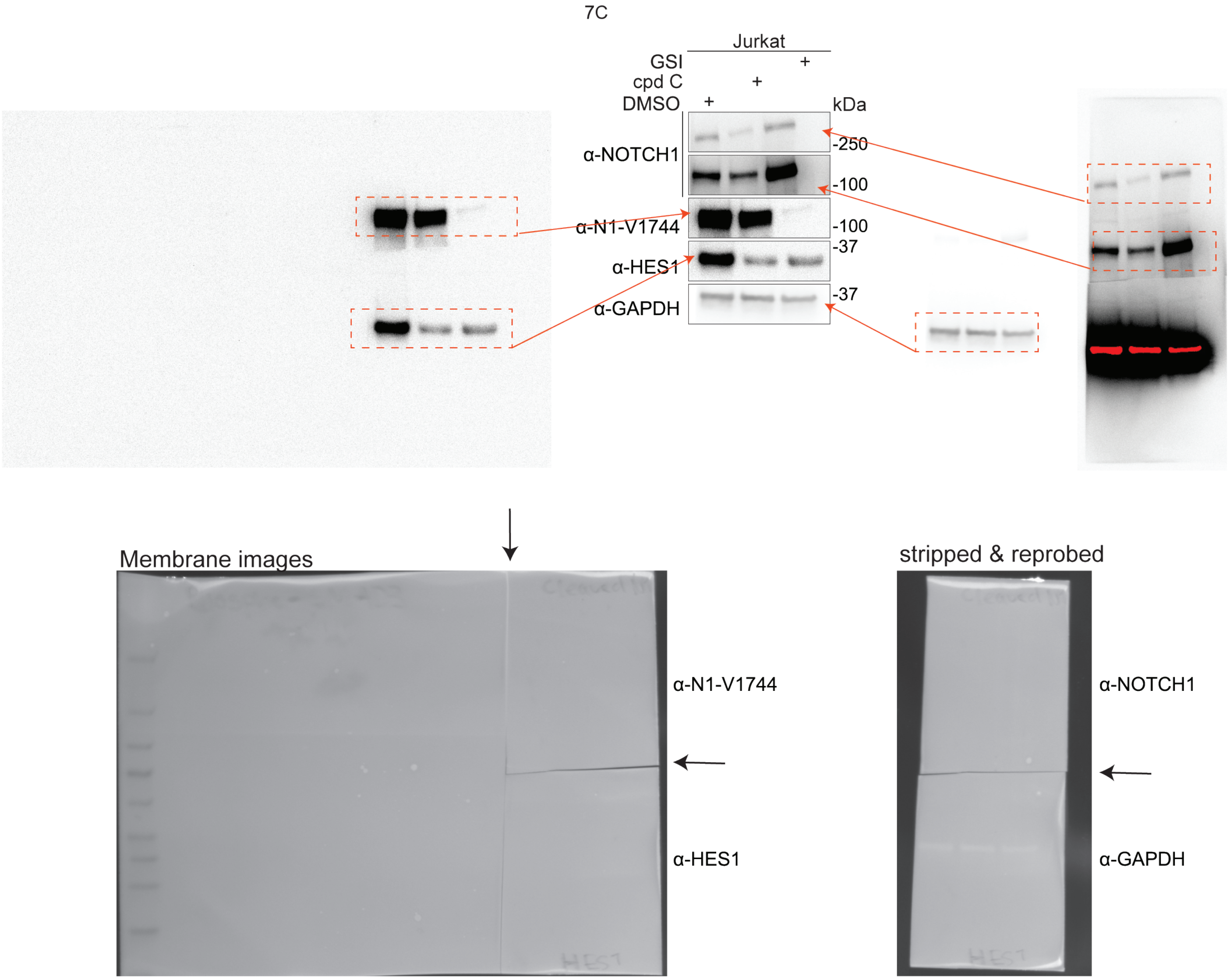

Supplemental Figure 10 continued

E

uncropped western blot images corresponding to Supplemental Figure 8B

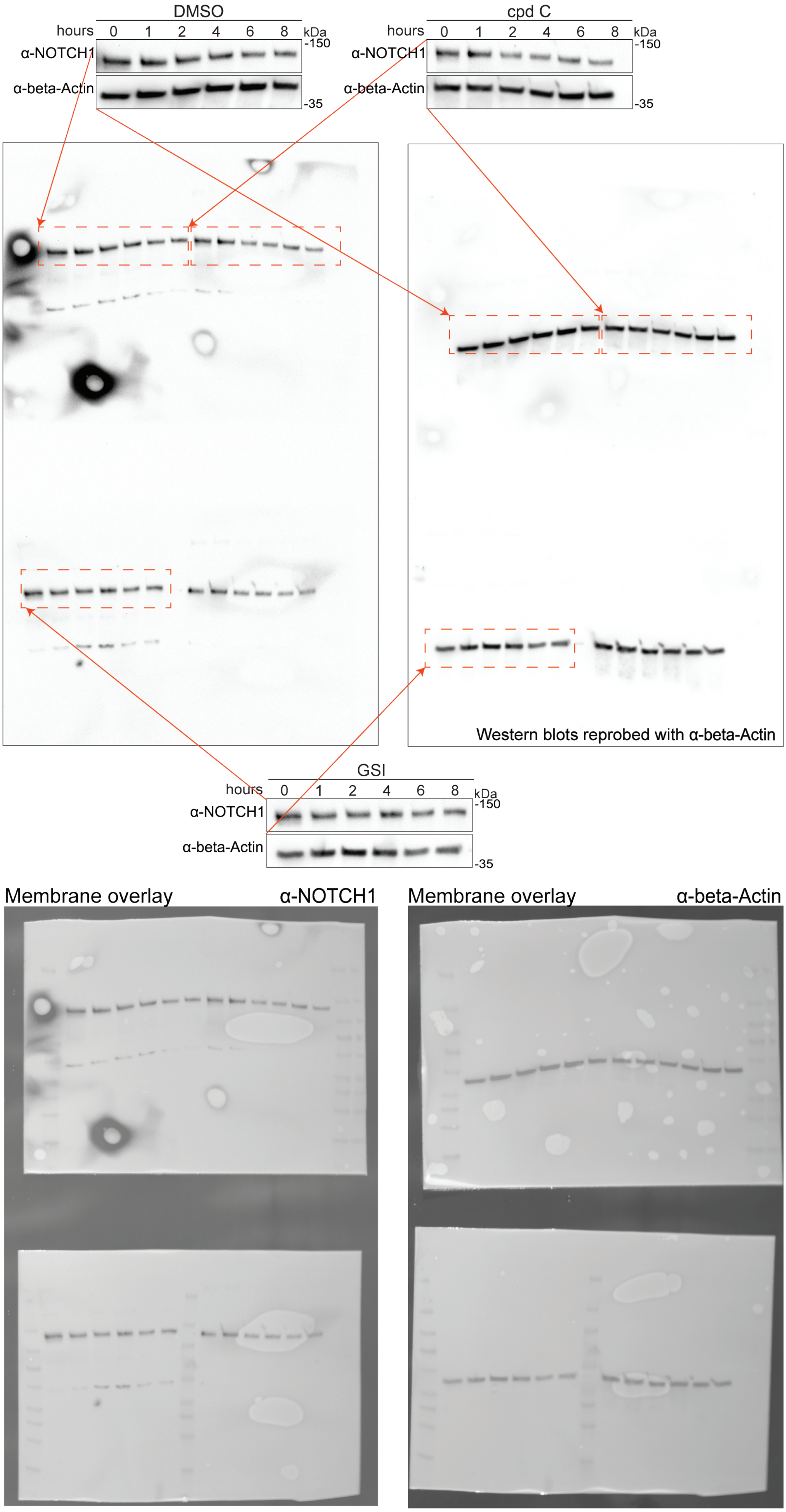

Supplemental Figure 10:

Uncropped versions of western blots. (A) Uncropped version of the western blot presented in Figure 1A. The blot in Figure 1A is shown to the left, and to the right are the corresponding uncropped western blots, with a dashed red box indicating the cropped area presented in the main figure. Other lanes in the uncropped western blot refer to experiments not presented in this study. Below, a bright field view of the gel for the blot is presented (denoted membrane image). The arrow indicates the position where the membrane was cut, and the upper part was used for staining for anti-N1-V1744 and the lower part for a-beta-actin staining. After staining for anti-N1-V1744 the upper part was stripped and reprobed with anti-FLAG M2 antibody. (**B**) Uncropped version of the western blot presented in Supplemental Figure 4K. The arrows indicate the position where the membrane was cut. The upper part was probed with anti-N1-V1744 and the lower part with a-beta-actin antibodies, as indicated. (**C**) Uncropped version of the western blot presented in Figure 8B. The blot in Figure 8B is shown in the middle, and the corresponding uncropped western blots are shown above and below, with red dashed boxes indicating the cropped areas presented in the main figure. Below, two bright field views of the gels for the blots are presented (denoted membrane image). The arrows indicate the position where the membrane was cut. In both images, the upper part was probed with anti-NOTCH1 and the lower part with a-beta-actin, as indicated. The lowest part of the lower figure was used for an experiment unrelated to data presented in this study. (**D**) i) Uncropped version for the western blot for MDA-MB-231 cells presented in Figure 8C. The blot in Figure 8C is shown to the left. To the right the longer and shorter forms of NOTCH1 identified by an anti-NOTCH1 antibody are presented, with short and long exposures for the upper band. Red areas in the western blots indicate signal saturation. The same membrane that was first used for anti-NOTCH1 staining was subsequently cut (marked by an arrow in the bright field image), and the upper part was reprobed with anti-N1-V1774 (second image from top), while the lower part was reprobed with an a-beta actin antibody (fourth image from the top), as control. After developing the blot, the anti-beta-Actin membrane was cut and the lower part probed with anti-HES1 antibody. At the bottom, the bright field view of the gel is shown (denoted membrane images), with arrows marking where the membrane was cut for the different stainings. To the right, the part of the membrane probed with anti-HES1 antibody is shown. ii) The corresponding western blot for Jurkat cells presented in Figue 8C is shown at the top. To the right the cleaved form of NOTCH1 identified with anti-N1-V1774 is presented and detection of HES1 with an anti-HES1 antibody is shown at the lower part of the membrane. The same membrane was stripped and reprobed with anti-GAPDH and anti-NOTCH1 antibody (image to the right). At the bottom, the bright field view of the gel is shown (denoted membrane images), with arrows marking where the membrane was cut for the different stainings. Lanes left of the vertical arrow in the uncropped western blot refer to experiments not presented in this study. (**E**) Uncropped version for the western blots presented in Supplemental Figure 8B. The blots in Figure 7F are shown at the top and lower right. To the left, the western blot after probing with NOTCH1 is shown, and to the right, the same blot after stripping the NOTCH1 antibody and reprobing with a-beta actin. At the bottom of the figure, overlay images of bright field views and blots are presented with the anti-NOTCH1 overlay to the left and a-beta-Actin overlay to the right (denoted membrane overlay).
