# Supplementary material for: Disrupting Notch signalling by a small molecule inhibiting dihydroorotate dehydrogenase activity
Source: Sci Rep. 2026 Jun 6;16:17538. doi: 10.1038/s41598-026-55679-3 (PMC13242511; doi:10.1038/s41598-026-55679-3)
Supplement: Supplementary file 2 — Supplementary Information 2. [file 41598_2026_55679_MOESM2_ESM.pdf]

Supplemental Tables

Supplemental Table 1:
List of 189 potential inhibitor compounds and levels of inhibition in the Notch reporter assay at three different doses: 1.25; 10 and 25 µM. Ranking of inhibitors compared to the primary screen (corresponding to Figure 2A,B).

Supplemental Table 2:
List of potential inhibitor hits and their response in the APP-C99 counter assay in comparison to the 3-point dose-confirmation assay at three different doses: 1.25; 10 and 25 µM (corresponding to Figure 3B; Supplemental Figure 3C).

Supplemental Table 3:
List of 39 potential inhibitors after screening through an 11-point dose-response APP-C99 counter-assay (corresponding to Supplemental Figure 3D,E).

Supplemental Table 4A:
List and chemical structures of the 16 compounds and 47 analogues with Structure (SMILES), Structure image and performance in primary/confirmation screen as well as in the 14-point dose response APP-C99 counter screen.

Supplemental Table 4B:
List of 63 potential inhibitor hits and analogues hit compounds and their response to the APP-C99 counter-screen (Hill-slope and IC50 values) using a 14-point dose-response regimen (corresponding to Supplemental Figure 4A,B).

Supplemental Table 4C:
Summary of IC50 and area under curve (AUC) values for known Notch inhibitors in 9-point and 14-point Notch dose optimisation assays. (corresponding to Supplemental Figure 4 E,F,G).

Supplemental Table 4D:
List of 52 (15 candidate inhibitor hits and 37 analogues) and their response to the Notch reporter-screen using a 14-point dose-response regimen (corresponding to Supplemental Figure 4E,F).

Supplemental Table 4E:
Area under curve (AUC) analysis of data from the Notch reporter and APP-C99 counter assays (corresponding to Supplemental Figure 4I).

Supplemental Table 4F:
Comparison of approximated IC50 values of hits and Notch inhibitors from 14-point dose response screens in the Notch reporter and APP-C99 counter assays (corresponding to Supplemental Figure 4I).

Supplemental Table 4G:
Densitometric measurements of western blot band intensities from the compound treatment experiment presented in Supplemental Figure 4K.

Supplemental Table 5:
Mean fluorescence intensity (MFI) and fusion index from the myogenic assay in C2C12 cells in response to compound treatment (corresponding to Figure 5B,C).

Supplemental Table 6:
IC50 and AUC values for compound A-E, analogues and control inhibitors in CellTiterGLO and HiBit assays (corresponding to Supplemental Figure 5A,B,C,D).

Supplemental Table 7A:
IC50 and Hill-slope values for compound A-E and control inhibitors in the enzymatic DHODH assay (corresponding to Figure 7B,D).

Supplemental Table 7B:
IC50 and Hill-slope values for compound A-E and BAY-2202234 over extended dose in the Notch reporter assay with expanded dose-range (corresponding to Figure 7A and Supplemental Figure 7B).

Supplemental Table 8A:
Calculation of mean MFI and fusion index of MDA-MB-231 cells in response to compound treatment for 72 hours (corresponding to Supplemental Figure 8A)

Supplemental Table 8B:
Calculation of nuclei and giant cell count of MDA-MB-231 cells in response to compound treatment (corresponding to Figure 8C).

Supplemental Table 8C:
Densitometric measurements of western blot band intensities from cycloheximide experiment from compound-treated cells (corresponding to Supplemental Figure 8B).

Supplemental Table 8D:
IC50s and Hill-slopes values from HiBit assays (corresponding to Supplemental Figure 8D,G)

Supplemental Table 9:
List of all antibodies and primers used in this study.
